# Synthesis of Some Mono- and Disaccharide-Grafting Phthalazine Derivatives and Some New *Se*-Nucleoside Analogues: Antibacterial Properties, Quantum Chemical Calculations, and Cytotoxicity

**DOI:** 10.3390/molecules28010317

**Published:** 2022-12-30

**Authors:** I. E. El-Shamy, E. Hleli, A. A. Alsheikh, M. A. Yawer, M. A. El-Hashash, J. Dybal, A. M. Abdel-Mohsen

**Affiliations:** 1 Chemistry Department, Faculty of Science, Fayoum University, Fayoum 63514, Egypt; 2 Institute of Macromolecular Chemistry, Czech Academy of Sciences, Heyrovského nám. 2, 162 06 Prague, Czech Republic; 3 Department of Chemical Engineering, Faculty of Chemical and Petroleum Engineering, Albaath University, Homs 12574, Syria; 4 Department of Chemistry, Division of Science and Technology, University of Education Lahore, Lahore 32200, Pakistan; 5 Chemistry Department, Faculty of Science, Ain Shams University, Cairo 11566, Egypt

**Keywords:** mono/disaccharides, nucleoside analogues, *S-*nucleosides, antibacterial, biocompatibility, quantum calculations

## Abstract

A highly efficient and versatile synthetic approach for the synthesis of 4-(pyren-1-ylmethyl)-1-(d-glycosyloxy) phthalazine nucleosides **11a**,**b**, **13**, *β*-*S-*nucleosides **16**, **18**, **20**, and acyclo *C-*nucleosides **23a**,**b**, **24**, **25** and **27a**–**f** was described and fully characterized. Furthermore, a series of desired new nucleoside analogues containing Se of 4-(pyren-1-ylmethyl) phthalazine-1(2*H*)-selenone **28**–**33** were synthesized. The structures of all reported compounds were confirmed by IR, ^1^H-NMR, ^13^C-NMR, MS and elemental analysis. All compounds have been screened for their antibacterial and antifungal activities. Maximum activity was shown by **20** and **33a** comparable to the standard drugs with lower toxicity. The cytotoxicity of the selected compound was measured and evaluated. The energy gap between the highest occupied molecular orbital and lowest unoccupied molecular orbital was calculated using theoretical computations to reflect the chemical reactivity and kinetic stability of the synthesized compounds. Using density functional theory (DFT), electronic parameters such as the highest occupied and lowest unoccupied molecular orbitals (HOMO and LUMO) and the molecular electrostatic potential (MEPS) were calculated. On the basis of different studied structures, these properties were computed in order to elucidate the chemical reactivity and the kinetic stability. Obviously, the band gap energy (Eg) of structures studied reveals that the lowest band gap obtained for the structure **16**-**a** indicates that it has the highest chemical reactivity and lowest kinetic stability.

## 1. Introduction

Nucleoside analogs have proven to be very useful as a therapeutic drug, including various antiviral products used against viral infections in various cells [1]. In recent years, nucleosides have attained great importance for medicinal chemists because of the discovery that structural modification of natural derivatives can offer important synthetic routes for the preparation of new analogues of bioactive nucleosides. Sugar moieties as well as heterocyclic bases have been modified for certain purposes [2]. Moreover, many nucleoside analogues containing modified sugar showed useful chemotherapeutic activities [3]. For example, hairy cell leukemia and metastatic breast diseases can be cured by using *N*-nucleoside and *C*-nucleoside, respectively. Recently, some *S-*glycosides have been shown to be potential anticancer agents against many cell lines [4,5,6,7,8,9,10]. On the other hand, antimicrobial [11,12,13,14,15,16,17], antitumor [18,19], antihypertensive [20], antitrypanosomally [21], antithrombotic [22], antiallergic [23,24], and anti-inflammatory [12] characteristics shown by phthalazine derivatives used as effective chemotherapeutic agents. Various substituents are incorporated into the phthalazine nucleus, and their fusion with various heterocyclic systems made them biological active compounds. Some 1,4-disubstituted phthalazine derivatives show antitumor activities at concentrations slightly below cytotoxic levels [18]. Others possess significant to mild anti-inflammatory activity [12] and antimicrobial properties [11]. Substitution at the positions 1 and 4 proved to be effective in reducing the cytotoxicity of the compounds. As a result, the attachment of a sugar moiety at position-1 and an alkyl or aromatic substituent at position-4 of the phthalazine nucleus increased its biological activity as a result of the hydrophilic nature of the sugar moiety that can increase its transport to biological systems. Currently, quantum computation studies have become effective methods for verifying the properties of the optoelectronic structure of the active layer used for different areas [25,26]. Density functional theory or other ab initio calculations are among the main drivers used to understand the structural properties of active layers. In addition, they are used to conduct a relative study of their experimental and theoretical analysis based on the same composition [27]. Therefore, this study focuses on the synthesis of *S-* and acyclo *C-*nucleosides of 4-(pyren-1-ylmethyl) phthalazine and some new *S-*containing nucleoside analogues with the aim of producing compounds that have improved biological activity. For this purpose, DFT calculations were performed to better understand their main characteristics, to improve the design, and to examine their electronic properties. The main aim of this study is to synthesize new nucleoside based-phthalazine derivatives. Investigate the biological (antimicrobial and biocompatibility) properties of the synthesized compounds agents’ different types of bacteria and fungi. The chemical reactivity and kinetic stability of the synthesized compounds are calculated and discussed.

## 2. Results and Discussion

### 2.1. Synthesis of Different Intermediates

**Scheme 1:**
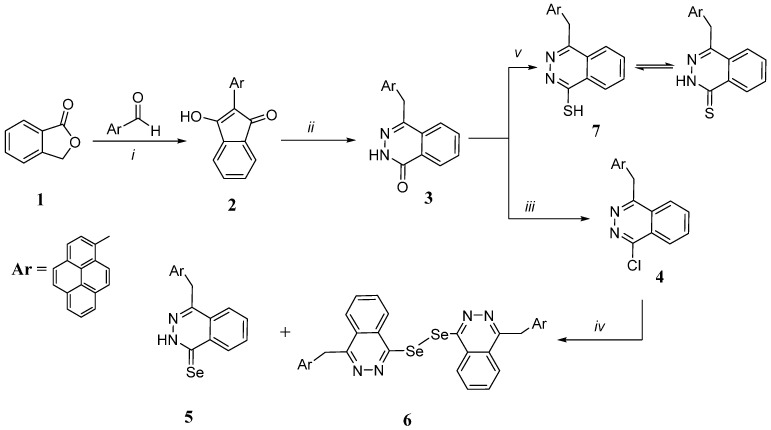
Synthesis of derivatives of pyren-1-ylmethylphthalazine **3**, **5**, and **7**. Reagents and conditions: (*i*) CH_3_ONa; (*ii*) NH_2_NH_2_, EtOH; (*iii*) PCl_5_, POCl_3_; (*iv*) NaBH_4_, Se, ethanol; (*v*) P_2_S_5_, *p*-xylene.

The critical intermediate phthalazinone **3** is synthesized by reaction of phthalid **1** with pyren-2-carboxaldehyde using CH_3_ONa followed by cyclization of indenone **2** with hydrazine hydrate. The ^1^H-NMR spectrum of **3** exchangeable signal of D_2_O in the downstream region is shown at 11.33 ppm, which confirms its presence assigned to the -NH proton of the phthalazine moiety in addition to CH_2_ that appeared as a singlet at 4.69 ppm; its mass spectrum showed the peak of molecular ions at *m/z* 360 corresponding to the molecular formula C_25_H_16_N_2_O. Chlorination of phthalazinone **3** in a mixture of POCl_3_ and PCl_5_ gives chlorophthalazine **4**, which is a good intermediate for the synthesis of various selenium-bearing nucleoside analogs. The IR spectrum of compound **4** showed that there is no absorption for the CO and NH groups. Phthalazine-1(2*H*)-selenone derivative **5** was effectively obtained in excellent yields (85%) by refluxing chlorophthalazine **3** with NaBH_4_/Se in ethanol. The **6** (10%) diselenide derivative **6** (10%) was obtained as a by-product. It was noticed that the inherent capacity for the oxidation of a selenone **5** into diselenide **6** could be accelerated in the presence of air and heating [11]. Selenone **5** and diselenide **6** were separated with the help of fractional crystallization from absolute methanol. When compound **3** was treated with phosphorus pentasulfide, it provided phthalazine-1(2*H*)-thione **7** that was characterized by the elemental analysis technique and spectral data (Scheme 1). The IR spectrum of **7** showed strong absorption bands at 2410 (SH of the thiol tautomeric structure), 1323 (C=S of both thione tautomeric structures) cm^−1^, with the disappearance of the C=O group band of phthalazinone **3**. The mass spectra of **5** and **7** showed characteristic M^+^ peaks with an intensity of 15 and 10%, respectively.

**Scheme 2:**
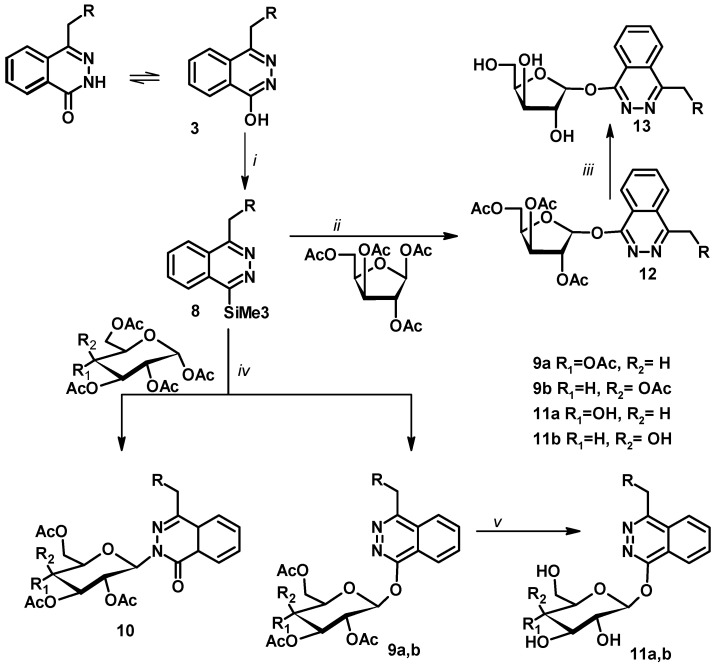
Synthesis of 4-(pyren-1-ylmethyl)-1-(d-glycosyloxy) phthalazine nucleosides **11a**,**b** and **13**. Reagents and conditions: (*i*) HMDS, (NH_4_)_2_SO_4_; (*ii*) 1,2,3,4,6-penta-*O*-acetyl-*α*-d-glucopyranose or α-d-galactopyranose pentaacetate, SnCl_4_, ethylene chloride; (*iii*) *β*-d-ribopyranosetetraacetate, (i*v*) SnCl_4_, (*v*) Et_3_N, MeOH.

Phthalazinone, as with pyradizinone, exists in the lactam–lactim equilibrium, with the lactam tautomer being the most prevalent. The conversion from lactam to lactim form takes place during the formation of the salt with metal ions, where the metal ion was located in the *O*- rather than *N*-atom, regardless of the electropositive nature of the metal ion [28] (Scheme 2). Treatment of phthalazinone **3** with hexamethyldisilazane in the presence of (NH_4_)_2_SO_4_ as a catalyst gave the trimethylsilyloxyphthalazinone derivative **8** containing the respective nucleoside by coupling with per-*O*-acetyl sugars through activation with SnCl_4_ as a catalyst to give the acetyloxonium ion with the formation of SnCl_4_OAc^−^. The oxynium ion is attacked by silylated phthalazine, since a nucleophile on the top side would then give the *β-* anomer exclusively [28,29,30].

Thus, the reaction of trimethylsilyloxyphthalazine with 1,2,3,4,6-penta-*O*-acetyl-*α*-d-glucopyranose or α-d-galactopyranose pentaacetate in the presence of SnCl_4_ resulted in the formation of tetra-*O*-acetylated-*β*-d-glucopyranosyloxy derivatives **9a**,**b** tetra-*O*-acetylated-d-glucopyranosyloxy, respectively (Scheme 2). Deacetylation of compounds **9a**,**b** in the presence of triethylamine/methanol and few drops of water tend to produce free glycosides **11a, b**, respectively. Similarly, coupling of **3** with *β*-d-ribopyranosetetraacetate gave **12** and deacetylation gave the corresponding glycosides **13** (Scheme 2). The IR spectrum of compounds **11a, b,** and **13** showed the absence of the carbonyl amide band, indicating that glycosylation takes place on the *O*- and not on the *N*- atom of the aglycone of **8**. The anomeric proton H-1′ for **9a, b** has appeared as a doublet at *δ* 5.60 and 5.62 having a *J*1′,2′ coupling constant *J*1′,2′ at 9.0 and 8.8 Hz, and it was a correlation with the chemical change for anomeric carbon at *δ* 99.4 and 99.6 ppm, respectively, according to [11,31] with the *β*-configuration. On the other hand, the smaller value of the coupling constant of **12** could be due to the *α*-configuration [32]. The ^1^H NMR data of the deacetylated nucleosides **11a**, **b** and **13** confirmed the absence of acetoxy protons at *δ* 1.59–2.30 with the appearance of exchangeable OH protons with D_2_O at *δ* 4.21–5.49.

**Scheme 3:**
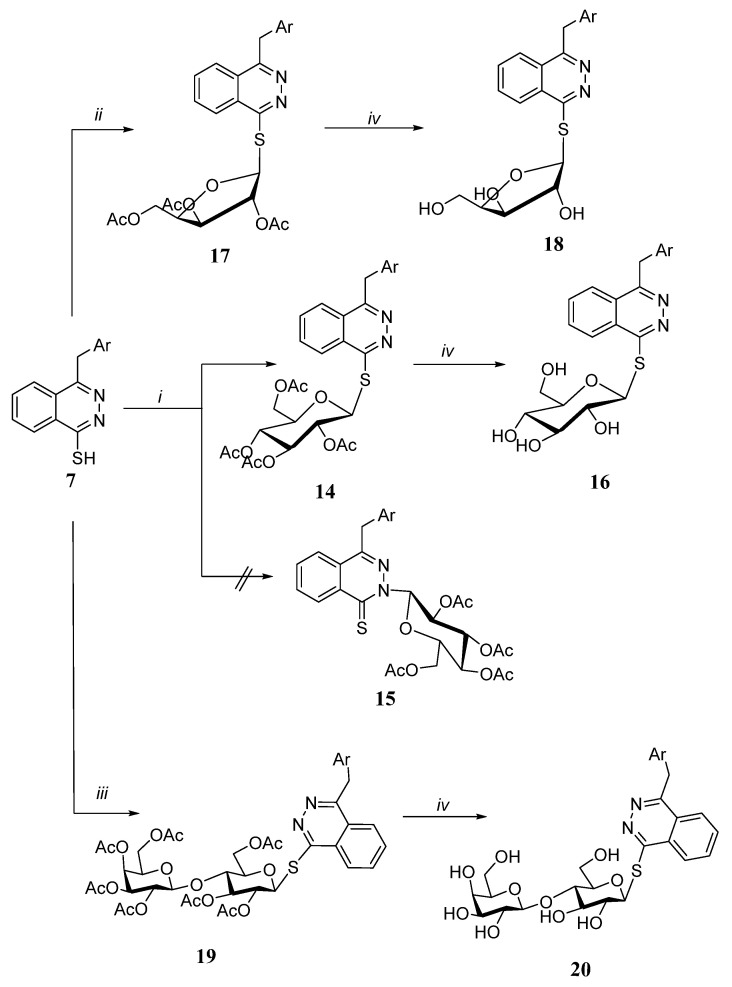
Synthesis of *S*-nucleosides **16**, **18**, and **20**. Reagents and conditions: (*i*) 1-bromo-*α*-d-glucose tetraacetate, Et_3_N, acetone; (*ii*) tri-*O*-acetyl-*α*-d-xylofuranoside bromide, Et_3_N, acetone; (*iii*) acetobromo-*α*-d-lactose, K_2_CO_3_, acetone; (*iv*) MeOH/NH_3_.

The glycosylation of the 7 phthalazinethione derivative with one equivalent of 1-bromo-*α*-d-glucose tetraacetate in dry acetone/triethylamine bears the corresponding *S-*nucleoside **14** (Scheme 3). The *S-β*-d-configuration of compound **14** was assigned from their ^1^H NMR data, which confirmed the presence of anomeric proton H-1′ of the glycoside moiety as a doublet at δ 5.59 with a *J*1′,2′ coupling constant of 11.02 Hz and its correlation with the chemical change for anomeric carbon at *δ* 90.4 ppm consistent with the reported data for *S-β*-d-glycosides [32]. Similarly, *S-*nucleoside **17** was synthesized by reacting phthalazinethione **7** with tri-*O*-acetyl-*α*-d-xylofuranoside bromide. The ^1^H NMR spectrum of **17** showed the characteristic xylofuranosyl pattern, with a doublet signal at 6.08 ppm for the anomeric proton, as observed for analogous *β*-d-xylofuranosyl nucleosides [33].

The other protons in the glycoside moiety resonated at δ 3.80–5.59, while the three acetyl groups appeared as three singlets at *δ* 1.99, 2.19, and 2.33 ppm. Deacetylation of *S-*nucleosides **14** and **17** using NH_3_/MeOH at 25 °C bearing the free glycosides **16** and **18**, respectively. The ^1^H NMR data of the latter compounds revealed the absence of acetyl protons with the appearance of exchangeable D_2_O OH protons at *δ* 3.50–5.61. Furthermore, the reaction of phthalazinethione **7** with acetobromo-*α*-d-lactose in anhydrous acetone/K_2_CO_3_ gave *S-*nucleoside **19**. The deacetylation of that resulted in the formation of the corresponding *S-*lactoside **20** (Scheme 3). The ^1^H NMR spectrum of acetylated *S-*lactoside **19** showed signals at *δ* 1.90–2.18 assigned to the acetyl groups and a doublet at *δ* 6.40 for the anomeric proton with a *J*1′a,2′a coupling constant of 9.1 Hz indicating the *β*-orientation of the thioglycosidic bond. The ^1^H NMR spectrum of deacetylated *S-*lactoside **20** confirmed the absence of the OCOCH_3_ groups with the appearance of D_2_O exchangeable OH protons at *δ* 4.39–5.34.

**Scheme 4:**
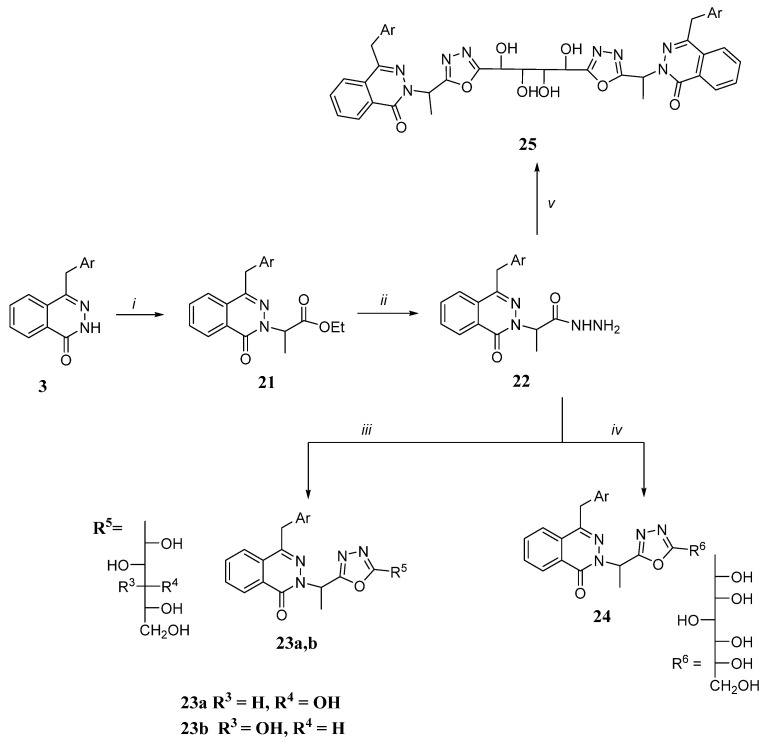
Synthesis of acyclo *C*-nucleosides **23a**,**b**, **24**, and **25**. Reagents and conditions: (*i*) ethyl 2-bromopropionate, K_2_CO_3_, acetone; (*ii*) NH_2_NH_2_, EtOH; (*iii*) d-glucono and d-galactono-1,5-lactone; (*iv*) d-glucoheptonic acid-1,4-lactone; (*v*) diethyl galactarate, pyridine.

Recent studies have shown that 2-(1-Oxo-4-(pyren-1-ylmethyl) phthalazin-2(1*H*)-yl) propanehydrazide (**22**) has been selected for the synthesis of the desired new acyclo *C*-nucleosides (Scheme 4). When phthalazinone **3** was allowed to react with ethyl 2-bromopropionate in dry acetone and in the presence of anhydrous K_2_CO_3_, it produced the corresponding ester derivative **21** in 70% yields. Acid hydrazide **22** was synthesized by refluxing its corresponding ester derivatives **21** with hydrazine hydrate in absolute ethanol. Compounds **21** and **22** were confirmed by IR, ^1^H NMR, and mass spectra, which agree with the assigned structures. Dehydrative cyclization of equimolar amounts of d-glucono and d-galactono-1,5-lactone with hydrazide derivative **22** in absolute ethanol gave acyclo-*C*- nucleoside **23a, b,** respectively (Scheme 4). The reaction occurs via a nucleophilic attack of *N-*1 of the hydrazide 22 on the carbonyl carbon of lactone, which led to an opening of the lactone ring that was followed by dehydrative cyclization of the intermediate obtained to give the acyclo-*C* nucleoside 23a, b. To synthesize analogues with a longer alditolyl residue, reactions have been extended to d-glucoheptonic acid-1,4-lactone, which upon reaction with hydrazide **3** would result in the formation of 2-(1-(5-(d-glycero-d-gulo-hexitol-1-yl)-1,3,4-oxadiazol-2-yl)ethyl)-4-(pyren-1-ylmethyl) phthalazin-1(2*H*)-one (**24**). The ^1^H NMR spectra of the compounds **23a**,**b** and **24** show the absence of protons of NH and NH_2_ at *δ* 9.32 and 4.46 with the appearance of exchangeable OH D_2_O protons from the sugar residue at *δ* 4.20–5.39. The ^13^C NMR spectra of the acyclo *C-*nucleoside **23a** showed 5 signals of sugar carbons resonated in a higher field in the range 64.2–72.2 ppm. Furthermore, this investigation has been extended toward the synthesis of a double-headed acyclo-*C*-nucleoside analogue. Fusion of diethyl galactarate with hydrazide **22** gave the double-headed target acyclo *C* nucleoside **25**. Its ^1^H NMR spectrum showed only two doublets at *δ* 3.89 and 4.90 assigned for H-2′, H-3′ and H-1′, H-4′, respectively, indicating the existence of a center of symmetry [34].

**Scheme 5:**
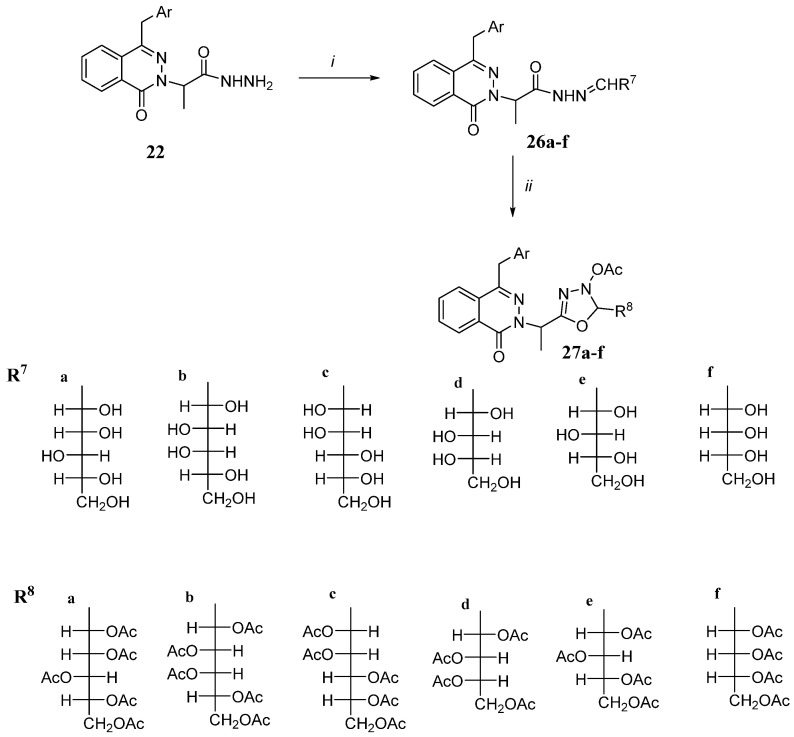
Synthesis of **27a**–**f** acyclo *C*-nucleosides. Reagents and conditions: (*i*) R^7^CHO, EtOH; (*ii*) Ac_2_O.

Hydrazides **22** were condensed with monosaccharides (d-glucose, d-galactose, d-mannose, l-arabinose, d-xylose and d-ribose) in an aqueous ethanolic solution and a catalytic amount of glacial acetic acid, yielded the corresponding hydrazino-sugar derivatives **26a–f**, respectively (Scheme 5). The ^1^H NMR spectrum of the sugar hydrazones **26a–f** (**R**^7^) confirmed the presence of the azomethine proton H-1′ as a doublet in a low field in the range *δ* 6.99–7.04 ppm followed by the rest of the alditol-l-y1 side chain in the higher field, in addition to the NH at *δ* 10.10–11.03 ppm. However, the rest of the sugar and hydroxyl protons appeared as multiples at 3.5–5.3 ppm. The ^13^C NMR spectrum of the sugar hydrazones **26a–f** (**R**^7^) showed five C=N (C-1′) signals in the range 149.5–150.1 ppm, in addition to the sugar residue signals (Scheme 5). The assignment of sugar carbon atoms based on their equivalences of chemical shifts to the open structure assigned to other sugar hydrazones [35]. Therefore, the lowest field signals were assigned to C-4′ or C-5′, and the highest field signal was assigned to C-6′. The resonance of approximately 71.9 ppm is assigned to C-2′. These data confirmed the open-chain nature of the sugar residues of the hydrazones **26a–f**. Cyclization as a result of condensation of sugar hydrazones **26a–f** with acetic anhydride at 130 _°C_ for 1.5 h tended to form the acyclo *C*-nucleosides **27a–f** (**R**^8^). The IR spectrum of compounds **27a–f** (**R**^8^) showed characteristic absorption bands for OAc (1733–1740 cm^−l^) and NAc (1677–1690 cm^−l^), devoid of any OH and NH bands. The presence of protons of the COCH_3_ group, CH of the oxadiazole moiety (*δ* 5.80–6.07), and the downward shift of the sugar protons in ^1^H NMR spectra confirmed the assignment of the structure of **27a–f**. Moreover, the ^13^C NMR spectra of **27a–f** (**R**^8^) showed oxadiazole-CH at a higher field (*δ* 83.9–86.2 ppm) than the respective carbon (C-1′) in **26** confirmed the cyclization.

**Scheme 6:**
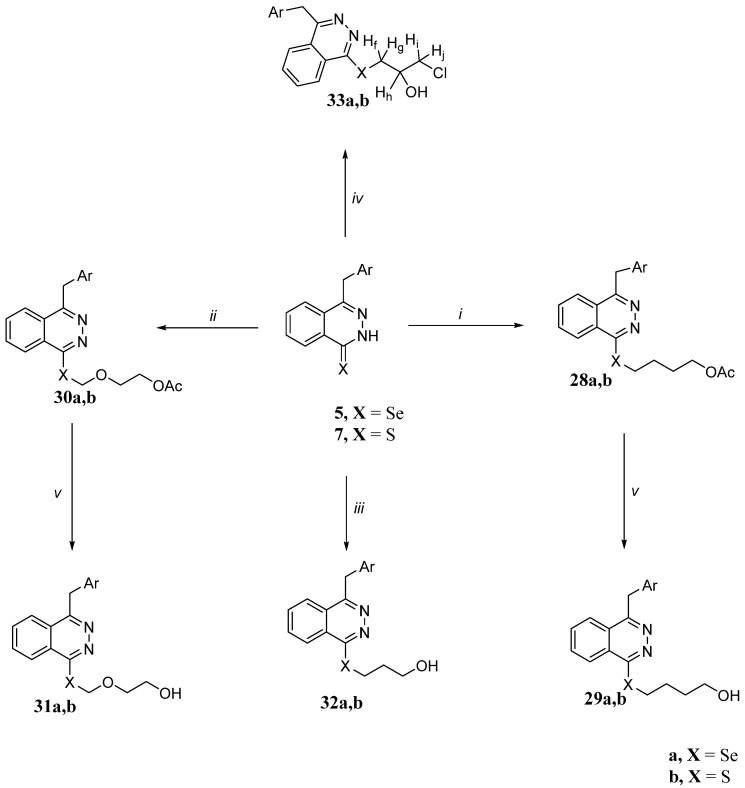
Synthesis of *S*- and *S*-containing nucleoside analogues **28**–**33**. Reagents and conditions: (*i*) 4-bromobutyl acetate, K_2_CO_3_, Et_3_N; (*ii*) 2-acetoxyethoxymethyl bromide, K_2_CO_3_, Et_3_N; (*iii*) 3-chloropropanol, K_2_CO_3_, Et_3_N; (*iv*) 1,3-dichloro-2-propanol, K_2_CO_3_, Et_3_N; (*v*) Et_3_N, MeOH.

Treatment of phthalazine-1(2*H*)-selenone **5** and phthalazine-1(2*H*)-thione 7 with different alkylating agents *viz*., 4-bromobutyl acetate, 2-acetoxyethoxymethyl bromide, 3-chloropropanol, and 1,3-dichloro-2-propanol in boiling *N*,*N*-dimethylformamide in the presence of K_2_CO_3_ formed the corresponding acetyloxy *S*- and *S*-alkyl phthalazines **28, 30, 32** and **33** (Scheme 6). Deacetylation derivatives of compounds **29** and **31** were obtained using the saturated triethylamine-methanol solution and a few drops of H_2_O in the presence of compounds **28** and **30**, respectively. The IR spectra of compounds **28** and **30** revealed that the absorption bands of the acetoxy group at 1739–1747 cm^−1^. Compounds **29** and **31**–**33** showed absorption bands at 3487–3110 cm^−1^ attributable to the OH group. The ^1^H NMR spectrum of **30a** showed COCH_3_ signal at 2.12 ppm in addition to the CH_2_ signals, each as a triplet. The ^13^C NMR spectrum of **32a** showed the expected number of signals for aromatic carbons and four methylene carbon signals at *δ* 23.3–67.2.

### 2.2. Antimicrobial Activity of the Prepared Compounds

From the data analysis of the inhibition zone (Table 1), it was confirmed that the most synthesized compounds exhibited significant inhibitory effect against bacterial and fungal strains than the starting compound **3** which confirmed the improvement of biological properties by building the sugar moiety on the phthalazine ring. Phthalazine derivatives **16**, **18**, **20**, **26a** and **33a** showed good inhibitions against all bacterial and fungal strains related to the reference drugs. The *S-*lactoside **20** and the *S*-nucleoside analog **33a** have been shown to be the best antibacterial agents due to the presence of sugar and selenolochloropropanol moieties, while **16**, **18** and **26a** are good antifungal agents due to the presence of *S-*nucleoside and sugar hydrazine moieties.

### 2.3. Minimum Inhibitory Concentration (MIC)

Minimum inhibitory concentrations (MIC) of the most active phthalazine derivatives **16**, **18**, **20**, **26a** and **33a** against two species of bacteria, *Staphylococcus aureus* (MTCCB 737) and *Escherichia coli* (MTCCB 1652) and also two species of fungi, *Aspergillus niger* and Alternaria alternate, were evaluated using the serial dilution technique [20,28]. Dilution series were prepared with 6.25, 12.5, 25, 50 and 100 mg/mL of nutrient broth medium in each tube, 100 mL of standardized suspension of the test microbes (107 cells/mL) were added and incubated at 37 °C for 24 h. Compounds **20a** and **33a** demonstrated good inhibitions against selected bacterial and fungal strains.

### 2.4. Cytotoxicity Activity

The LC50 values of the newly synthesized compounds **16**, **18**, **20**, **26a** and **33a** were 1.39, 2.31, 3.54, 0.58 and 6.49 mg/mL, respectively. The standard drug Bleomycin has an LC50 value of 0.41 mg/mL (Table 2). The lowest LC50 value was found in sugar hydrazine **26a**, indicating its higher cytotoxicity than the other compounds. The *S*-lactoside **20** and the *S-*nucleoside analog **33a** showed excellent biocidal activity against brine shrimp due to their lower cytotoxicity that satisfied the preliminary antimicrobial screening and MIC (Table 3). So, we can conclude that the *S-*lactosyl and *S-*nucleoside analogs of pyrenylmethylphthalazine are the most active potent antibacterial and antifungal agents with lower toxicity in host cells.

### 2.5. Frontier Molecular Orbitals

With the fully optimized geometry structure, we then obtained the corresponding electronic structures HOMO (highest unoccupied molecular orbital), LUMO (lowest unoccupied molecular orbital) and the band gap energy value (Eg). Furthermore, the molecular electrostatic potential (MESP) maps were calculated by the DFT B3LYP/6–31 + G(d) method. The energy levels HOMO and LUMO are the molecule’s most important orbitals. These orbitals are sometimes referred to as frontier molecular orbitals. The HOMO orbital represents the electron donor and the LUMO orbital represents the electron acceptor. The LUMO–HOMO energy gap was a very effective property for determining the molecule’s interaction with other species and characterizing the chemical reactivity and kinetic stability of the molecule. A molecule with a small frontier orbital gap LUMO–HOMO is more polarizable and was generally associated with high chemical reactivity and low kinetic stability [36]. In contrast, a molecule with a large LUMO–HOMO frontier orbital gap was generally associated with low chemical reactivity and high kinetic stability [36]. The HOMO energy, the LUMO energy, and the LUMO–HOMO energy gap of compounds **1**, **3**, **5**, **7**, **9a**, **16**, **18**, **20**, **24a**, **26a**, **27f**, **29a** and **33a** are listed in Table 4 and Table S1 (Supplementary Materials). Optimized geometric structures, 3D HOMO molecular orbital, and 3D LUMO molecular orbital for the compounds drawn are given in Table 5 and Table S2.

It is observed from Table 4 that compound **1** has the highest energy level of HOMO and the lowest energy level of LUMO. Then, it gives rise to an energy gap Eg of the order of 5.77 eV, so it is less polarizable [37]. However, for a longer-chain structure, the band gap decreased as with compound **16a**, which has a band gap of approximately 3.30 eV. The comparison of LUMO–HOMO energy gaps reveals that compound **1** has the largest value of the LUMO–HOMO energy gap. Therefore, it has the lowest chemical reactivity and highest kinetic stability, while compounds **16a** have the highest chemical reactivity and lowest kinetic stability due to the smallest value of the LUMO–HOMO energy gap (Table S1).

### 2.6. Chemical Reactivity of Descriptors

To assume a qualitative chemical reactivity, we subsequently determined some theoretical descriptors related to the DFT calculations using the values already found of the HOMO and LUMO level. The following parameters are as the ionization potential (*I*), the electron affinity (*A*), the electronegativity (*χ*), global hardness (*η*), softness (σ), chemical potential (*μ*) and the global electrophilicity index (*ω*). The ionization potential (*I*) and electronic affinity (*A*) values were calculated according to the Kooperman theorem [38].

$$I=-E_{HOMO}\;A=-E_{LUMO}$$

Therefore, electronegativity (*χ*), global hardness (*η*), softness (*σ*), chemical potential (*μ*) and global electrophilicity index (*ω*). These descriptors were determined using Equations (1)–(4) [31,39].

(1) $$\chi =-µ=-1/2\left(E_{HOMO}+E_{LUMO}\right)$$

(2) $$\eta =\left(I-A\right)/2$$

(3) $$\omega =\frac{\chi^{2}}{2\eta }$$

(4) $$\sigma =\frac{1}{\eta }$$

These DFT-based chemical reactivity calculations are admissible to forecast a better understanding of chemical systems related to their stability and reactivity. The determined values of the parameters of the chemical characteristics are reported in Table 5 and Table S2 (Supplementary Materials of Table S2).

In general, the chemical reactivity varies with the structure, the electronegativity (*χ*) is the parameter that reflects the ability of a molecule to attract electrons toward itself. Chemical hardness (*η*) and global softness (*σ*) express the resistance of the system to change in its number of electrons. The overall electrophilicity index characterizes the electrophilic power of the molecule. In this case, as shown in Table 5, compound **16a** presents the lowest hardness (*η* = 1.65 eV) and the highest softness (*σ* = 3.22 eV). However, compound 1 presents the highest electronegativity (*χ* = 2.89 eV), the highest hardness (*η* = 2.89 eV) and the lowest softness (*σ* = 3.22 eV). Therefore, compound 16a is a more electron donor, as referred to in [40]. Furthermore, it is noticed as well as that, in the case of the dimer resulting from physical interaction, those characteristic parameters are improved—for example, in the case of dimer 16 a, the electronegativity decreased (*χ* = 3.70 eV), chemical hardness (*η* = 1.45 eV) decreased and the softness (*σ* = 3.54 eV) increased.

### 2.7. Molecular Electrostatic Potential

**Scheme 7:**
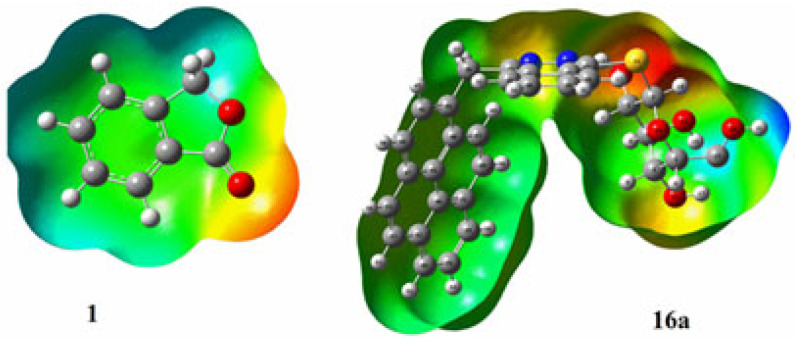
Representative molecular electrostatic potentials (MESP) mapped on the electron density surface calculated by the B3LYP/6–31 + G(d) method for compounds (**1**) and (**16a**). Symbols are: O (red), C (gray), H (white), and N (blue).

The molecular electrostatic potential (MESP) is a useful three-dimensional method that can visualize molecules’ charge distribution and charge-related properties such as dipole moment, electronegativity, and molecular polarity (Scheme 7 and Table S2). Different colors represent the different values of the electrostatic potential on the surface; the red region corresponds to the lowest or most negative electrostatic potential, the blue region identifies the highest or most positive electrostatic potential, and the green region refers to the area of the zero potential. The electrostatic potential increases in the order red < orange < yellow < green < blue (Scheme 7 and Table S2). The molecular electrostatic potential surface mapped onto an electron density on surface also allows us to display molecules’ size, shape, and charge density. In organic chemistry, the molecular electrostatic potential property is widely used to predict the sites of chemical reactivity for electrophilic (electron-rich region) and nucleophilic (electron-poor region) attacks. The MESP region with red color represents the highest reactivity to electrophilic attack, while the blue color is the most suitable site for nucleophilic attack [40].

To predict chemically reactive binding sites for electrophilic and nucleophilic attacks of the **1** and **26a** molecule mentioned above, MESP plots were calculated at the theory level B3LYP/6-31 + G(d). MESP maps, generated with optimized geometry of the **1** and **16a** molecules using Gauss View 05 software, are shown in Scheme 7. It may be seen that for molecule **1**, the negative electrostatic potential region (red and yellow) is mainly localized over the oxygen atoms, especially the oxygen atom of the carbonyl group, which is the most reactive site for an electrophilic attack.

**Scheme 8:**
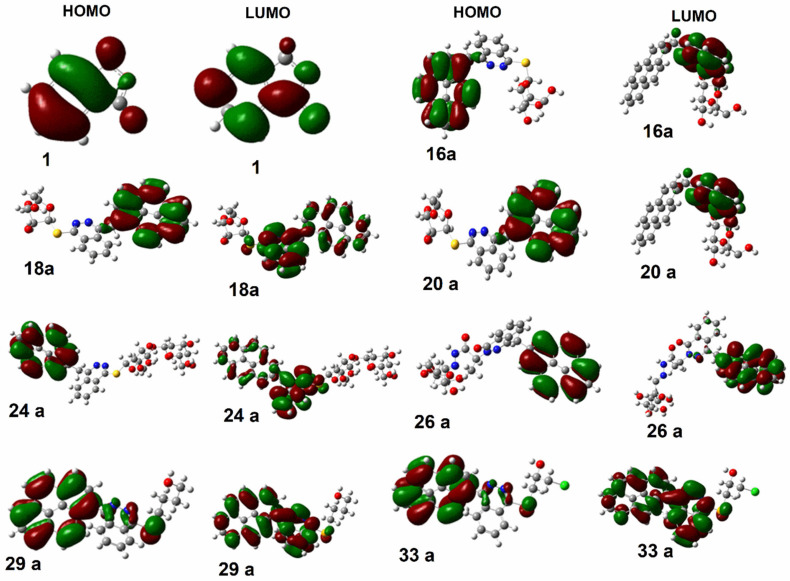
The 3D plots of the molecular orbitals of HOMO and LUMO of some compounds were calculated using the DFT (B3LYP)/6–31 + G(d) method.

The highest positive electrostatic potential region (blue) is located on the carbon atom linked to the oxygen atom, which is the most reactive site for a nucleophilic attack. The map of the electrostatic potential surface of the **16a** molecule shows that the most negative electrostatic potential region (red and yellow) is located mainly in the oxygen atom related to carbon number3, which can be suggested as a possible site for an electrophilic attack. The positive electrostatic potential region (blue) is located on the carbon atoms linked to carbon on the one side and the other on the OH group, which can be considered as possible sites for a nucleophilic attack. As shown in Scheme 8, both HOMO and LUMO are located on one side of the polymer backbone far from the central unit. Practically, in all cases, the HOMO is localized in the Ar group, and the LUMO is localized in the phthalazine group.

Thereby, it can be seen from Scheme 8 and Figure S1 (Supplementary Materials) that in the case of a dimer resulting from a chemical reaction calculated (dimer 1) between two monomers of the same structure, no change is observed in the energy value of the band gap explained by the localization of HOMO–LUMO, which are far from the change occurring in the structure. However, in the case of a dimer formed due to a physical interaction between two monomers placed symmetrically (dimer 2) or asymmetrically (dimer 3), the band gap energy has been reduced compared to the value of the initial monomer. Thus in [41], this decrease in the energy gap value is due to the intermolecular interaction which can arise among the two monomers, such as the hydrogen bonding interaction.

## 3. Material and Methods

### 3.1. Materials

1-Pyrenecarboxaldehyde 99%, Selenium powder, 99.99% trace metal basis, hexamethyldisilazane (HMDS), reagent grade, ≥99%, α-d(+)-glucose pentaacetate 99%, *β*-d-ribofuranose 1,2,3,5-tetraacetate, and acetobromo-α-d-glucose were purchased from Sigma-Aldrich. Acetobromo-α-d-xylose, stabilized with 2.5% CaCO_3_, α-d-Galactose pentaacetate 98%, d-glucono-1,5-lactone and d-glucoheptonic acid-1,4-lactone from Biosynth Carbosynth^®^ (Manchester, UK). acetobromo-α-d-lactose 95% by HPLC, from Creative Biolabs (Shirley, NY, USA). Diethylgalactarate from Bench Chemical, (Huangshan, China). Brine shrimp eggs were obtained from the UniAqua Laboratory in Naawan (Misamis Oriental, Philippines), as a gift sample for cytotoxicity.

### 3.2. Methods

*3-Hydroxy-2-(pyren-1-yl)-1H-inden-1-one* (**2**): A solution of phthalide **1** (0.01 mol) and pyren-2-carboxaldehyde (0.01 mol) in ethylpropionate (60 mL) was added to a stirred solution of sodium ethoxide (200 mL, 0.1 N) at 25 °C. The reaction mixture was then heated to reflux for 1 h with stirring. After cooling, the reaction mixture was poured into ice water, acidified with glacial acetic acid at pH 2–3. The resulting precipitate was filtered off, washed with water, and crystallized from ethanol to give **2**.

*4-(Pyren-1-ylmethyl) phthalazin-1(2H)-one* (**3**): A mixture of indenone **2** (0.01 mol) and 80% hydrazine hydrate (0.05 mol) in 40 mL of ethanol and a catalytic amount of glacial acetic acid was refluxed for 5 h. The reaction mixture was left to cool at room temperature. The resulting solid product was collected by filtration and recrystallized from toluene to give **3**.

*1-Chloro-4-(pyren-1-ylmethyl) phthalazine* (**4**): A mixture of phthalazinone **3** (0.01 mol) and PCl_5_ (0.01 mol) was stirred together and boiled under reflux in POCl_3_ (40 mL, redistilled). After 2 h, the solution was clear and the POCl_3_ was distilled under reduced pressure. The residue was dissolved in CHCl_3_ and poured on ice-H_2_O. The aqueous layer was carefully adjusted to pH 8 with aq. NH_3_, the separated CHCl_3_ layer and the aqueous solution extracted with CHCl_3_ (50 mL). The CHCl_3_ extracts were combined dried over Na_2_SO_4_ and CHCl_3_ evaporated. The residue was then dissolved in benzene and the solution was purified first by passing through an alumina column and then through a charcoal column and then eluted with benzene. On evaporation of the eluates, a white crystalline solid of **4** was obtained.

*4-(Pyren-1-ylmethyl) phthalazine-1(2H)-selenone* (**5**): A mixture containing compound **4** (0.01 mol), selenium metal (0.012 mol) and sodium borohydride (0.03mol) was heated under reflux in 40 mL of ethanol for 8 h. The mixture was cooled and poured into ice-HCl. The solid formed was filtered off, washed with cold water, dried, and recrystallized from ethanol to give **5**.

*1,2-bis (4- (pyren-1-ylmethyl) phthalazin-1-yl) diselane* (**6**): Compound **6** precipitated during reflux of the above reaction mixture and was recrystallized from toluene as reddish-brown crystals.

*4-(Pyren-1-ylmethyl) phthalazine-1-thiol* (**7**)**:** To (0.05 mol) of phthalazinone **3** in 70 mL of boiling *p*-xylene, P_2_S_5_ (0.05 mol) was added in small portions, stirring. The mixture was heated on a metallic bath at 180 °C for 4 h and rapidly filtered, washing the residue with hot *p*-xylene. After cooling, the solid residue obtained was filtered off and crystallized from ethanol to give **7**.

*4-(Pyren-1-ylmethyl)-1-((trimethyl)silyloxy) phthalazine* (**8**): A solution of compound **3** (0.02 mol) in hexamethyldisilazane (60 mL) in the presence of a catalytic amount of (NH_4_)_2_SO_4_ was refluxed for 6 h. The excess hexamethyldisilazane was evaporated under reduced pressure, followed by co-evaporation with dry xylene. The solid residue was used directly for the next step.

*4-(pyren-1-ylmethyl)-1-(per-O-acetyl-d-glycosyloxy) phthalazine nucleosides* (**9a**,**b**) and (**12**): To a solution of compound **8** (0.02 mol) in ethylene chloride (50 mL) was added *α*-d-glucopyranose, *α*-d-galactopyranose pentaacetate, or *β*-d-ribopyranose tetracetate (0.02 mol) and stannic chloride (0.02 mol) was added. The reaction mixture was heated under reflux for 3 h with stirring. After cooling, it was neutralized with saturated aqueous solution of NaHCO3 and extract with CHCl_3_. The extracts were combined, washed with water, and dried in Na_2_SO_4_. The extracts on evaporation provided a white solid, which was purified by recrystallization of water to give **9a**, **b** and **12**, respectively.

*Free nucleosides* (**11a**,**b**) and (**13**): Et_3_N (1 mL) was added to a solution of acylated nucleosides **9a**,**b** and **12** (0.001 mol) in methanol (30 mL) and few drops of H_2_O. The mixture was stirred at 25 °C for 26 h, evaporated under reduced pressure, and the residue was co-evaporated with methanol until the excess Et_3_N was removed. The solid residue was crystallized from ethanol to give **11a**,**b** and **13**.

*4-(Pyren-1-ylmethyl)-1-(per-O-acetyl-d-glycosyl-1-thio) phthalazine* (**14**) and (**1**): To a solution of phthalazinethione **7** (0.01 mol) in Et_3_N (0.01 mol) and dry acetone (40 mL) was added acetobromo-*α*-d-glucose (0.01 mol). The mixture was stirred at 25 °C for 30 h and filtered. The filtrate was evaporated under reduced pressure and the solid residue was recrystallized from MeOH-H_2_O to give the acetylated *S-*nucleosides **14** and **17**.

*Free S-nucleosides* (**16**), (**18**) and (**20**): Dry gaseous NH_3_ was passed through a solution of each of the acetylated nucleosides **14, 17** and **19** (0.01 mol) in MeOH (15 mL) stirred at 5 °C for 3 h, then the reaction mixture was stirred at 25 °C for 24 h, then heated the reaction mixture for 1.5 h. at 110–115 °C. The resulting mixture was then concentrated at reduced pressure to give a solid residue, which was washed with boiling CHCl_3_ (150 mL). The crude nucleoside was dried and recrystallized with the appropriate solvent to give free *S* nucleosides **16**, **18**, and **20**.

*Ethyl 2-(1-oxo-4-(pyren-1-ylmethyl) phthalazin-2(1H)-yl) propanoate* (**21**): A mixture of phthalazinone **3** (0.01 mol), ethyl bromoacetate (0.04 mol) and K_2_CO_3_ (0.04 mol) in 50 mL of anhydrous acetone was heated under reflux for 24 h with stirring, cooled at 25 °C and then poured into H_2_O. The solid obtained was filtered off and crystallized from methanol to give **21.**

*2-(1-Oxo-4-(pyren-1-ylmethyl) phthalazin-2(1H)-yl) propanehydrazide* (**22**): A mixture of ester **21** (0.01 mol) and 80% hydrazine hydrate (0.03 mol) in 50 mL of absolute EtOH was heated under reflux for 3 h. After being cooled, the resultant solid was filtered off and crystallized from ethanol to give **22**.

*Acyclo C-nucleosides* (**23a**,**b**) and (**24**): A well-mixed mixture of compound 22 (0.01 mol) and d-glucono, d-galactono-1,5-lactone or d-glucoheptonic acid-1,4-lactone (0.01 mol) was heated in an oil bath for 2 h at 170–175 °C, whereby it melted and became pale yellow and then solidified. The solid residue was cooled and crystallized from ethanol-H_2_O to give **23a, b,** and **24**.

*Double-headed acyclo C-nucleoside analogous* (**25**): A solution of diethyl galactrate (0.01 mol) in pyridine (10 mL) was treated with the hydrazide derivative **22** (0.04 mol). The reaction mixture was heated under reflux for 3 h, allowing a solid product to precipitate, filter, washed with ethanol and recrystallized from EtOH-H_2_O to give **25**.

*Sugar hydrazones* (**26a**–**f**): To a well stirred mixture of the respective monosaccharide (d-(+)-glucose, d-(+)-galactose, mannose, l-(+) arabinose, d-(+)-xylose, d-(−)-ribose) [(0.01 mol) in H_2_O (2 mL)], a catalytic amount of glacial acetic acid (0.2 mL) in ethanol (30 mL) and the derivative hydrazide **22** (0.01 mol) were added. The mixture was heated under reflux for 2–4 h and the resulting solution was concentrated and left to cool. The precipitate formed was filtered, washed with water and ethanol, dried, and recrystallized from methanol to give **26a**–**f**, respectively.

*Acyclo C-nucleosides* (**27b**–**f**): A solution of hydrazones **26b**–**f** (0.01 mol) in acetic anhydride (20 mL), was heated under reflux for 2–3 h. The resulting solution was poured onto ice H_2_O and the product that separated was filtered, washed with a NaHCO_3_ solution followed by H_2_O, and then dried. The solid products were recrystallized from the appropriate solvent to give **27b**–**f**.

*Nucleoside analogues containing S and Se* (**28**–**32**): The selenolo derivatives of phthalazine **5** or **7** (0.01 mol) were heated under reflux with alkyl halide derivatives, *viz.* 4-bromobutylacetate, 2-acetoxyethoxymethyl bromide, 3-chloro propan-1-ol or 1,3-dichloro propan-2-ol (0.01 mol) for 3–4 h, in the presence of dry K_2_CO_3_/acetone. The reaction mixture was filtrated and the solvent evaporated under reduced pressure. The solid residue obtained was collected and recrystallized from the appropriate solvent to give **28**–**32**.

## 4. Characterization

The melting points were determined on the Electrothermal 9100 melting point apparatus from Electrothermal Engineering Ltd. (Rochford, England) and are not corrected. The IR spectra (KBr) were recorded on an FT-IR NEXCES spectrophotometer from CTech company (Shimadzu, Japan). The ^1^H NMR spectra were measured with a Jeol ECA 500 MHz (Tokyo, Japan) instrument in DMSO-*d*_6_ and chemical changes were recorded in *δ* ppm relative to TMS. Mass spectra (EI) were run at 70 eV with a Finnigan SSQ 7000 spectrometer. The purity of the compounds was checked on aluminum plates coated with silica gel (Merck). The elemental analysis for C, H, N and S was performed using a Costech model 4010 and the percentage values agreed with the proposed structures within ± 0.4% of the theoretical values.

### 4.1. Antimicrobial Activity

In vitro antimicrobial activities of some synthesized compounds were evaluated for their antibacterial activity against three bacterial strains, *Staphylococcus epidermidis* (MTCCB 1824), *Staphylococcus aureus* (MTCCB 737), and *Escherichia coli* (MTCCB 1652) and three fungal strains, namely, *Aspergillus niger*, *Aspergillus fumigatus*, and *Alternaria alternate* using the nutrient agar disc diffusion method [9,18,24] at 100 mg/mL concentration. Dimethylslphoxide, as a blank, exhibited no activity against any of the used organisms. Antimicrobial activity was evaluated by measuring the inhibition zone, after 20–24 h of incubation at 37 °C for bacterial strains and 3–4 days at 37 °C for fungal strains. Ketoconazole and tetracycline were used as reference drugs, respectively, at a concentration of 30 mg/mL. Preliminary screening data revealed that most compounds showed moderate to excellent antimicrobial activity when compared with ketoconazole and tetracycline as standard drugs.

### 4.2. Cytotoxicity Activity

The brine shrimp lethality bioassay [42,43] is a rapid and comprehensive bioassay for natural and synthetic bioactive compounds, indicating cytotoxicity and a wide range of pharmacological activities (e.g., antimicrobial, anticancer, antiviral, pesticide). In this method, Brine shrimp eggs were obtained from the New Aqua Laboratory in Naawan, Misamis Oriental, as a gift sample for research work. Artificial seawater was prepared by dissolving 38 g of sea salt in 1 L of distilled H_2_O to hatch the shrimp eggs and kept in a small tank. The eggs were then added to the divided tank. Constant oxygen supply was provided and a temperature of 37 °C was maintained for 48 h to hatch and mature the shrimp called nauplii (Larvae). The solutions of compounds **16**, **18**, **20**, **26a** and **33a** were prepared by dissolving 10 mg of each synthesized compound in 2 mL of DMSO. From this stock, a series of solutions 5, 10, 20, 40 and 80 mg/mL were transferred to 15 vials (three for each dilution were used for each test sample and LC50 is the mean of three values), and one vial was kept as a control having 2 mL of DMSO. Then, approximately 10 brine shrimp nauplii were applied to each of all experimental vials and control vials. The number of nauplii that died after 24 h was enumerated. The resulting data were transformed to Probit analysis 34 to determine LC50 values for the 5 compounds tested.

### 4.3. Computational Method

All molecular calculations were carried out in the gas phase using density functional theory (DFT) [42] combined with the hybrid functional B3LYP. The calculations were performed with a base set 6 to 31 + G(d) using the Gaussian 16 software package [43]. The fully optimized geometries were approved as global minima, since no imaginary frequencies were found as a result of the calculations in normal mode. Afterwards, the HOMO and LUMO level energy, LUMO–HOMO frontier orbital gap, and MESP map were computed for the optimized structures obtained.

## 5. Conclusions

In this study, 4-(pyren-1-ylmethyl) phthalazin-1(2*H*)-one (**3**) was used as critical synthons for the synthesis of a wide variety of novel, *S*- and acyclo *C*-nucleosides. In addition, it was also used for the synthesis of a new class of 4-substituted 1-(d-glycosyloxy) phthalazine nucleosides and *S*-containing nucleoside analogs with potentially interesting biological and medicinal properties. The antimicrobial activity of the synthesized compounds showed that some *S-*nucleosides, sugar hydrazine and *S*-nucleoside analogs of pyrenylmethylphthalazine has significant antimicrobial activity against different type of bacteria and fungi. The newly synthesized compounds are biocompatible and noncytotoxic materials. On the basis of HOMO and LUMO, the frontier orbital gap reflects the kinetic stability and chemical reactivity of the molecule. The molecular electrostatic potential investigates the sites of chemical reactivity for electrophilic and nucleophilic attacks. From antimicrobial measurements, cytotoxicity evaluation, chemical reactivity and kinematic stability of selected compound, we can conclude that some of *S-* and acyclo *C-*nucleosides could be used as new antimicrobial agent and drug delivery applications.

## Figures and Tables

**Table 1:** Antimicrobial activity of the synthesized compounds at 100 mg/mL concentration.

Compd. No.	Diameter of the Inhibition Zone ^a^ (mm)					
	Bacteria			Fungi		
	*Staphylococcus* *epidermidis* MTCCB 1824	*Staphylococcus* *aureus* MTCCB 737	*Escherichia coli* MTCCB 1652	*Aspergillus* *niger*	*Aspergillus* *fumigatus*	*Alternaria* *alternata*
16	23	22	19	18	21	19
18	20	23	19	19	17	18
20	26	27	26	14	15	14
26a	22	24	25	19	18	20
33a	27	28	29	15	17	16
Tetracycline ^b^	25	30	28	-	-	-
Ketoconazole ^b^	-	-	-	20	18	21

^a^ 12 mm or less: resistant or without inhibition, 13–17 mm: moderate inhibition, and 18 mm or more: maximum inhibition. ^b^ Concentration of reference drugs used was 30 mg/mL.

**Table 2:** Minimum inhibitory concentration (MIC, mg/mL) of the synthesized compounds **16**, **18**, **20**, **26a**, and **33a**.

The Selected Organisms	Minimum Inhibitory Concentration (MIC)					
	16	18	20	26a	33a	Standard ^a^
***Staphylococcus aureus* (MTCCB 737)**	50	50	25	50	50	6.25
***Escherichia coli* (MTCCB 1652)**	>100	>100	12.5	>100	25	12.5
***Aspergillus niger***	25	>100	25	25	50	6.25
***Alternaria alternata***	25	50	50	25	50	6.25

^a^ Tetracycline and ketoconazole were used as reference drugs against bacterial and fungal strains, respectively.

**Table 3:** Cytotoxicity activity of the synthesized compounds **16**, **18**, **20**, **26a** and **33a**.

Samples	95% Confidence Limit ppm			Regression Equation	*X^2^* (df)
	LC_50_	Lower	Upper		
**16**	1.39	0.69	2.82	y = 3.54 + 1.29*x*	0.41 (2)
**18**	2.31	1.30	4.10	y = 4.36 + 1.78*x*	0.32 (2)
**20**	3.54	2.08	6.02	y = 3.98 + 1.85*x*	3.38 (2)
**26a**	0.58	0.19	1.77	y = 4.08 + 1.22*x*	0.20 (2)
**33a**	6.49	4.16	10.15	y = 3.16 + 2.28*x*	0.36 (2)
**Bleomycin ^a^**	0.41	0.27	0.62	y = 3.16 + 2.98*x*	0.62 (2)
**Gallic acid ^a^**	4.53	3.33	6.15	y = 3.93 + 1.62*x*	1.25 (2)

^a^ Bleomycin and gallic acid were used as standard drugs in cytotoxicity activity.

**Table 4:** HOMO energy, LUMO energy and LUMO–HOMO energy gap of compounds calculated using the DFT (B3LYP)/6–31 + G(d) method.

Compound Number	HOMO (eV)	LUMO (eV)	Gap Energy HOMO–LUMO (eV)
**1**	−7.55109	−1.77795	5.77
**16a**	−5.32764	−2.02581	3.30
**18a**	−5.3001	−1.65888	3.64
**20a**	−5.30172	−1.64781	3.65
**26a**	−5.26257	−1.47582	3.78
**29a**	−5.28363	−1.69722	3.58
**33a**	−5.45913	−1.92429	3.53

**Table 5:** Global descriptors of chemical reactivity of structures **1**, **16a**, **18a**, **20a**, **26a**, **29a** and **33a**.

Compound Number	*I* (eV)	*A* (eV)	*µ* (eV)	*ꭓ* (eV)	*η* (eV)	*σ* (eV)	ω
**1**	7.55	1.77	−4.66	4.66	2.89	0.38	3.76
**16a**	5.32	2.02	−3.67	3.67	1.65	0.31	4.09
**18a**	5.30	1.65	−3.47	3.47	1.82	0.34	3.31
**20a**	5.30	1.64	−3.47	3.47	1.83	0.34	3.29
**26a**	5.26	1.47	−3.36	3.36	1.89	0.36	2.99
**29a**	5.28	1.69	−3.49	3.49	1.79	0.34	3.39
**33a**	5.45	1.92	−3.69	3.69	1.76	0.32	3.86

## Data Availability

Most of the data presented in this study are available in the supplementary material. Additional data presented in this study are available on request from the corresponding author.

## Supplementary Materials

The following supporting information can be downloaded at: https://www.mdpi.com/article/10.3390/molecules28010317/s1. All data of novel synthesized compounds were fully characterized using different techniques like ^1^H-NMR, FT-IR, mass spectroscopy, elemental analysis and is presented in the supporting information. Figure S1: 3D plots of HOMO and LUMO molecular orbitals of some compounds calculated using the DFT(B3LYP)/ 6–31+G(d) method; Figure S2: Illustrative figures of molecular electrostatic potentials (MESP) mapped on the electron density surface calculated by the B3LYP/6–31 + G(d) method for some selected compounds. Symbols are: O (red), C (gray), H (white), and N (blue); Table S1: HOMO energy, LUMO energy, and LUMO-HOMO energy gap of compounds calculated using the DFT(B3LYP)/ 6–31 + G(d) method; Table S2: Global descriptors of chemical reactivity of the structures of some studied compounds using the DFT(B3LYP)/ 6–31 + G(d) method.
