# Supplementary material for: Synthesis of Some Mono- and Disaccharide-Grafting Phthalazine Derivatives and Some New Se-Nucleoside Analogues: Antibacterial Properties, Quantum Chemical Calculations, and Cytotoxicity
Source: Molecules. 2022 Dec 30;28(1):317. doi: 10.3390/molecules28010317 (PMC9822378; doi:10.3390/molecules28010317)
Supplement: Supplementary file 1 [file molecules-28-00317-s001.zip › molecules-2099936-supplementary.pdf]

Supplementary Materials

Synthesis of Some Mono- and Disaccharide-Grafting Phthalazine Derivatives and Some New *Se*-Nucleoside Analogues: Antibacterial Properties, Quantum Chemical Calculations, and Cytotoxicity

I. E. El-Shamy [1], E. Hleli [2], A. A. Alsheikh [3], M. A. Yawer [4], M. A. El-Hashash [5], J. Dybal [2] and A. M. Abdel-Mohsen [2,*]

[1] Chemistry Department, Faculty of Science, Fayoum University, Fayoum 63514, Egypt
[2] Institute of Macromolecular Chemistry, Czech Academy of Sciences, Heyrovského nám. 2, 162 06 Prague, Czech Republic
[3] Department of Chemical engineering, Faculty of Chemical and Petroleum Engineering, Albaath University, Homs 12574, Syria
[4] Department of Chemistry, Division of Science and Technology, University of Education Lahore, Lahore 32200, Pakistan
[5] Chemistry Department, Faculty of Science, Ain Shams University, Cairo 11566, Egypt
* Correspondence: abdellatif@imc.cas.cz

Results

Synthesis of 3-Hydroxy-2-(pyren-1-yl)-1*H*-inden-1-one (2): Yield: 69%, m.p.: 142–143 °C; ^{1}H-NMR (DMSO-d$_6$, δ): 8.04 (t, 1H, $^3J_{HH}$ = 7.5 Hz, *Pyr-H*), 8.10-8.18 (m, 3H, *Pyr-H*), 8.21-8.24 (m, 3H, *Pyr-H*), 8.30 (d, 1H, $^3J_{HH}$ = 8.5 Hz, *Pyr-H*), 8.46-8.49 (m, 1H, *Pyr-H*), 7.34-7.68 (m, 4H, *Indenone-H*), 13.20 (s, 1H, D_2O exchangeable OH); FT-IR (KBr, v,cm^{-1}): 3462(OH), 1689(C=O) cm^{-1}; MS (m/z): 346 (M$^+$, 17). Anal. calcd. for $C_{25}H_{14}O_2$: C, 86.69; H, 4.07; found C, 86.71; H, 4.02.

Synthesis of 4-(Pyren-1-ylmethyl)phthalazin-1(2*H*)-one (3): Yield: 66%, m.p.: 256–257 °C; ^{1}H-NMR (DMSO-d$_6$, δ): 4.69 (s, 2H, CH_2), 7.55 (t, 1H, $J_{6,7}$ = 7.0 Hz, H-7, *Phthalazi-H*), 7.68 (d, 1H, $J_{7,8}$ = 7.6 Hz, H-8, *Phthalaz-H*), 7.81 (t, 1H, H-6, *Phthalaz-H*), 7.90 (d, 1H, $J_{5,6}$ = 8.1 Hz, H-5, *Phthalaz-H*), 8.01 (dd, 1H, $^3J_{HH}$ = 7.8 and 7.3 Hz, *Pyr-H*), 8.08-8.13 (m, 2H, *Pyr-H*), 8.19 (d, 1H, $^3J_{HH}$ = 9.8 Hz, *Pyr-H*), 8.24 (d, 2H, $^3J_{HH}$ = 7.9 Hz, *Pyr-H*), 8.29 (d, 1H, $^3J_{HH}$ = 8.5 Hz, *Pyr-H*), 8.40-8.48 (m, 2H, *Pyr-H*), 11.33 (s, 1H, D_2O exchangeable NH); ^{13}C NMR: δ 37.1(CH_2), 120.2, 121.1, 122.6, 124.3, 124.9, 125.0, 125.2, 125.5, 125.9, 126.4, 127.7, 127.9, 128.4, 129.0, 129.5, 129.8, 130.1, 130.5, 130.9, 131.5, 131.9, 132.0, 140.3 (Ar-C), 166.2 (CO); FT-IR (KBr, v,cm^{-1}): 3210 (NH), 1668(C=O) cm^{-1}; MS (m/z): 360 (M$^+$, 22). Anal.calcd. for $C_{25}H_{16}N_2O$: C, 83.31; H, 4.47; N, 7.77; found C, 83.33; H, 4.40; N, 7.79.

Synthesis of 1-Chloro-4-(pyren-1-ylmethyl)phthalazine (4): Yield: 60%, m.p.: 133–134 °C; ^{1}H-NMR (DMSO-d$_6$, δ): 4.75 (s, 2H, CH_2), 7.59 (m, 2 H, H-7,8, *Phthalaz-H*), 7.72 (t, 1 H, $J_{6,7}$ = 8.4, $J_{5,6}$ = 8.0 Hz, H-6, *Phthalaz-H*), 7.84 (d, 1 H, $J_{5,6}$ = 7.9 Hz, H-5, *Phthalaz-H*), 8.03 (t, 1H, $^3J_{HH}$ = 7.5 Hz, *Pyr-H*), 8.07-8.11 (m, 2H, *Pyr-H*), 8.16 (d, 1H $^3J_{HH}$ = 9.0 Hz, *Pyr-H*), 8.22 (d, 2H, $^3J_{HH}$ = 7.8 Hz, *Pyr-H*), 8.24 (d, 1H, $^3J_{HH}$ = 8.0 Hz, *Pyr-H*), 8.40 (d, 1H, $^3J_{HH}$ = 7.5 Hz, *Pyr-H*), 8.49 (d, 1H, $^3J_{HH}$ = 8.0 Hz, *Pyr-H*); ^{13}C NMR: δ 38.4(CH_2); 120.8, 121.5, 122.4, 124.1, 124.8, 125.0, 125.1, 125.4, 126.0, 126.9, 127.9, 128.1, 128.7, 129.0, 129.2, 129.7, 130.0, 130.4, 130.8, 131.4, 132.0, 132.4, 141.4 (Ar-C), 1501.2 (C-Cl); FT-IR (KBr, v,cm^{-1}): 1608, 1503 (aromatic C-H) cm^{-1}; MS (m/z): 378 (M$^+$, 9).Anal.calcd. for $C_{25}H_{15}ClN_2$: C, 79.26; H, 3.99; N, 7.39; found C, 79.29; H, 3.96; N, 7.30.

Synthesis of 4-(Pyren-1-ylmethyl)phthalazine-1(2*H*)-selenone (5): Yield: 85%, m.p.: 201–202 °C; ^{1}H-NMR (DMSO-d$_6$, δ): 4.72 (s, 2H, CH_2), 7.48 (t, 1 H, J = 7.0 Hz, H-7, *Phthalaz-H*), 7.59 (d, 1 H, $J_{7,8}$ = 7.5 Hz, H-8, *Phthalaz-H*), 7.68 (t, 1 H, H-6, *Phthalaz-H*), 7.84 (d, 1 H, $J_{5,6}$ = 8.4, H-5, *Phthalaz-H*), 8.07 (d, 1H, $^3J_{HH}$ = 8.7 Hz, *Pyr-H*), 8.02-8.09 (m, 2H, *Pyr-H*), 8.20 (d, 1H, $^3J_{HH}$ = 8.0 Hz, *Pyr-H*); 8.25 (d, 1H, $^3J_{HH}$ = 7.8 Hz, *Pyr-H*), 8.28-8.30 (m, 2H, *Pyr-H*), 8.36 (dd, 1H, $^3J_{HH}$ = 8.4 and $^4J_{HH}$ = 2.6 Hz, *Pyr-H*), 8.50 (d, 1H $^3J_{HH}$ = 9.0 Hz, *Pyr-H*), 9.10 (s, 1H, D_2O exchangeable NH); ^{13}C NMR: δ 38.0(CH_2), 121.2, 121.7, 123.4, 124.0, 124.6, 124.8, 125.0, 125.2, 126.1, 127.0, 127.7, 128.0, 128.6, 128.8, 129.1, 129.6, 130.0, 130.6, 130.9, 131.3, 131.8, 132.0, 140.1 (Ar-C), 149.5(C=Se); FT-IR (KBr, v,cm^{-1}): 3222 (NH) cm^{-1}; MS (m/z): 423 (M$^+$, 11).Anal.calcd. for $C_{25}H_{16}N_2Se$: C, 70.92; H, 3.81; N, 6.62; found C, 70.98; H, 3.79; N, 6.60.

Synthesis of 4-(Pyren-1-ylmethyl)phthalazine-1-thiol (7): Yield: 81%, m.p.: 192–193 °C; ^{1}H-NMR (DMSO-d$_6$, δ): 4.70 (s, 2H, CH$_2$), 6.11 (s, 1H, D$_2$O exchangeable NH), 7.51 (t, 1 H, $J_{6,7}$ = 7.0 Hz, H-7, *Phthalaz-H*), 7.60 (d, 1 H, $J_{7,8}$ = 7.0 Hz, H-8, *Phthalaz-H*), 7.77 (t, 1H, H-6, *Phthalaz-H*), 7.83 (d, 1 H, $J_{5,6}$ = 8.0 Hz, H-5, *Phthalaz-H*), 8.01 (t, 1H, $^3J_{HH}$ = 7.6 Hz, *Pyr-H*), 8.05-8.11 (m, 2H, *Pyr-H*), 8.17 (d, 1H, $^3J_{HH}$ = 8.1 Hz, *Pyr-H*), 8.21 (d, 2H, $^3J_{HH}$ = 7.6 Hz, *Pyr-H*), 8.27 (d, 2H, $^3J_{HH}$ = 8.5 Hz, *Pyr-H*), 8.38 (dd, 1H, $^3J_{HH}$ = 8.5 and $^4J_{HH}$ = 2.4 Hz, *Pyr-H*), 8.45-8.48 (m, 1H, *Pyr-H*); ^{13}C NMR: δ 37.7(CH$_2$), 121.0, 121.9, 123.0, 124.2, 124.8, 125.0, 125.3, 125.8, 126.4, 127.2, 127.5, 128.0, 128.3, 128.9, 129.4, 129.9, 130.9, 131.5, 131.9, 132.0, 132.8, 133.1, 146.6 (Ar-C), 173.5(C=S); FT-IR (KBr, v,cm^{-1}): 2410(C=S), 1323 cm^{-1}; MS (m/z): 376 (M$^+$, 18). Anal.calcd. for $C_{25}H_{16}N_2S$: C, 79.76; H, 4.28; N, 7.44; S, 8.52; found C, 79.80; H, 4.30; N, 7.44; S, 8.49.

Synthesis of 4-(Pyren-1-ylmethyl)-1-(2,3,4,6-tetra-*O*-acetyl-*β*-D glucopyranosyloxy)phthalazine (9a): Yield: 79%, m.p.: 93–94 °C; ^{1}H-NMR (DMSO-d$_6$, δ): 1.59, 1.96, 2.04, 2.26 (4s, 12 H, 4 OCOCH$_3$), 4.11 (m, 1 H, H-5′), 4.31 (dd, 1 H, $J_{5',6'}$ 2.1, $J_{6',6'}$ 11.9 Hz, H-6′′), 4.46 (dd, 1 H, $J_{5',6'}$ 4.8, $J_{6',6''}$12.1 Hz, H-6′), 4.77 (s, 2H, CH$_2$), 5.33 (t, 1 H, $J_{3',4'}$ 10.1, $J_{4',5'}$ 10.2 Hz, H-4′), 5.47 (t, 1 H, $J_{2',3'}$ 9.5 Hz, H-2′), 5.54 (t, 1 H, $J_{3'4'}$ 10.2 Hz, H-3′), 5.60 (d, 1 H, $J_{1',2'}$ 9.0 Hz, H-1′), 7.60 (t, 1 H, $J_{6,7}$ = 7.5 Hz, H-7, *Phthalaz-H*), 7.68 (d, 1 H, J = 7.3 Hz, H-8, *Phthalaz-H*), 7.75 (t, 1 H, J = 8.0 Hz, H-6, *Phthalaz-H*), 7.89 (d, 1 H, $J_{5,6}$ = 8.3 Hz, H-5, *Phthalaz-H*), 8.04 (t, 1H, $^3J_{HH}$ = 7.4 Hz, *Pyr-H*); 8.05- 8.10 (m, 2H, *Pyr-H*); 8.15 (d, 1H, $^3J_{HH}$ = 9.0 Hz, *Pyr-H*), 8.20-8.25 (m, 2H, *Pyr-H*), 8.28 (d, 2H, $^3J_{HH}$ = 8.4 Hz, *Pyr-H*), 8.45-8.48 (m, 1H *Pyr-H*); ^{13}C NMR: δ 19.9, 20.2, 20.4, 20.9 (4 CH$_3$), 37.0(CH$_2$), 62.1 (C-6′), 68.2 (C-4′), 70.9 (C-3′), 71.4 (C-2′), 74.9 (C-5′), 93.9 (C-1′), 121.4, 121.8, 122.7, 124.0, 124.5, 125.1, 125.4, 125.7, 126.0, 126.8, 127.1, 128.2, 128.4, 128.8, 129.0, 129.4, 130.4, 131.0, 131.3, 131.7, 132.0, 133.4, 139.6 (Ar-C), 169.0, 169.5, 169.9, 170.1 (4C=O), 172.1 (N=C-O), FT-IR (KBr, v,cm^{-1}): 1738 (C=O ester) cm^{-1}. MS (m/z): 690 (M$^+$, 14). Anal.calcd. for $C_{39}H_{34}N_2O_{10}$: C, 67.82; H, 4.96; N, 4.06; found C, 67.86; H, 4.90; N, 4.10.

Synthesis of 4-(Pyren-1-ylmethyl)-1-(2,3,4,6-tetra-*O*-acetyl-*β*-D-galactopyranosyloxy)phthalazine (9b): Yield: 82%, m.p.: 111–112 °C; ^{1}H-NMR (DMSO-d$_6$, δ): 2.10, 2.16. 2.18, 2.30 (3 s, 12 H, 4 OCOCH$_3$), 4.32 (dd, 1 H, $J_{5',6'}$ 7.3, $J_{6',6''}$ 11.1 Hz, H-6′), 4.35 (dd, 1 H, $J_{5',6''}$, 7.3, $J_{6',6''}$ 12.1 Hz, H-6′′), 4.44 (m, 1 H, H-5′), 4.70 (s, 2H, CH$_2$), 5.30 (dd, 1 H, $J_{2',3'}$ 9.9, $J_{3',4'}$ 4.0 Hz, H-3′), 5.50 (dd, 1 H, $J_{2',3'}$ 11.5, $J_{1',2'}$ 9.1 Hz, H-2), 5.63 (d, 1 H, $J_{1,2}$ 8.8 Hz, H-1′), 5.71 (d, 1 H, $J_{4',5'}$ 3.0 Hz, H-4′), 7.52 (t, 1 H, $J_{6,7}$ = 7.0 Hz, H-7, *Phthalaz-H*), 7.61 (d, 1 H, $J_{7,8}$ = 7.9 Hz, H-8, *Phthalaz-H*), 7.72 (t, 1 H, H-6, *Phthalaz-H*), 7.81 (d, 1 H, $J_{5,6}$ = 8.5 Hz, H-5, *Phthalaz-H*), 8.05-8.07 (m, 2H, *Pyr-H*); 8.10 (d, 1H, $^3J_{HH}$ = 8.0 Hz, *Pyr-H*); 8.19 (d, 1H, $^3J_{HH}$ = 7.3 Hz, *Pyr-H*); 8.22-8.25 (m, 3H, *Pyr-H*); 8.30 (dd, 1H, $^3J_{HH}$ = 8.3 and $^4J_{HH}$ = 2.7 Hz, *Pyr-H*); 8.48 (d, 1H, $^3J_{HH}$ = 9.0 Hz, *Pyr-H*); ^{13}C NMR: δ 19.5, 20.1, 20.3, 20.8 (4 CH3), 37.5(CH$_2$), 61.7 (C-6′), 67.1 (C-4′), 69.0 (C-2′), 69.5 (C-3′), 73.9 (C-5′), 94.1 (C-1′), 121.0, 121.3, 122.1, 123.2, 124.1, 124.9, 125.1, 125.5, 126.2, 126.7, 127.0, 127.7, 128.2, 128.9, 129.1, 129.4, 130.6, 131.1, 131.5, 131.9, 132.2, 133.1, 138.2 (Ar-C), 169.0, 169.8, 169.9, 170.6, (O-C=O), 171.2 (N=C-

O). FT-IR (KBr, v,cm^{-1}): 1735 (C=O ester) cm^{-1}. MS (m/z): 690 (M$^+$, 12). Anal.calcd. for $C_{39}H_{34}N_2O_{10}$: C, 67.82; H, 4.96; N, 4.06; found C, 67.81; H, 4.92; N, 4.13.

Synthesis of 4-(Pyren-1-ylmethyl)-1-(2,3,5-tri-*O*-acetyl-*α*-D-ribofuranosyloxy)phthalazine (12): Yield: 77%, m.p.: 88–89 °C; ^{1}H-NMR (DMSO-d$_6$, δ): 2.00, 2.06, 2.18 (3 s, 9H, 3 $OCOCH_3$), 4.44, 4.49 (2 d, 2 H, $J_{4',5''}$ 5.0, $J_{4',5'}$ 2.8, $J_{5',5''}$ 9.1 Hz, H-5′,5′′), 4.60 (m, 1 H, H-4′), 4.78 (s, 2H, CH_2), 5.50 (dd, 1 H, $J_{2',3'}$ 5.8, $J_{3',4'}$ 7.9 Hz, H-3′), 5.79 (dd, 1 H, $J_{1',2'}$ 2.1, $J_{2',3'}$ 6.1 Hz, H-2′), 5.88 (d, 1 H, H-1′), 7.55 (m, 2 H, H-7,8, *Phthalaz-H*), 7.60 (t, 1 H, $J_{6,7}$ = 7.9, $J_{5,6}$ = 7.9 Hz, H-6, *Phthalaz-H*), 7.81 (d, 1 H, $J_{5,6}$ = 7.8 Hz, H-5, *Phthalaz-H*), 8.03 (t, 1H, $^3J_{HH}$ = 7.5 Hz, *Pyr-H*), 8.06-8.09 (m, 2H, *Pyr-H*), 8.18 (d, 1H, $^3J_{HH}$ = 9.0 Hz, *Pyr-H*), 8.24 (d, 2H, $^3J_{HH}$ = 9.0 Hz, *Pyr-H*), 8.29 (d, 1H, $^3J_{HH}$ = 7.8 Hz, *Pyr-H*), 8.39 (d, 1H, $^3J_{HH}$ = 9.0 Hz, *Pyr-H*), 8.40-8.44 (m, 1H, *Pyr-H*); ^{13}C NMR: δ_C 20.1, 20.5, 20.9 (3 CH_3), 37.3(CH_2), 61.9 (C-5′), 69.0 (C-3′), 74.4 (C-2′), 80.8 (C-4′), 99.8 (C-1′), 121.1, 121.4, 122.0, 122.5, 124.0, 124.5, 125.2, 125.6, 126.1, 126.6, 127.2, 127.7, 128.1, 128.8, 129.2, 129.4, 130.1, 130.5, 131.1, 131.6, 132.0, 132.8, 139.4 (Ar-C). 169.1 (N=C-O), 169.7, 169.9, 172.4 (3O-C=O); FT-IR (KBr, v, cm^{-1}): 1733 (C=O ester) cm^{-1}. MS (m/z): 618 (M$^+$, 13). Anal.calcd. for $C_{36}H_{30}N_2O_8$: C, 69.89; H, 4.89; N, 4.53; found C, 69.92; H, 4.84; N, 4.50.

Synthesis of free nucleosides (11a,b) *and* (13)

Synthesis of 4-(Pyren-1-ylmethyl)-1-(*β*-D-glucopyranosyloxy) phthalazine (11a): Yield: 68%, m.p.: 144–145 °C; ^{1}H-NMR (DMSO-d$_6$, δ): 3.30 (m, 6H, H-6′, H-6′′, H-5′, H-4′, H-3′ and H-2′), 4.21 (t, 1H, J = 3.66 Hz, OH-6′, D_2O exchangeable), 4.54 (d, 1H, J = 5.03 Hz, OH-4′, D_2O exchangeable), 4.74 (s, 2H, CH_2), 5.20 (d, 1H, J = 4.44 Hz, OH-3′, D_2O exchangeable), 5.49 (d, 1H, J = 5.08 Hz, OH-2′, D_2O exchangeable), 6.01 (d, 1H, $J_{1',2'}$ = 8.54 Hz, H-1′), 7.49 (t, 1 H, $J_{6,7}$ = 7.0 Hz, H-7, *Phthalaz-H*), 7.62 (d, 1 H, $J_{7,8}$ = 7.1 Hz, H-8, *Phthalaz-H*), 7.70 (t, 1 H, H-6, *Phthalaz-H*), 7.85 (d, 1 H, $J_{5,6}$ = 7.8 Hz,H-5, *Phthalaz-H*), 7.98-8.02 (m, 1H, *Pyr-H*); 8.10 (t, 1H, $^3J_{HH}$ = 7.5 Hz, *Pyr-H*); 8.22 (d, 1H, $^3J_{HH}$ = 9.0 Hz, *Pyr-H*); 8.25 (d, 1H, $^3J_{HH}$ = 9.0 Hz, *Pyr-H*); 8.29 (d, 1H, $^3J_{HH}$ = 8.8 Hz, *Pyr-H*); 8.36 (d, 1H, $^3J_{HH}$ = 7.2 Hz, *Pyr-H*); 8.39 (d, 1H, $^3J_{HH}$ = 7.5 Hz, *Pyr-H*); 8.44 (d, 1H, $^3J_{HH}$ = 8.2 Hz, *Pyr-H*); 8.46-8.51 (m, 1H, *Pyr-H*); FT-IR (KBr, v,cm^{-1}): 3477(broad, OH) cm^{-1}; MS (m/z): 522 (M$^+$, 5). Anal.calcd. for $C_{31}H_{26}N_2O_6$: C, 71.25; H, 5.02; N, 5.36; found C, 71.29; H, 5.10; N, 5.32.

Synthesis of 4-(Pyren-1-ylmethyl)-1-(*β*-D-galactopyranosyloxy) phthalazine (11b): Yield: 77%, m.p.: 131–132 °C; ^{1}H-NMR (DMSO-d$_6$, δ): 3.50 (m, 3H, H-3′, H-6′, H-6′′), 3.61 (m, 3H, H-2′, H-4′, H-5′), 4.60 (m, 2H, OH-4′, OH-6′, D_2O exchangeable), 4.76 (s, 2H, CH_2), 5.05 (d, 1H, J = 5.22 Hz, OH-3′, D_2O exchangeable), 5.30 (d, 1H, J = 4.91 Hz, OH-2′, D_2O exchangeable), 5.99 (d, 1H, $J_{1',2'}$ = 9.02 Hz, H-1′), 7.50 (t, 1 H, $J_{6,7}$ = 7.5 Hz, H-7, *Phthalaz-H*), 7.61 (d, 1 H, J = 7.3 Hz, H-8, *Phthalaz-H*), 7.69 (t, 1 H, J = 8.5 Hz, H-6, *Phthalaz-H*), 7.89 (d, 1 H, $J_{5,6}$ = 7.8 Hz, H-5, *Phthalaz-H*), 8.08 (t, 1H $^3J_{HH}$ = 7.5 Hz, *Pyr-H*), 8.11 (d, 1H, $^3J_{HH}$ = 8.9 Hz, *Pyr-H*), 8.16 (d, 1H, $^3J_{HH}$ = 8.9 Hz, *Pyr-H*), 8.20 (d, 1H, $^3J_{HH}$ = 8.1 Hz, *Pyr-H*), 8.24-8.29 (m, 3H, *Pyr-H*); 8.46 (d, 1H, $^3J_{HH}$ = 8.0 Hz, *Pyr-H*), 8.49 (d, 1H, $^3J_{HH}$ = 9.5 Hz, *Pyr-H*); 13C NMR: δ 37.1(CH_2), 61.2

(C-6′), 67.1 (C-4′), 68.9 (C-2′), 71.4 (C-3′), 78.9 (C-5′), 99.0 (C-1′), 121.0, 121.7, 122.2, 122.5, 123.1, 124.2, 125.1, 125.5, 126.2, 126.6, 127.4, 127.8, 128.3, 128.8, 129.1, 129.3, 130.0, 130.4, 131.1, 131.7, 132.1, 132.7, 138.6 (Ar-C), 169.2 (N=C-O). FT-IR (KBr, *v*,cm^{-1}): 3493(broad, OH) cm^{-1}; MS (m/z): 522 (M$^+$, 4). Anal.calcd. for $C_{31}H_{26}N_2O_6$: C, 71.25; H, 5.02; N, 5.36; found C, 71.30; H, 5.11; N, 5.34.

Synthesis of 4-(Pyren-1-ylmethyl)-1-(*α*-D-ribofuranosyloxy)phthalazine (13): Yield: 66%, m.p.: 122–123 °C; ^{1}H-NMR (DMSO-d$_6$, *δ*): 3.15 (m, 2H, H-5′, H-5′′), 3.29 (m, 1H, H-4′), 3.50-3.55 (2H, H-3′ , H-2′), 4.30 (t, 1H, *J* = 6.0 Hz, OH-5′, D_2O exchangeable), 4.58 (d, 1H, *J* = 5.01 Hz, OH-3′, D_2O exchangeable), 4.69 (d, 1H, *J* = 4.9 Hz, OH-2′, D_2O exchangeable), 4.78 (s, 2H, CH_2), 6.02 (d, 1H, $J_{1',2'}$ = 4.03 Hz, H-1′), 7.49 (t, 1 H, $J_{6,7}$ = 7.0 Hz, H-7, *Phthalaz-H*), 7.58 (d, 1 H, *J* = 6.9 Hz, H-8, *Phthalaz-H*), 7.73 (t, 1 H, *J* = 8.1 Hz, H-6, *Phthalaz-H*), 7.82 (d, 1 H, $J_{5,6}$ = 7.8 Hz, H-5, *Phthalaz-H*), 8.03 (t, 1H, $^3J_{HH}$ = 7.6 Hz, *Pyr-H*), 8.07 (d, 1H, $^3J_{HH}$ = 8.9 Hz, *Pyr-H*), 8.10 (d, 1H, $^3J_{HH}$ = 8.9 Hz, *Pyr-H*), 8.15 (d, 1H, $^3J_{HH}$ = 9.3 Hz, *Pyr-H*), 8.22- 8.21 (m, 2H, *Pyr-H*); 8.25 (d, 1H, $^3J_{HH}$ = 8.0 Hz, *Pyr-H*), 8.32 (d, 1H, $^3J_{HH}$ = 9.3 Hz, *Pyr-H*), 8.40 (dd, 1H, $^3J_{HH}$ = 8.0 Hz, $^4J_{HH}$ = 2.2 Hz, *Pyr-H*); 13C NMR: δ 37.1(CH_2), 61.0 (C-5′), 68.8 (C-3′), 77.0 (C-2′), 87.1 (C-4′), 101.7 (C-1′), 120.8, 121.4, 122.0, 122.3, 123.0, 124.4, 125.2, 125.6, 126.1, 126.8, 127.3, 127.8, 128.1, 128.7, 129.2, 129.4, 130.1, 130.5, 131.2, 131.7, 132.3, 132.0, 139.1 (Ar-C), 168.8 (N=C-O). FT-IR (KBr, *v*,cm^{-1}): 3477(broad, OH) cm^{-1}; MS (m/z): 492 (M$^+$, 7). Anal.calcd. for $C_{30}H_{24}N_2O_5$: C, 73.16; H, 4.91; N, 5.69; found C, 73.13; H, 4.90; N, 5.71.

Synthesis of 4-(Pyren-1-ylmethyl)-1-(2,3,4,6-tetra-*O*-acetyl-*β*-D-glucopyranosyl-1-thio)phthalazine (14): Yield: 60%, m.p.: 69–70°C; ^{1}H NMR (DMSO-d$_6$, *δ*): 1.80, 1.89,1.90, 1.96 (4s, 12H, 4OCOCH$_3$), 3.92(m, 1H, H-5′), 4.18 (dd, 1H, $J_{5',6'}$= 1.88, $J_{6',6''}$= 11.89 Hz, H-6′′), 4.43 (dd, 1H, $J_{5',6'}$ = 6.12, $J_{6',6''}$= 11.66 Hz, H-6′), 4.74 (s, 2H, CH_2), 5.13 (t, 1H, $J_{3',4'}$= 10.80, $J_{4',5'}$ = 10.01 Hz, H-4′), 5.22 (t, 1H, $J_{2',3'}$= 9.30 Hz, H-2′), 5.39(t, 1H, $J_{3',4'}$= 10.22 Hz, H-3′), 5.59 (d, 1H, $J_{1',2'}$= 11.02 Hz, H-1′), 7.57 (t, 1 H, *J* = 7.0 Hz, H-7, *Phthalaz-H*), 7.63 (d, 1 H, $J_{7,8}$ = 7.9 Hz, H-8, *Phthalaz-H*), 7.69 (t, 1 H, H-6, *Phthalaz-H*), 7.90 (d, 1 H, $J_{5,6}$ = 8.4, H-5, *Phthalaz-H*), 8.01-8.04 (m, 2H, *Pyr-H*), 8.09 (d, 1H, $^3J_{HH}$ = 8.9 Hz, *Pyr-H*), 8.15 (d, 1H, $^3J_{HH}$ = 8.0 Hz, *Pyr-H*), 8.20 (d, 1H, $^3J_{HH}$ = 7.5 Hz, *Pyr-H*), 8.21-8.25 (m, 2H, *Pyr-H*), 8.33 (dd, 1H, $^3J_{HH}$ = 8.0 and $^4J_{HH}$ = 2.5 Hz, *Pyr-H*), 8.48 (d, 1H, $^3J_{HH}$ = 9.5 Hz, *Pyr-H*); ^{13}C NMR: *δ* 20.1, 20.3, 20.7, 21.2 (4 CH_3), 37.2(CH_2), 62.0 (C-6′), 68.1 (C-4′), 71.0 (C-3′), 71.2 (C-2′), 74.8 (C-5′), 90.5 (C-1′), 121.4, 121.8, 122.7, 124.0, 124.5, 125.1, 125.4, 125.7, 126.0, 126.8, 127.1, 128.2, 128.4, 128.8, 129.0, 129.4, 130.4, 131.0, 131.3, 131.7, 132.0, 133.4, 139.6 (Ar-C), 162.1 (N=C-S), 168.2, 169.1, 169.4, 170.3 (4O-C=O); FT-IR (KBr, *v*, cm^{-1}): 1735 (C=O ester) cm^{-1}. MS (m/z): 706 (M$^+$, 17). Anal.calcd. for $C_{39}H_{34}N_2O_9S$: C, 66.28; H, 4.85; N, 3.96; S, 4.54; found C, 66.30; H, 4.85; N, 3.92; S, 4.50.

Synthesis of 4-(Pyren-1-ylmethyl)-1-(2,3,5-tri-*O*-acetyl-*β*-D-xylofuranosyl-1-thio)phthalazine (17): Yield: 55%, m.p.:81–82°C; ^{1}H NMR (DMSO-d$_6$, *δ*): 1.99, 2.19, 2.33 (3s, 9H, 3 OCOCH$_3$), 3.80, (dd, 1H, $J_{4',5'}$ = 7.5, $J_{5',5''}$ = 11.9 Hz, H-5′), 4.08 (dd, 1H, $J_{4',5''}$= 4.11, $J_{5',5''}$ = 11.9 Hz, H-5′′), 4.79 (s, 2H, CH_2), 5.22 (m, 1H, H-4′), 5.35 (t, 1H, $J_{1',2'}$ = 4.3, $J_{2',3'}$ = 10.2 Hz, H-2′), 5.59 (t, 1H, $J_{2',3'}$ = 10.2, $J_{3',4'}$ = 10.2 Hz, H-3′), 6.08 (d, 1H,

$J_{1',2'}$ = 4.55 Hz, H-1′), 7.49 (m, 2 H, H-7,8, *Phthalaz-H*), 7.61 (t, 1 H, $J_{6,7}$ = 8.6, $J_{5,6}$ = 8.5 Hz, H-6, *Phthalaz-H*), 7.82 (d, 1 H, $J_{5,6}$ = 8.2 Hz, H-5, *Phthalaz-H*), 8.00-8.03 (2H, m, *Pyr-H*), 8.10 (d, 1H, $^3J_{HH}$ = 9.2 Hz, *Pyr-H*), 8.17 (d, 1H, $^3J_{HH}$ = 7.7 Hz, *Pyr-H*), 8.20-8.23 (m, 3H, *Pyr-H*), 8.31 (dd, 1H, $^3J_{HH}$ = 8.2 and $^4J_{HH}$ = 2.7 Hz, *Pyr-H*), 8.48 (d, 1H, $^3J_{HH}$ = 9.5 Hz, *Pyr-H*); ^{13}C NMR: δ 19.9, 20.2, 20.3 (3 CH_3), 37.8(CH_2), 61.3 (C-5′), 71.0 (C-2′), 73.6 (C-3′), 78.1 (C-4′) and 92.2 (C-1′), 121.0, 121.3, 122.5, 123.8, 124.2, 125.8, 125.9, 125.4, 126.1, 126.7, 127.1, 128.1, 128.5, 128.8, 129.1, 129.5, 130.6, 131.0, 131.4, 131.8, 132.3, 133.4, 137.1 (Ar-C), 160.5 (N=C-S), 163.2, 169.3, 170.1 (3 CO); FT-IR (KBr, *v*, cm^{-1}): 1743 (C=O ester) cm^{-1}, MS (m/z): 634 (M$^+$, 10). Anal.calcd. for $C_{36}H_{30}N_2O_7S$: C, 68.13; H, 4.76; N, 4.41; S, 5.05; found C, 68.18; H, 4.78; N, 4.41; S, 5.00.

Synthesis of 4-(Pyren-1-ylmethyl)-1-((2′,3′,4′,6′-tetra-*O*-acetyl-*β*-D-galactopyranosyl-(1→4))-(2′,3′,4′,6′ -tetra-*O*-acetyl-*β*-D-glucopyranosyl-1-thio))phthalazine (19): Yield: 53%, m.p.: 100–101°C; ^{1}H NMR (DMSO-d$_6$, δ): 1.90, 1.93, 1.96, 2.00, 2.04, 2.10, 2.18 (7s, 21H, 7 $OCOCH_3$), 4.00-4.09 (m, 3H, H-2′b, H-6′a, H-6′b), 4.20 (dd, 1H, $J_{6''a,6'a}$ = 10.8, $J_{6''a,5'a}$ = 4.3 Hz, H-6′′a), 4.40 (m, 1H, H-5′b), 4.66 (dd, 1H, $J_{6''b,5'b}$ = 7.1 Hz, H-6′′b), 4.73 (s, 2H, CH_2), 4.90 (m, 1H, H-5′a), 4.97 (dd, 1H, $J_{1'b,2'b}$ = 8.0 Hz, H-1′b), 5.12 (1H, $J_{4'b,3'b}$ = 4.0, $J_{4'b,5'b}$ = 4.6 Hz, H-4′b), 5.25 (dd, 1H, $J_{2'a,1'a}$ = 10.2, $J_{2'a,3'a}$ = 10.2 Hz, H-2′a), 5.30 (dd, 1H, $J_{4'a,3'a}$ = 8.5, $J_{4'a,5'a}$ = 8.9 Hz, H-4′a), 5.39 (d, 1H, $J_{3'b,4'b}$ = 4.1 Hz, H-3′b), 5.44 (dd, 1H, $J_{3'a,2'a}$ = 8.9, $J_{3'a,4'a}$ = 9.9 Hz, H-3′a), 6.40 (d, 1H, $J_{1'a,2'a}$ = 9.1 Hz, H-1′a), 7.50 (t, 1 H, $J_{6,7}$ = 7.8 Hz, H-7, *Phthalaz-H*), 7.62 (d, 1 H, *J* = 7.6 Hz, H-8, *Phthalaz-H*), 7.76 (t, 1 H, *J* = 8.6 Hz, H-6, *Phthalaz-H*), 7.83 (d, 1 H, $J_{5,6}$ = 8.6 Hz, H-5, *Phthalaz-H*), 8.04-8.07 (m, 2H, *Pyr-H*), 8.11 (d, 1H, $^3J_{HH}$ = 8.5 Hz, *Pyr-H*), 8.16 (d, 1H, $^3J_{HH}$ = 8.4 Hz, *Pyr-H*), 8.20-8.25 (m, 3H, *Pyr-H*), 8.27-8.29 (m, 1H, *Pyr-H*), 8.45 (d, 1H, $^3J_{HH}$ = 9.6 Hz, *Pyr-H*); FT-IR (KBr, *v*, cm^{-1}): 1745 (C=O ester) cm^{-1}. MS (m/z): 994 (M$^+$, 11). Anal.calcd. for $C_{51}H_{50}N_2O_{17}S$: C, 61.56; H, 5.07; N, 2.82; S, 3.22; found C, 61.56; H, 5.11; N, 2.85; S, 3.20.

Synthesis of 4-(Pyren-1-ylmethyl)-1-(*β*-D-glucopyranosylthio)phthalazine (16): Yield: 50%, m.p.: 144–145°C; ^{1}H NMR (DMSO-d$_6$, δ): 3.31 (m, 6H, H-6′ , H-6′′, H-5′ , H-4′, H-3′and H-2′), 3.50 (t, 1H, *J*= 4.1 Hz, OH-6′, exchangeable with D_2O), 4.40 (d, 1H, *J*= 5.3 Hz, OH-4′, exchangeable with D_2O), 4.73 (s, 2H, CH_2), 5.19 (d, 1H, *J*= 5.2 Hz, OH-3′,D_2O exchangeable), 5.61 (d, 1H, *J*= 3.8 Hz, OH-2′, exchangeable with D_2O), 6.11 (d, 1H, $J_{1',2'}$= 9.0 Hz, H-1′), 7.46 (t, 1 H, $J_{6,7}$ = 7.5 Hz, H-7, *Phthalaz-H*), 7.51 (d, 1 H, $J_{7,8}$ = 7.5 Hz, H-8, *Phthalaz-H*), 7.67 (t, 1 H, H-6, *Phthalaz-H*), 7.85 (d, 1 H, $J_{5,6}$ = 8.2 Hz, H-5, *Phthalaz-H*), 8.01 (dd, 1H, $^3J_{HH}$ = 7.4 and 7.0 Hz, *Pyr-H*), 8.08-8.13 (m, 2H, *Pyr-H*), 8.18 (d, 1H, $^3J_{HH}$ = 9.5 Hz, *Pyr-H*), 8.22 (d, 2H, $^3J_{HH}$ = 7.4 Hz, *Pyr-H*), 8.29 (d, 1H, $^3J_{HH}$ = 8.3 Hz, *Pyr-H*), 8.35-8.44 (m, 2H, *Pyr-H*); ^{13}C NMR: δ 37.8(CH_2), 61.0 (C-6′), 68.2 (C-4′), 70.1 (C-2′), 72.9 (C-3′), 79.3 (C-5′), 98.9 (C-1′), 122.0, 122.2, 122.5, 123.2, 124.1, 125.4, 125.8, 125.9, 126.2, 126.8, 127.3, 128.2, 128.5, 128.9, 129.3, 129.5, 130.3, 131.0, 131.6, 131.8, 132.1, 133.5, 136.0 (Ar-C), 159.1 (N=C-S).; FT-IR (KBr, *v*, cm^{-1}): 3478-3210 (OH) cm^{-1}. MS (m/z): 538 (M$^+$, 18). Anal.calcd. for $C_{31}H_{26}N_2O_5S$: C, 69.13; H, 4.87; N, 5.20; S, 5.95; found C, 69.19; H, 4.85; N, 5.20; S, 5.90.

Synthesis of 4-(Pyren-1-ylmethyl)-1-(*β*-D-xylofuranosylthio)phthalazine (18): Yield: 61%, m.p.:133–134°C; ^{1}H NMR (DMSO-d$_6$, *δ*): 3.39 (m, 2H, H-5′, H-5′′), 3.42 (m, 1H, H-4′), 3.50-3.66 (2H, H-3′, H-2′), 4.55 (t, 1H, *J* = 7.0 Hz, OH-5′, exchangeable with D_2O), 4.68 (d, 1H, *J* = 5.2 Hz, OH-3′, exchangeable with D_2O), 4.72 (s, 2H, CH_2), 4.80 (d, 1H, *J* = 3.5 Hz, OH-2′, exchangeable with D_2O), 6.13 (d, 1H, $J_{1',2'}$ = 3.5 Hz, H-1′), 7.57 (m, 2 H, H-7,8, *Phthalaz-H*), 7.66 (t, 1 H, $J_{6,7}$ = 8.7, $J_{5,6}$ = 8.6 Hz, H-6, *Phthalaz-H*), 7.89 (d, 1 H, $J_{5,6}$ = 8.7 Hz, H-5, *Phthalaz-H*), 8.03 (t, 1H, $^3J_{HH}$ = 7.7 Hz, *Pyr-H*); 8.05-8.09 (m, 2H *Pyr-H*); 8.14 (d, 1H, $^3J_{HH}$ = 8.2 Hz, *Pyr-H*); 8.19 (d, 2H, $^3J_{HH}$ = 7.7 Hz, *Pyr-H*); 8.24 (d, 2H, $^3J_{HH}$ = 8.2 Hz, *Pyr-H*); 8.37 (dd, 1H, $^3J_{HH}$ = 8.2 and $^4J_{HH}$ = 2.3 Hz, *Pyr-H*); 8.43-8.47 (m, 1H, *Pyr-H*); ^{13}C NMR: *δ* 37.4(CH_2), 63.2 (C-5′), 71.8 (C-3′), 77.9 (C-2′), 81.2 (C-4′) and 99.2 (C-1′), 121.3, 122.0, 122.5, 123.0, 124.2, 125.2, 125.7, 125.8, 126.1, 126.7, 127.1, 128.0, 128.3, 128.8, 129.0, 129.4, 130.3, 131.1, 131.5, 131.7, 132.0, 133.1, 135.3 (Ar-C), 155.3 (N=C-S). FT-IR (KBr, *v*, cm^{-1}): 3490-3244 (OH) cm^{-1}. MS (m/z): 508 (M^+, 19). Anal.calcd. for $C_{30}H_{24}N_2O_4S$: C, 70.85; H, 4.76; N, 5.51; S, 6.30; found C, 70.90; H, 4.76; N, 5.56; S, 6.28.

Synthesis of 4-(Pyren-1-ylmethyl)-1-(*β*-D-lactosylthio) phthalazine (20): Yield: 52%, m.p.: 200–201°C; ^{1}H NMR (DMSO-d$_6$, *δ*): 3.32-3.59 (4 m,12H, H-2′b, H-3′b, H-4′b, H-5′b, H-6′b, H-6′′b, H-2′a, H-3′a, H-4′a, H-5′a, H-6′a, H-6′′a), 4.39 (d, 1H, OH-4′b, exchangeable with D_2O), 4.52 (d, 1H, OH-6′b, exchangeable with D_2O), 4.71 (s, 2H, CH_2), 4.80 (d, 1H, *J* = 5.1 Hz, OH-3′b), 4.91 (d, 1H, OH-2′b), 5.11 (d, 1H, OH-6′a, exchangeable with D_2O), 5.13 (d, 1H, OH-3′a, exchangeable with D_2O), 5.34 (d, 1H, OH-2′a, exchangeable with D_2O), 5.77(d, 1H, $J_{1'b,2'b}$= 8.0 Hz, H-1′b), 5.85 (d, 1H, $J_{1'a,2'a}$= 9.1 Hz,H-1′a), 7.26 (d, ^3J = 7.1 Hz, 1H, *Pyrene -H*), 7.54 (d, ^3J = 7.8 Hz, 1H, *Pyrene -H*), 7.60 (t, 1H, $J_{6,7}$ 5.2 Hz, H-7, *Phthalazine-H*), 7.75 (d, 1H, $J_{7,8}$ 7.0 Hz, H-8, *Phthalazine-H*), 7.91 (t, 1H, H-6, *Phthalazine-H*), 8.10-8.40 (m, 7H, *Pyrene-H*), 8.51 (d, 1H, $J_{5,6}$ 8.3 Hz, H-5, *Phthalazine-H*); 7.48 (t, 1 H, $J_{6,7}$ = 7.5 Hz, H-7, *Phthalaz-H*), 7.60 (d, 1 H, *J* = 7.5 Hz, H-8, *Phthalaz-H*), 7.79 (t, 1 H, *J* = 8.0 Hz, H-6, *Phthalaz-H*), 7.88 (d, 1 H, $J_{5,6}$ = 8.2 Hz, H-5, *Phthalaz-H*), 8.02 (t, 1H, $^3J_{HH}$ = 7.5 Hz, *Pyr-H*), 8.07- 8.09 (m, 2H *Pyr-H*), 8.15 (d, 1H , $^3J_{HH}$ = 9.2 Hz, *Pyr-H*), 8.21-8.24 (m, 2H *Pyr-H*), 8.29 (d, 2H , $^3J_{HH}$ = 8.4 Hz, *Pyr-H*), 8.41-8.44 (m, 1H *Pyr-H*); ^{13}C NMR: *δ* 36.9(CH_2), 60.2 (C-6′a), 60.7 (C-6′b), 67.0 (C-3′a), 69.2 (C-2′b), 71.0 (C-2′a), 73.8 (C-3′b), 74.9 (C-4′a), 76.5 (C-4′b), 79.3 (C-5′b), 80.0 (C-5′a), 97.1 (C-1′b), 101.1 (C-1′a). 121.0, 121.7, 122.5, 122.8, 124.1, 125.3, 125.6, 125.9, 126.0, 126.5, 127.1, 127.7, 128.3, 128.7, 129.2, 129.5, 130.3, 131.0, 131.4, 131.7, 132.3, 133.5, 134.9 (Ar-C), 150.2 (N=C-S), FT-IR (KBr, *v*, cm^{-1}): 3471-3198 (OH) cm^{-1}; MS (m/z): 700 (M^+, 15). Anal.calcd. for $C_{37}H_{36}N_2O_{10}S$: C, 63.42; H, 5.18; N, 4.00; S, 4.58; found C, 63.49; H, 5.10; N, 4.00; S, 4.55.

Synthesis of Ethyl 2-(1-oxo-4-(pyren-1-ylmethyl) phthalazin-2(1*H*)-yl)propanoate (21): Yield: 77%, m.p.: 139–140°C; ^{1}H NMR (DMSO-d$_6$, *δ*): 1.12 (t, 3H, *J* = 10 Hz, CH_3CH_2), 1.61 (d, 3H, *J* = 8.1 Hz CH_3CH), 4.20 (q, 2H, *J* = 10 Hz CH_2CH_3), 4.73 (s, 2H, CH_2), 4.86 (q, 1H, *J* =8.0 Hz $CHCH_3$), 7.52 (m, 2 H, H-7,8, *Phthalaz-H*), 7.63 (t, 1 H, $J_{6,7}$ = 8.0, $J_{5,6}$ = 8.1 Hz, H-6, *Phthalaz-H*), 7.87 (d, 1 H, $J_{5,6}$ = 8.5 Hz, H-5, *Phthalaz-H*), 8.05 (t, 1H, $^3J_{HH}$ = 7.5 Hz, *Pyr-H*); 8.08-8.12 (m, 2H, *Pyr-H*); 8.17 (d, 1H, $^3J_{HH}$ = 9.1 Hz, *Pyr-H*); 8.22 (d,

2H, $^3J_{HH}$ = 7.5 Hz, *Pyr-H*); 8.28 (d, 1H, $^3J_{HH}$ = 8.2 Hz, *Pyr-H*); 8.33-8.39 (m, 2H, *Pyr-H*); ^{13}C NMR: δ 13.8 (CH_3), 15.2 (CH_3), 36.8 (CH_2), 65.2 (OCH_2), 67.1 (CH), 122.1, 122.5, 123.2, 124.0, 124.7, 125.3, 125.7, 125.9, 126.2, 126.5, 127.3, 127.9, 128.3, 128.8, 129.1, 129.5, 130.1, 131.4, 131.7, 131.9, 132.3, 133.9, 140.9 (Ar-C), 159.1 (N-C=O), 174.5(O-C=O); FT-IR (KBr, v,cm^{-1}): 1747 (C=O ester), 1670(C=O) cm^{-1}; MS (m/z):460 (M^+, 20). Anal.calcd. for $C_{30}H_{24}N_2O_3$: C, 78.24; H, 5.25; N, 6.08; found C, 78.30; H, 5.28; N, 6.01.

Synthesis of 2-(1-Oxo-4-(pyren-1-ylmethyl) phthalazin-2(1*H*)-yl)propanehydrazide (22): Yield: 70%, m.p.: 220–221°C; ^{1}H NMR (DMSO-d$_6$, δ): 1.67 (d, 3H, *J* = 8.1 Hz CH_3CH), 4.46 (s, 2H, NH_2 exchangeable with D_2O), 4.70 (s, 2H, CH_2), 4.90 (q, 1H, *J* = 8.0 Hz $CHCH_3$), 7.48 (t, 1 H, $J_{6,7}$ = 7.5 Hz, H-7, *Phthalaz-H*), 7.59 (d, 1 H, *J* = 7.5 Hz, H-8, *Phthalaz-H*), 7.72 (t, 1H, *J* = 8.0 Hz, H-6, *Phthalaz-H*), 7.88 (d, 1 H, $J_{5,6}$ = 8.4 Hz, H-5, *Phthalaz-H*), 8.04 (t, 1H, $^3J_{HH}$ = 7.5 Hz, *Pyr-H*); 8.06-8.09 (m, 3H, *Pyr-H*), 8.18-8.21 (m, 3H, *Pyr-H*), 8.29 (d, 1H, $^3J_{HH}$ = 7.8 Hz, *Pyr-H*), 8.42-8.45 (m, 1H, *Pyr-H*), 9.32 (s, 1H, NH exchangeable with D_2O); ^{13}C NMR: δ 15.9 (CH_3), 37.2 (CH_2), 68.2 (CH), 122.3, 122.4, 123.1, 123.9, 124.2, 125.3, 125.8, 125.9, 126.4, 126.6, 127.4, 127.9, 128.5, 128.8, 129.4, 129.7, 130.7, 131.9, 132.0, 132.6, 132.9, 133.5, 144.2 (Ar-C), 155.2 (N-C=O), 171.8 (NH-C=O); FT-IR (KBr, v,cm^{-1}): 3343, 3218, 3103 ($NHNH_2$), 1672, 1665 (2C=O) cm^{-1}; MS (m/z):446 (M^+, 38). Anal.calcd. for $C_{28}H_{22}N_4O_2$: C, 75.32; H, 4.97; N, 12.55; found C, 75.30; H, 4.92; N, 12.50.

Synthesis of 2-(1-(5-(D-Gluco-pentitol-1-yl)-1,3,4-oxadiazol-2-yl)ethyl)-4-(pyren-1-ylmethyl) phthalazin-1(2*H*)-one (23a): Yield: 90%, m.p.: 211–212°C; ^{1}H NMR (DMSO-d$_6$, δ): 1.60 (d, 3H, *J*=8.0 Hz CH_3CH), 3.19 (d, 1H, $J_{3',4'}$ 8.28 Hz H-3′), 3.35 (dd, 1H, $J_{4',5''}$ 5.7 Hz H-5′′), 3.50 (m, 1H, $J_{4',5'}$ 2.52 Hz H-4′), 3.57 (dd, 1H, $J_{5',5''}$ 11,3 Hz H-5′), 4.19(d,1 H, $J_{2',3'}$ 3.1 Hz, H-2′), 4.30 (m, 3H, OH exchangeable with D_2O), 4.61 (d, 1H, OH exchangeable with D_2O), 4.72 (s, 2H, CH_2), 4.84 (d, 1H, $J_{1',2'}$7.42 Hz H-1′), 4.92 (q, 1H, *J* = 8.5 Hz $CHCH_3$), 5.10(s, 1H, OH exchangeable with D_2O), 7.51 (t, 1 H, $J_{6,7}$ = 7.8 Hz, H-7, *Phthalaz-H*), 7.62 (d, 1 H, *J* = 7.8 Hz, H-8, *Phthalaz-H*), 7.80 (t, 1 H, *J* = 8.6 Hz, H-6, *Phthalaz-H*), 7.84 (d, 1 H, $J_{5,6}$ = 8.9 Hz, H-5, *Phthalaz-H*), 8.04 (t, 1H $^3J_{HH}$ = 7.8 Hz, *Pyr-H*), 8.11 (d, 1H $^3J_{HH}$ = 9.0 Hz, *Pyr-H*), 8.15 (d, 1H $^3J_{HH}$ = 9.0 Hz, *Pyr-H*), 8.20 (d, 1H $^3J_{HH}$ = 8.5 Hz, *Pyr-H*), 8.23-8.26 (m, 3H *Pyr-H*), 8.41 (d, 1H $^3J_{HH}$ = 8.2 Hz, *Pyr-H*), 8.53 (d, 1H $^3J_{HH}$ = 9.5 Hz, *Pyr-H*); ^{13}C NMR: δ 17.3(CH_3), 59.6 (CH), 36.5(CH_2), 64.2 (C-5′), 67.6 (C-1′), 71.6 (C-3′), 71.8 (C-2′), 72.2 (C-4′), 121.5, 122.1, 123.0, 123.7, 124.5, 125.3, 125.7, 125.9, 126.2, 126.6, 127.3, 127.8, 128.6, 128.8, 129.1, 129.5, 130.4, 131.6, 132.1, 132.5, 132.9, 133.2, 146.1 (Ar-C), 152.1 (N-C=O), 156.3, 160.8 (2C-oxadiazole); FT-IR (KBr, v,cm^{-1}): 3490-3198 (OH), 1670 (C=O), 1612 cm^{-1}. MS (m/z):608 (M^+, 13). Anal.calcd. for $C_{34}H_{32}N_4O_7$: C, 67.09; H, 5.30; N, 9.21; found C, 67.20; H, 5.20; N, 9.19.

Synthesis of 2-(1-(5-(D-Galacto-pentitol-1-yl)-1,3,4-oxadiazol-2-yl)ethyl)-4-(pyren-1-ylmethyl) phthalazin -1(2*H*)-one (23b): Yield: 92%, m.p.: 230–231°C; ^{1}H NMR (DMSO-d$_6$, δ): 1.68 (d, 3H, *J*=8.0 Hz CH_3CH), 3.38(t, 2H, H-5,′5′′), 3.50(t, 1H, H-4′), 3.71 (dd, 1H, H-3′), 3.82(m, 1H, H-2), 4.20(m,2 H, OH exchangeable with D_2O), 4.35(dd,1 H, OH exchangeable with D_2O), 4.62(d,1 H, OH exchangeable with D_2O), 4.76 (s, 2H, CH_2), 4.90 (q, 1H, *J* =8.5 Hz $CHCH_3$), 5.02(d, 1H, H-1′), 5.28(d,1 H, OH exchangeable

with D_2O), 7.50 (t, 1 H, $J_{6,7}$ = 7.7 Hz, H-7, *Phthalaz-H*), 7.62 (d, 1 H, *J* = 7.7 Hz, H-8, *Phthalaz-H*), 7.70 (t, 1H, *J* = 8.4 Hz, H-6, *Phthalaz-H*), 7.85 (d, 1 H, $J_{5,6}$ = 8.6 Hz, H-5, *Phthalaz-H*), 7.96-8.01 (m, 1H *Pyr-H*); 8.12 (t, 1H $^3J_{HH}$ = 7.4 Hz, *Pyr-H*); 8.19 (d, 1H $^3J_{HH}$ = 9.0 Hz, *Pyr-H*); 8.24 (d, 1H $^3J_{HH}$ = 8.8 Hz, *Pyr-H*); 8.29 (d, 1H $^3J_{HH}$ = 8.7 Hz, *Pyr-H*); 8.36 (d, 1H $^3J_{HH}$ = 7.4 Hz, *Pyr-H*); 8.39 (d, 1H $^3J_{HH}$ = 7.5 Hz, *Pyr-H*); 8.42 (d, 1H $^3J_{HH}$ = 7.8 Hz, *Pyr-H*); 8.45-8.49 (m, 1H *Pyr-H*); ^{13}C NMR: δ 17.0(CH_3), 60.1 (CH), 36.9(CH_2), 65.20 (C-5′), 66.7 (C-1′), 71.7 (C-3′), 71.9 (C-2′), 72.5 (C-4′), 121.0, 121.8, 123.2, 123.7, 124.2, 125.4, 125.7, 125.9, 126.0, 126.4, 127.3, 127.9, 128.6, 128.9, 129.2, 129.5, 130.2, 131.6, 132.3, 132.5, 132.9, 133.4, 145.2 (Ar-C), 154.1 (N-C=O), 155.1, 159.2 (2C-oxadiazole); FT-IR (KBr, *v*,cm^{-1}): 3481-3223 (OH), 1668 (C=O), 1613 cm^{-1}. MS (m/z): 608 (M^+, 17). Anal.calcd. for $C_{34}H_{32}N_4O_7$: C, 67.09; H, 5.30; N, 9.21; found C, 67.22; H, 5.18; N, 9.20.

Synthesis of 2-(1-(5-(D-Glycero-D-gulo-hexitol-1-yl)-1,3,4-oxadiazol-2-yl)ethyl)-4-(pyren-1-ylmethyl) phthalazin-1(2*H*)-one (24): Yield: 87%, m.p.: 288–289°C; ^{1}H NMR (DMSO-d_6, δ): 1.68 (d, 3H, *J*=8.0 Hz CH_3CH), 3.1-3.4 (m, 3 H, H-5′, 6′, 6′′), 3.90-4.10 (m, 2 H, H-3′ and H-4′), 4.30, 4.52, 5.39 (m, s, d, 6 H, 6OH exchangeable with D_2O), 4.72 (s, 2H, CH_2), 4.89-4.80 (m, 2 H, H-1′ and H-2′), 4.96 (q, 1H, *J* =8.5 Hz $CHCH_3$), 7.50 (m, 2 H, H-7,8, *Phthalaz-H*), 7.69 (t, 1 H, $J_{6,7}$ = 7.5, $J_{5,6}$ = 7.5 Hz, H-6, *Phthalaz-H*), 7.85 (d, 1 H, $J_{5,6}$ = 7.7 Hz, H-5, *Phthalaz-H*), 8.01 (t, 1H, $^3J_{HH}$ = 7.7 Hz, *Pyr-H*), 8.04-8.07 (m, 2H, *Pyr-H*), 8.19 (d, 1H, $^3J_{HH}$ = 9.3 Hz, *Pyr-H*), 8.25 (d, 2H, $^3J_{HH}$ = 9.3 Hz, *Pyr-H*), 8.30 (d, 1H, $^3J_{HH}$ = 8.0 Hz, *Pyr-H*), 8.40 (d, 1H, $^3J_{HH}$ = 9.3 Hz, *Pyr-H*), 8.43-8.47 (m, 1H, *Pyr-H*); ^{13}C NMR: δ 16.8(CH_3), 60.5 (CH), 37.2(CH_2), 63.2 (C-6′), 65.7 (C-1′), 67.0 (C-3′), 72.1 (C-4′), 72.9 (C-2′), 73.9 (C-5′), 121.3, 121.9, 123.3, 123.8, 124.1, 125.5, 125.7, 125.9, 126.2, 126.5, 127.0, 127.7, 128.3, 128.8, 129.1, 129.5, 130.4, 131.8, 132.1, 132.5, 132.8, 133.1, 147.4 (Ar-C), 155.5 (N-C=O), 156.3, 159.8 (2C-oxadiazole); FT-IR (KBr, *v*,cm^{-1}): 3403-3121 (OH), 1677(C=O), 1610 cm^{-1}; MS (m/z): 638 (M^+, 13). Anal.calcd. for $C_{35}H_{34}N_4O_8$: C, 66.82; H, 5.37; N, 8.77; found C, 66.70; H, 5.13; N, 8.70.

Synthesis of Preparation of double headed acyclo *C*-nucleoside analogous 25: Yield: 67%, m.p.: 290–291°C; ^{1}H NMR (DMSO-d_6, δ): 1.70 (d, 3H, *J*=8.0 Hz CH_3CH), 3.89 (d, 2 H, H-2′and H3′), 4.70 (s, 2H, CH_2), 4.90 (d, 2 H, H-1′ and H-4′), 4.90, 4.95 and 5.29 (d, s and d, 4 H, D_2O exchangable, 4OH), 7.52 (t, 1H, $J_{6,7}$ = 7.2 Hz, H-7, *Phthalazi-H*), 7.65 (d, 1H, $J_{7,8}$ = 7.4 Hz, H-8, *Phthalaz-H*), 7.80 (t, 1H, H-6, *Phthalaz-H*), 7.86 (d, 1H, $J_{5,6}$=8.0 Hz, H-5, *Phthalaz-H*), 8.02 (dd, 1H, $^3J_{HH}$ = 7.4 and 7.0 Hz, *Pyr-H*), 8.06-8.12 (m, 2H, *Pyr-H*), 8.20 (d, 1H, $^3J_{HH}$ = 9.5 Hz, *Pyr-H*), 8.26 (d, 2H, $^3J_{HH}$ = 8.0 Hz, *Pyr-H*), 8.30 (d, 1H, $^3J_{HH}$ = 8.6 Hz, *Pyr-H*), 8.41-8.46 (m, 2H, *Pyr-H*), ^{13}C NMR: δ 18.0(2CH_3), 63.1 ($_2$CH), 37.5(2CH_2), 72.3(C-2′, C-3′), 76.4 (C-1′, C-4′), 121.2, 121.6, 123.0, 123.4, 124.1, 125.3, 125.5, 126.0, 126.5, 126.7, 127.2, 127.9, 128.1, 128.8, 129.4, 129.5, 130.1, 131.6, 132.2, 132.5, 132.9, 133.2, 148.8 (Ar-C), 154.1 (2N-C=O), 156.1, 158.9 (4C-oxadiazole), FT-IR (KBr, *v*,cm^{-1}): 3466-3178 (OH), 1669 (C=O), 1614 cm^{-1}. MS (m/z): 1030 (M^+, 9). Anal.calcd. for $C_{62}H_{46}N_8O_8$: C, 72.22; H, 4.50; N, 10.87; found C, 72.20; H, 4.47; N, 10.89.

Synthesis ofD-Glucose -2-(4-(pyren-1-ylmethyl)phthalazin-1(2*H*)-one)propionohydrazone(26a).: Yield: 80%, m.p.: 201–202°C; ^{1}H NMR (DMSO-d$_6$, *δ*): 1.70 (d, 3H, *J*=8.0 Hz CH_3CH), 3.20 (d, 1H, $J_{3',4'}$ 8.2 Hz H-4′), 3.32 (dd, 1H, $J_{4',5''}$ 5.2 Hz H-6′′), 3.52 (m, 1H, $J_{4',5'}$ 2.5 Hz H-5′), 3.59 (dd, 1H, $J_{5',5''}$ 11.1 Hz H-6′), 4.20(d,1 H, $J_{2',3'}$ 3.1 Hz H-3′), 4.31 (m, 3H, OH exchangeable with D_2O), 4.64 (d, 1H, OH exchangeable with D_2O), 4.72 (s, 2H, CH_2), 4.83 (dd, *J* = 7.4 Hz, *J* = 7.8 Hz, 1H, H-2′), 5.00 (q, 1H, *J* =8.5 Hz $CHCH_3$), 5.54(s, 1H, OH exchangeable with D_2O), 7.01 (d, *J* = 7.8 Hz, 1H, H-1′), 7.44 (m, 2 H, H-7,8, *Phthalaz-H*), 7.69 (t, 1 H, $J_{6,7}$ = 8.7, $J_{5,6}$ 8.2 Hz, H-6, *Phthalaz-H*), 7.80 (d, 1 H, $J_{5,6}$ = 7.4 Hz, H-5, *Phthalaz-H*), 8.06 (t, 1H, $^3J_{HH}$ = 7.6 Hz, *Pyr-H*), 8.08-8.10 (m, 2H, *Pyr-H*), 8.17 (d, 1H $^3J_{HH}$ = 9.2 Hz,), 8.25 (d, 2H, $^3J_{HH}$ = 7.9 Hz, *Pyr-H*), 8.29 (d, 1H, $^3J_{HH}$ = 8.3 Hz, *Pyr-H*), 8.42 (d, 1H, $^3J_{HH}$ = 7.6 Hz, *Pyr-H*), 8.48 (d, 1H, $^3J_{HH}$ = 8.4 Hz, *Pyr-H*); 10.12 (s, 1H, NH); FT-IR (KBr, *v*,cm^{-1}): 3475-3188 (broad, OH+NH), 1677 (C=O), 1625 (CH=N) cm^{-1}; MS (m/z): 608 (M$^+$, 8). Anal.calcd. for $C_{34}H_{32}N_4O_7$: C, 67.09; H, 5.30; N, 9.21; found C, 67.11; H, 5.35; N, 9.24.

3.27. Synthesis of D-Galactose -2-(4-(pyren-1-ylmethyl) phthalazin-1(2*H*)-one)propionohydrazone (26b): Yield: 77%, m.p.: 214–215°C; ^{1}H NMR (DMSO-d$_6$, *δ*): 1.66 (d, 3H, *J*=8.0 Hz CH_3CH), 3.30(t, 2H, H-6′,6′′), 3.50(t, 1H, H-5′), 3.70 (dd, 1H, H-4′), 3.85(m, 1H, H-3′), 4.19(m,2 H, OH exchangeable with D_2O), 4.32(dd,1 H, OH exchangeable with D_2O), 4.35 (dd, *J* = 7.4 Hz, *J* = 7.8 Hz, 1H, H-2′), 4.60(d,1 H, OH exchangeable with D_2O), 4.72 (s, 2H, CH_2), 5.03 (q, 1H, *J* =8.5 Hz $CHCH_3$), 5.27(d,1 H, OH exchangeable with D_2O), 7.57 (m, 2 H, H-7,8, *Phthalaz-H*), 7.62 (t, 1 H, $J_{6,7}$ = 8.3, $J_{5,6}$ = 8.1 Hz, H-6, *Phthalaz-H*), 7.84 (d, 1 H, $J_{5,6}$ = 8.0 Hz, H-5, *Phthalaz-H*), 7.96-7.99 (2H, m, *Pyr-H*), 8.09 (d, 1H, $^3J_{HH}$ = 9.0 Hz, *Pyr-H*), 8.14 (d, 1H, $^3J_{HH}$ = 7.5 Hz, *Pyr-H*), 8.17-8.19 (m, 3H, *Pyr-H*), 8.32 (dd, 1H, $^3J_{HH}$ = 8.0 and $^4J_{HH}$ = 2.5 Hz, *Pyr-H*), 8.43 (d, 1H, $^3J_{HH}$ = 9.0 Hz, *Pyr-H*); 10.17 (s, 1H, NH exchangeable with D_2O); ^{13}C NMR: δ 14.9(CH_3), 37.1(CH_2), 60.4 (CH), 63.0 (C-6′), 69.2 (C-4′), 69.8 (C-3′), 72.4 (C-2′), 72.6 (C-5′), 121.0, 121.8, 123.4, 123.8, 124.4, 125.6, 125.7, 125.9, 126.0, 126.5, 127.1, 127.5, 128.3, 128.9, 129.1, 129.7, 130.4, 131.4, 132.1, 132.6, 132.8, 133.3, 145.4 (Ar-C), 150.1, (CH=N), 155.5 (N-C=O); FT-IR (KBr, *v*,cm^{-1}): 3486-3122 (broad, OH+NH), 1670 (C=O), 1631 (CH=N) cm^{-1}; MS (m/z): 608 (M$^+$, 9. Anal.calcd. for $C_{34}H_{32}N_4O_7$: C, 67.09; H, 5.30; N, 9.21; found C, 67.15; H, 5.36; N, 9.19.

Synthesis of D-Mannose -2-(4-(pyren-1-ylmethyl)phthalazin-1(2*H*)-one)propionohydrazone(26c): Yield: 75%, m.p.: 195–196°C; ^{1}H NMR (DMSO-d$_6$, *δ*): 1.71 (d, 3H, *J*=8.0 Hz CH_3CH), 3.27-3.37 (m, 2H, H-6′,6′′), 3.75 (m, 1H, H-5′), 4.13 (m, 1H, H-4′), 4.30(t, *J* = 7.4 Hz, 1H, H-3′), 4.40 (dd, *J* = 7.4 Hz, *J* = 7.8 Hz, 1H, H-2′), 4.48 (m, 1H, OH exchangeable with D_2O), 4.53 (d, *J* = 6.4 Hz, 1H, OH exchangeable with D_2O), 4.72 (s, 2H, CH_2), 5.07 (q, 1H, *J* =8.5 Hz $CHCH_3$), 5.20 (m, 1H, OH exchangeable with D_2O), 5.60 (t, *J* = 4.6 Hz, 1H, OH), 5.83 (t, *J* = 4.6 Hz, 1H, OH exchangeable with D_2O), 7.04 (d, *J* = 7.8 Hz, 1H, H-1′), 7.46 (m, 2 H, H-7,8, *Phthalaz-H*), 7.60 (t, 1 H, $J_{6,7}$ = 7.7, $J_{5,6}$ = 7.8 Hz, H-6, *Phthalaz-H*), 7.86 (d, 1 H, $J_{5,6}$ = 7.9 Hz, H-5, *Phthalaz-H*), 8.03 (t, 1H, $^3J_{HH}$ = 7.6 Hz, *Pyr-H*), 8.04-8.07 (m, 2H, *Pyr-H*), 8.15 (d,1H, $^3J_{HH}$ = 9.0 Hz, *Pyr-H*),, 8.20 (d, 2H, $^3J_{HH}$ = 9.0 Hz, *Pyr-H*), 8.24 (d,1 H, $^3J_{HH}$ = 8.2 Hz, *Pyr-H*), 8.39 (d, 1H, $^3J_{HH}$ = 9.0 Hz, *Pyr-H*),

8.40-8.43 (m, 1H, *Pyr-H*), 10.10 (s, 1H, NH exchangeable with D_2O); ^{13}C NMR: δ 15.1(CH_3), 37.3(CH_2), 60.7 (CH), 63.0 (C-6′), 69.4 (C-4′), 70.4 (C-3′), 70.9 (C-2′), 71.2 (C-5′), 121.3, 121.8, 122.9, 123.5, 124.4, 125.0, 125.7, 126.1, 126.2, 126.5, 127.0, 127.5, 128.3, 128.8, 129.3, 129.7, 130.3, 131.4, 132.2, 132.6, 132.9, 133.3, 146.1 (Ar-C), 150.4, (CH=N), 156.0 (N-C=O); FT-IR (KBr, v,cm^{-1}): 3501-3210 (broad, OH+NH), 1672 (C=O), 1635 (CH=N) cm^{-1}. MS (m/z): 608 (M^+, 11). Anal.calcd. for $C_{34}H_{32}N_4O_7$: C, 67.09; H, 5.30; N, 9.21; found C, 67.16; H, 5.38; N, 9.22.

Synthesis of D-Arabinose -2-(4-(pyren-1-ylmethyl) phthalazin-1(2*H*)-one)propionohydrazone (26d): Yield: 66%, m.p.: 233–234°C; ^{13}C NMR: δ 14.7(CH_3), 36.7(CH_2), 59.7 (CH), 63.5 (C-4′), 70.7 (C-3′), 71.0 (C-2′), 73.6 (C-4′), 121.1, 121.6, 122.1, 123.2, 124.3, 125.1, 125.7, 126.2, 126.6, 126.8, 127.2, 127.4, 128.0, 128.7, 129.0, 129.7, 130.2, 131.7, 132.4, 132.5, 132.9, 133.3, 147.1 (Ar-C), 150.2, (CH=N), 154.9 (N-C=O); FT-IR (KBr, v,cm^{-1}): 3490-3108(broad, OH+NH), 1668 (C=O), 1625(CH=N) cm^{-1}; MS (m/z): 578 (M^+, 10). Anal.calcd. for $C_{33}H_{30}N_4O_6$: C, 68.50; H, 5.23; N, 9.68; found C, 68.54; H, 5.29; N, 9.62.

Synthesis of D-Xylose -2-(4-(pyren-1-ylmethyl)phthalazin-1(2*H*)-one)propionohydrazone(26e). :Yield: 73%, m.p.: 191–192°C; ^{13}C NMR: δ15.1(CH_3), 37.3(CH_2), 60.7 (CH), 62.6 (C-5′), 71.7 (C-3′), 71.9 (C-2′), 72.3 (C-4′), 121.2, 121.5, 122.5, 123.5, 124.4, 125.1, 125.7, 126.4, 126.6, 126.9, 127.1, 127.3, 128.0, 128.8, 129.3, 129.9, 130.2, 131.7, 132.2, 132.5, 132.9, 133.5, 145.2 (Ar-C), 149.5, (CH=N), 156.0 (N-C=O); FT-IR (KBr, v,cm^{-1}): 3490-3118 (broad, OH+NH), 1673 (C=O), 1630(CH=N) cm^{-1}; MS (m/z): 578 (M^+, 6). Anal.calcd. for $C_{33}H_{30}N_4O_6$: C, 68.50; H, 5.23; N, 9.68; found C, 68.59; H, 5.26; N, 9.60.

Synthesis of D-Ribose -2-(4-(pyren-1-ylmethyl)phthalazin-1(2*H*)-one)propionohydrazone(26f). : Yield: 70%, m.p.: 178–179°C; ^{1}H NMR (DMSO-d$_6$, δ): 1.69 (d, 3H, J=8.0 Hz CH_3CH), 3.30-3.44 (m, 2H, H-5′,5′′), 3.61 (m, 1H, H-4′), 4.30 (t, J = 7.4 Hz, 1H, H-3′), 4.82 (dd, J = 7.4 Hz, J = 7.8 Hz, 1H, H-2′), 4.50 (d, J = 6.4 Hz, 1H, OH exchangeable with D_2O), 4.56 (m, 1H, OH exchangeable with D_2O), 4.70 (s, 2H, CH_2), 5.03 (q, 1H, J =8.5 Hz $CHCH_3$), 5.30 (t, J = 4.6 Hz, 1H, OH exchangeable with D_2O), 5.60 (t, J = 4.6 Hz, 1H, OH exchangeable with D_2O), 6.99 (d, J = 7.8 Hz, 1H, H-1′), 7.56 (m, 2 H, H-7,8, *Phthalaz-H*), 7.66 (t, 1 H, $J_{6,7}$ = 7.8, $J_{5,6}$ = 7.8 Hz, H-6, *Phthalaz-H*), 7.89 (d, 1 H, $J_{5,6}$ = 7.7 Hz, H-5, *Phthalaz-H*), 8.02 (t, 1H, $^3J_{HH}$ = 7.6 Hz, *Pyr-H*), 8.03-8.08 (m, 2H, *Pyr-H*), 8.21 (d, 1H, $^3J_{HH}$ = 9.2 Hz, *Pyr-H*), 8.26 (d, 2H, $^3J_{HH}$ = 9.2 Hz, *Pyr-H*), 8.30 (d, 1H, $^3J_{HH}$ = 8.0 Hz, *Pyr-H*), 8.42 (d, 1H, $^3J_{HH}$ = 9.2 Hz, *Pyr-H*), 8.44-8.48 (m, 1H, *Pyr-H*); 11.03 (s, 1H, NH exchangeable with D_2O); FT-IR (KBr, v,cm^{-1}): 3466-3208 (broad, OH+NH), 1670(C=O), 1633 (CH=N) cm^{-1}. MS (m/z): 578 (M^+, 10). Anal.calcd. for $C_{33}H_{30}N_4O_6$: C, 68.50; H, 5.23; N, 9.68; found C, 68.57; H, 5.22; N, 9.61.

Synthesis of 2-(1,2,3,4,5-Penta-*O*-acetyl-D-galactopentitolyl)-5-(1-(1-oxo-4-(pyren-1-ylmethyl phthalazin-2(1*H*)-yl)ethyl) -1,3,4-oxadiazol-3(2*H*)-yl acetate (27b): Yield: 59%, m.p.: 104–105°C; ^{1}H NMR (DMSO-d$_6$, δ): 1.60 (d, 3H, J=8.0 Hz CH_3CH), 1.82, 1.92, 2.05, 2.14, 2.19, 2.32 (6s, 18H, 6CO CH_3), 3.40 (dd, J = 12.0 Hz, J = 3.1 Hz, 1H, H-5′), 4.21 (dd, J = 12.2 Hz, J = 3.1 Hz, 1H, H-5′′), 4.63 (m, 1H, H-4′),

4.70 (s, 2H, CH_2), 5.01 (q, 1H, *J* =8.1 Hz $CHCH_3$), 5.21 (m, 1H, H-3′), 5.34 (dd, *J* = 6.0 Hz, *J* = 7.1 Hz, 1H, H-2′), 5.50 (dd, *J* = 7.2 Hz, *J* = 7.7 Hz, 1H, H-1′), 5.83 (d, *J* = 7.8 Hz, 1H, oxadiazoline-H), 7.49 (m, 2 H, H-7,8, *Phthalaz-H*), 7.58 (t, 1 H, $J_{6,7}$ = 8.3, $J_{5,6}$ = 8.0 Hz, H-6, *Phthalaz-H*), 7.87 (d, 1 H, $J_{5,6}$ = 7.9 Hz, H-5, *Phthalaz-H*), 8.06-8.09 (2H, m, *Pyr-H*), 8.11 (d, 1H, $^3J_{HH}$ = 9.2 Hz, *Pyr-H*), 8.15 (d, 1H, $^3J_{HH}$ = 7.5 Hz, *Pyr-H*), 8.18-8.20 (m, 3H, *Pyr-H*), 8.30 (dd, 1H, $^3J_{HH}$ = 8.2 and $^4J_{HH}$ = 2.6 Hz, *Pyr-H*), 8.48 (d, 1H, $^3J_{HH}$ = 9.2 Hz, *Pyr-H*); ^{13}C NMR: δ 10.7(CH_3), 20.1, 20.4, 20.6, 20.8, 21.5(5 CH_3 ester), 22.2 (NAc), 37.3(CH_2), 59.5 (CH), 61.8 (C-6′), 67.6 (C-5′), 67.7 (C-4′), 68.1 (C-3′),70.5(C-2′), 85.4(CH-oxadiazole), 121.2, 121.6, 122.3, 123.1, 124.2, 125.1, 125.9, 126.2, 126.5, 127.0, 127.3, 127.5, 128.2, 128.5, 129.3, 129.9, 130.4, 131.9, 132.0, 132.5, 132.7, 133.0, 147.2 (Ar-C), 152.4 (N-C=O, phthalazine moiety), 156.3(C-oxadiazole); 167.4(NCO), 169.6, 169.8, 169.9, 170.4, 170.9(5 OCO); FT-IR (KBr, v,cm^{-1}): 1740 (C=O ester), 1680, 1667, 1615 cm^{-1}. MS (m/z): 876 (M$^+$, 8). Anal.calcd. for $C_{46}H_{44}N_4O_{14}$: C, 63.01; H, 5.06; N, 6.39; found C, 63.10; H, 5.10; N, 6.34.

Synthesis of 2-(1,2,3,4,5-Penta-*O*-acetyl-D-mannopentitolyl)-5-(1-(1-oxo-4-(pyren-1-ylmethyl phthalazin-2(1*H*)-yl)ethyl)-1,3,4-oxadiazol-3(2*H*)-yl acetate (27c): Yield: 65%, m.p.: 112–113°C; ^{1}H NMR (DMSO-d$_6$, δ): 1.73 (d, 3H, *J*=7.8 Hz CH_3CH), 1.82, 1.89, 2.00, 2.09, 2.18, 2.32 (6s, 18H, 6CH_3), 3.88 (dd, *J* = 12.0 Hz, *J* = 3.3 Hz, 1H, H-5′), 4.15 (dd, *J* = 11.8 Hz, *J* = 3.8 Hz, 1H, H-5′′), 4.65 (m, 1H, H-4′), 4.75 (s, 2H, CH_2), 5.02 (q, 1H, *J* =8.0 Hz $CHCH_3$), 5.14 (m, 1H, H-3′), 5.31 (dd, *J* = 6.0 Hz, *J* = 7.5 Hz, 1H, H-2′), 5.64 (dd, *J* = 7.2 Hz, *J* = 7.9 Hz, 1H, H-1′), 5.80 (d, *J* = 7.0 Hz, 1H, oxadiazoline-H),), 7.48 (m, 2 H, H-7,8, *Phthalaz-H*), 7.56 (t, 1 H, $J_{6,7}$ = 8.5, $J_{5,6}$ = 8.6 Hz, H-6, *Phthalaz-H*), 7.80 (d, 1 H, $J_{5,6}$ = 8.6 Hz, H-5, *Phthalaz-H*), 8.00 (t, 1H, $^3J_{HH}$ = 7.6 Hz, *Pyr-H*); 8.03-8.07 (m, 2H *Pyr-H*); 8.17 (d, 1H, $^3J_{HH}$ = 8.0 Hz, *Pyr-H*); 8.20 (d, 2H, $^3J_{HH}$ = 7.6 Hz, *Pyr-H*); 8.25 (d, 2H, $^3J_{HH}$ = 8.2 Hz, *Pyr-H*); 8.38 (dd, 1H, $^3J_{HH}$ = 8.1 and $^4J_{HH}$ = 2.5 Hz, *Pyr-H*); 8.44-8.46 (m, 1H, *Pyr-H*); ^{13}C NMR: δ 10.7(CH_3), 20.3, 20.4, 20.6, 20.7, 21.3(5 CH_3 ester), 21.9 (NAc), 37.1(CH_2), 59.8 (CH), 61.9 (C-6′), 67.5 (C-5′), 67.6 (C-4′), 68.3 (C-3′),70.6(C-2′), 86.2(CH-oxadiazole), 121.0, 121.5, 122.3, 122.9, 123.2, 124.7, 125.5, 126.2, 126.5, 127.0, 127.2, 127.4, 128.3, 128.6, 129.1, 129.5, 130.4, 131.4, 132.0, 132.5, 132.9, 133.6, 146.7 (Ar-C), 153.2 (N-C=O, phthalazine moiety), 156.0(C-oxadiazole); 168.0(NCO), 169.2, 169.7, 169.9, 170.2, 171.1(5 OCO); FT-IR (KBr, v,cm^{-1}): 1733 (C=O ester), 1677, 1668, 1616 cm^{-1}. MS (m/z): 876 (M$^+$, 10). Anal.calcd. for $C_{46}H_{44}N_4O_{14}$: C, 63.01; H, 5.06; N, 6.39; found C, 63.05; H, 5.11; N, 6.31.

Synthesis of 2-(1,2,3,4-Tetra-*O*-acetyl-D-arabinotetritolyl)-5-(1-(1-oxo-4-(pyren-1-ylmethyl) phthalazin-2(1*H*)-yl)ethyl)-1,3,4-oxadiazol-3(2*H*)-yl acetate (27d): Yield: 54%, m.p.: 77–78°C; ^{1}H NMR (DMSO-d$_6$, δ): 1.96, 1.98, 2.01, 2.06, 2.3 (5s, 15H, 5 COCH3), 3.97(dd, 1H, J = 6.6 Hz, H-4′′), 4.16(dd, 1H, J = 12.0 Hz, H-4′), 4.70 (s, 2H, CH_2), 5.01 (q, 1H, *J* =8.1 Hz $CHCH_3$), 5.22(m, 1H, J = 3.9 Hz, H-3′), 5.43(t, 1H, J = 5.1 Hz, H-2′), 5.52(t, 1H, J = 4.8 Hz, H-1′), 6.07(d, 1H, J = 4.2 Hz, oxadiazoline-H), 7.50 (m, 2 H, H-7,8, *Phthalaz-H*), 7.61 (t, 1 H, $J_{6,7}$ = 8.2, $J_{5,6}$ = 8.2 Hz, H-6, *Phthalaz-H*), 7.80 (d, 1 H, $J_{5,6}$ = 8.1 Hz, H-5, *Phthalaz-H*), 8.03-8.04 (m,2H, *Pyr-H*); 8.09 (d,1H, $^3J_{HH}$ = 8.9 Hz, *Pyr-H*); 8.19 (d, 1H, $^3J_{HH}$ = 7.7 Hz, *Pyr-H*); 8.20-8.23

(m, 3H, *Pyr-H*); 8.30 (dd, 1H, $^3J_{HH}$ = 8.1 and $^4J_{HH}$ = 2.8 Hz, *Pyr-H*); 8.48 (d, 1H, $^3J_{HH}$ = 9.3 Hz, *Pyr-H*); ^{13}C NMR: δ 10.7(CH_3), 20.2, 20.5, 20.9, 21.1(4 CH_3 ester), 22.0 (NAc), 37.0(CH_2), 59.1 (CH), 61.7 (C-5′), 68.5 (C-4′), 70.2 (C-3′), 71.4 (C-2′), 84.1(CH-oxadiazole), 121.3, 121.5, 122.3, 123.2, 124.5, 125.1, 125.7, 126.2, 126.6, 127.0, 127.2, 127.5, 128.1, 128.5, 129.2, 129.7, 130.4, 131.8, 132.0, 132.5, 132.9, 133.2, 146.0 (Ar-C), 154.9 (N-C=O, phthalazine moiety), 156.4(C-oxadiazole); 166.2(NCO), 169.6, 169.8, 169.9, 170.4(4 OCO); FT-IR (KBr, *v*,cm^{-1}): 1734 (C=O ester), 1689, 1667, 1617 cm^{-1}. MS (m/z): 804 (M^+, 15). Anal.calcd. for $C_{43}H_{40}N_4O_{12}$: C, 64.17; H, 5.01; N, 6.96; found C, 64.20; H, 4.96; N, 6.90.

Synthesis of 2-(1,2,3,4-Tetra-*O*-acetyl-D-xylotetritolyl)-5-(1-(1-oxo-4-(pyren-1-ylmethyl) phthalazin-2(1*H*)-yl)ethyl)-1,3,4-oxadiazol-3(2*H*)-yl acetate (27e): Yield: 60%, m.p.: 98–99°C; ^{1}H NMR (DMSO-d_6, *δ*): 1.70 (d, 3H, *J*=8.8 Hz CH_3CH), 1.91, 2.07, 2.10, 2.20, 2.33 (5s, 15H, 5CH3), 4.01 (dd, *J* = 12.0 Hz, *J* = 3.8 Hz, 1H, H-4′), 4.20 (dd, *J* = 12.0 Hz, *J* = 3.7 Hz, 1H, H-4′′), 4.71 (s, 2H, CH_2), 5.05 (q, 1H, *J* =8.5 Hz $CHCH_3$), 5.22 (m, 1H, H-3′), 5.30 (dd, *J* = 6.5 Hz, *J* = 7.5 Hz, 1H, H-2′), 5.60 (dd, *J* = 7.5 Hz, *J* = 6.4 Hz, 1H, H-1′), 5.88 (d, *J* = 7.5 Hz, 1H, oxadiazoline-H), 7.58 (t, 1 H, $J_{6,7}$ = 7.7 Hz, H-7, *Phthalaz-H*), 7.63 (d, 1 H, *J* = 7.0 Hz, H-8, *Phthalaz-H*), 7.72 (t, 1 H, *J* = 8.2 Hz, H-6, *Phthalaz-H*), 7.86 (d, 1 H, $J_{5,6}$ = 8.4 Hz, H-5, *Phthalaz-H*), 8.02 (t, 1H, $^3J_{HH}$ = 7.7 Hz, *Pyr-H*); 8.06- 8.09 (m, 2H, *Pyr-H*); 8.16 (d, 1H, $^3J_{HH}$ = 9.1 Hz, *Pyr-H*), 8.20-8.24 (m, 2H, *Pyr-H*), 8.30 (d, 2H, $^3J_{HH}$ = 8.5 Hz, *Pyr-H*), 8.46-8.49 (m, 1H *Pyr-H*); ^{13}C NMR: δ 10.7(CH_3), 20.4, 20.7, 20.8, 20.9(4 CH_3 ester), 21.8 (NAc), 36.3(CH_2), 58.2 (CH), 62.2 (C-5′), 68.8 (C-4′), 70.1 (C-3′), 71.1 (C-2′), 83.9(CH-oxadiazole), 121.0, 121.6, 122.7, 123.2, 124.1, 125.1, 125.8, 126.2, 126.6, 126.9, 127.2, 127.5, 128.0, 128.5, 129.0, 129.7, 130.4, 131.7, 132.3, 132.5, 132.9, 133.5, 147.4 (Ar-C), 154.9 (N-C=O, phthalazine moiety), 157.3(C-oxadiazole); 165.97(NCO), 169.2, 169.5, 169.7, 170.2(4 OCO), FT-IR (KBr, *v*,cm^{-1}): 1735 (C=O ester), 1690, 1666, 1612 cm^{-1}. MS (m/z): 804 (M^+, 18). Anal.calcd. for $C_{43}H_{40}N_4O_{12}$: C, 64.17; H, 5.01; N, 6.96; found C, 64.20; H, 4.95; N, 6.90.

Synthesis of 2-(1,2,3,4-Tetra-*O*-acetyl-D-ribotetritolyl)-5-(1-(1-oxo-4-(pyren-1-ylmethyl) phthalazin-2(1*H*)-yl)ethyl)-1,3,4-oxadiazol-3(2*H*)-yl acetate (27f): Yield: 59%, m.p.: 89–90°C; ^{1}H NMR (DMSO-d_6, *δ*): 1.65 (d, 3H, *J*=8.8 Hz CH_3CH), 1.90, 2.06, 2.18, 2.22, 2.38 (5s, 15H, 5CH3), 4.05 (dd, *J* = 10.1 Hz, *J* = 3.7 Hz, 1H, H-4′), 4.17 (dd, *J* = 10.2 Hz, *J* = 3.9 Hz, 1H, H-4′′), 4.70 (s, 2H, CH_2), 5.01 (q, 1H, *J* =8.1 Hz $CHCH_3$), 5.21 (m, 1H, H-3′), 5.30 (dd, *J* = 7.0 Hz, *J* = 7.5 Hz, 1H, H-2′), 5.52 (dd, *J* = 7.4 Hz, *J* = 7.0 Hz, 1H, H-1′), 5.84 (d, *J* = 7.4 Hz, 1H, oxadiazoline-H), 7.51 (t, 1 H, $J_{6,7}$ = 7.2 Hz, H-7, *Phthalaz-H*), 7.60 (d, 1 H, $J_{7,8}$ = 7.5 Hz, H-8, *Phthalaz-H*), 7.74 (t, 1 H, H-6, *Phthalaz-H*), 7.81 (d, 1 H, $J_{5,6}$ = 7.9 Hz,H-5, *Phthalaz-H*), 8.03-8.06 (m, 1H, *Pyr-H*); 8.11 (t, 1H, $^3J_{HH}$ = 7.6 Hz, *Pyr-H*); 8.20 (d, 1H, $^3J_{HH}$ = 9.0 Hz, *Pyr-H*); 8.27 (d, 1H, $^3J_{HH}$ = 9.0 Hz, *Pyr-H*); 8.32 (d, 1H, $^3J_{HH}$ = 8.4 Hz, *Pyr-H*); 8.34 (d, 1H, $^3J_{HH}$ = 7.2 Hz, *Pyr-H*); 8.41 (d, 1H, $^3J_{HH}$ = 7.4 Hz, *Pyr-H*); 8.43(d, 1H, $^3J_{HH}$ = 8.2 Hz, *Pyr-H*); 8.45-8.50 (m, 1H, *Pyr-H*); ^{13}C NMR: δ 11.1(CH_3), 20.0, 20.7, 21.0, 21.8(4 CH_3 ester), 22.0 (NAc), 35.5(CH_2), 59.4 (CH), 60.1 (C-5′), 68.0 (C-4′), 70.3 (C-3′), 70.9 (C-2′), 80.8(CH-oxadiazole), 120.2, 121.0, 122.1, 122.7, 123.1, 124.3, 125.8, 126.2, 126.5, 126.9, 127.3, 127.9, 128.4,

128.9, 129.3, 129.8, 130.0, 132.7, 132.9, 133.5, 134.3, 134.7, 145.4 (Ar-C), 156.5 (N-C=O, phthalazine moiety), 158.1(C-oxadiazole); 166.9 (NCO), 169.4, 169.9, 170.7, 171.2(4 OCO),FT-IR (KBr, *v*,cm^{-1}): 1738 (C=O ester), 1686, 1668, 1615 cm^{-1}; MS (m/z): 804 (M^+, 5). Anal.calcd. for $C_{43}H_{40}N_4O_{12}$: C, 64.17; H, 5.01; N, 6.96; found C, 64.22; H, 4.92; N, 6.92.

Synthesis of 4-((4-(Pyren-1-ylmethyl)phthalazin-1-yl)selanyl)butyl acetate (28a): Yield: 75%, m.p.: 122–123°C; ^{1}H NMR (DMSO-d_6, δ):1.60 (m, 2H, CH_2), 1.85 (m, 2H, CH_2), 2.08 (s, 3H, CH_3CO), 4.03 (t, 2H, *J* = 6.5 Hz, $SeCH_2$), 4.57 (t, 2H, *J* = 6.5 Hz, CH_2OCO), 4.71 (s, 2H, CH_2), 7.55 (t, 1 H, $J_{6,7}$ = 7.8 Hz, H-7, *Phthalaz-H*), 7.60 (d, 1 H, *J* = 7.8 Hz, H-8, *Phthalaz-H*), 7.83 (t, 1 H, *J* = 8.5 Hz, H-6, *Phthalaz-H*), 7.87 (d, 1 H, $J_{5,6}$ = 8.8 Hz, H-5, *Phthalaz-H*), 8.01 (t, 1H $^3J_{HH}$ = 7.8 Hz, *Pyr-H*), 8.13 (d, 1H $^3J_{HH}$ = 9.1 Hz, *Pyr-H*), 8.17 (d, 1H $^3J_{HH}$ = 9.0 Hz, *Pyr-H*), 8.21 (d, 1H $^3J_{HH}$ = 8.7 Hz, *Pyr-H*), 8.22-8.25 (m, 3H *Pyr-H*), 8.42 (d, 1H $^3J_{HH}$ = 8.1 Hz, *Pyr-H*), 8.49 (d, 1H $^3J_{HH}$ = 9.5 Hz, *Pyr-H*); ^{13}C NMR: δ 20.8(CH_3 ester), 24.7(CH_2), 30.1(CH_2), 34.3($SeCH_2$), 37.0(CH_2), 67.9(CH_2O), 121.2, 121.7, 122.3, 122.8, 123.1, 124.5, 125.3, 126.1, 126.4, 127.0, 127.4, 127.5, 128.0, 128.6, 129.0, 129.3, 130.4, 131.5, 132.0, 132.7, 132.9, 133.1, 144.1 (Ar-C), 153.2 (N-C=O, phthalazine moiety), 173.8(OCO); FT-IR (KBr, *v*, cm^{-1}): 1739 (C=O ester) cm^{-1}; MS (m/z): 537 (M^+, 21). Anal.calcd. for $C_{31}H_{26}N_2O_2Se$: C, 69.27; H, 4.88; N, 5.21; found C, 69.31; H, 4.85; N, 5.19.

Synthesis of 4-((4-(Pyren-1-ylmethyl)phthalazin-1-yl)thio)butyl acetate (28b): Yield: 71%, m.p.: 130–131°C; ^{1}H NMR (DMSO-d_6, δ):1.70 (m, 2H, CH_2), 1.91 (m, 2H, CH_2), 2.02 (s, 3H, CH_3CO), 4.14 (t, 2H, *J* = 6. 5 Hz, SCH_2), 4.71 (s, 2H, CH_2), 5.10 (t, 2H, *J* = 7. 0 Hz, CH_2OCO), 7.48 (m, 2 H, H-7,8, *Phthalaz-H*), 7.60 (t, 1 H, $J_{6,7}$ = 7.5 $J_{5,6}$ = 7.8 Hz, H-6, *Phthalaz-H*), 7.84 (d, 1 H, $J_{5,6}$ = 7.7 Hz, H-5, *Phthalaz-H*), 8.01 (t, 1H, $^3J_{HH}$ = 7.6 Hz, *Pyr-H*), 8.03-8.06 (m, 2H, *Pyr-H*), 8.24 (d, 1H, $^3J_{HH}$ = 9.0 Hz, *Pyr-H*), 8.27(d, 2H, $^3J_{HH}$ = 9.2 Hz, *Pyr-H*), 8.30 (d, 1H, $^3J_{HH}$ = 8.1 Hz, *Pyr-H*), 8.40 (d, 1H, $^3J_{HH}$ = 9.4 Hz, *Pyr-H*), 8.44-8.47 (m, 1H, *Pyr-H*); ^{13}C NMR: δ 21.0(CH_3 ester), 24.8(CH_2), 30.3(CH_2), 36.1(SCH_2), 37.2(CH_2), 67.6(CH_2O), 121.0, 121.5, 122.1, 122.7, 123.6, 124.5, 125.0, 126.3, 126.4, 127.1, 127.4, 127.5, 128.2, 128.6, 129.1, 129.3, 130.2, 131.4, 132.2, 132.7, 132.9, 133.4, 145.2 (Ar-C), 156.0 (N-C=O, phthalazine moiety), 172.4(OCO); FT-IR (KBr, *v*, cm^{-1}): 1740 (C=O ester) cm^{-1}. MS (m/z): 490 (M^+, 33). Anal.calcd. for $C_{31}H_{26}N_2O_2S$: C, 75.89; H, 5.34; N, 5.71; S, 6.53; found C, 75.89; H, 5.39; N, 5.76; S, 6.50.

Synthesis of 4-((4-(Pyren-1-ylmethyl)phthalazin-1-yl)selanyl)butan-1-ol (29a): Yield: 60%, m.p.: 166–167°C; ^{1}H NMR (DMSO-d_6, δ):1.54 (m, 2H, CH_2), 1.78 (m, 2H, CH_2), 3.91 (t, 2H, *J* = 6.2 Hz, $SeCH_2$), 4.39 (t, 2H, *J* = 6.5 Hz, CH_2O), 4.74 (s, 2H, CH_2), 4.83 (br, 1H, D_2O exchangeable OH), 7.54(t, 1 H, $J_{6,7}$ = 7.0 Hz, H-7, *Phthalaz-H*), 7.59 (d, 1 H, $J_{7,8}$ = 7.5 Hz, H-8, *Phthalaz-H*), 7.70 (t, 1 H, H-6, *Phthalaz-H*), 7.84 (d, 1 H, $J_{5,6}$ = 8.5 Hz, H-5, *Phthalaz-H*), 8.04-8.07 (m, 2H, *Pyr-H*); 8.11 (d, 1H, $^3J_{HH}$ = 8.1 Hz, *Pyr-H*); 8.18 (d, 1H, $^3J_{HH}$ = 7.3 Hz, *Pyr-H*); 8.21-8.24 (m, 3H, *Pyr-H*); 8.33 (dd, 1H, $^3J_{HH}$ = 8.2 and $^4J_{HH}$ = 2.6 Hz, *Pyr-H*); 8.47 (d, 1H, $^3J_{HH}$ = 9.1 Hz, *Pyr-H*); ^{13}C NMR: δ 24.8 (CH_2), 30.2 (CH_2), 35.1 ($SeCH_2$), 37.1(CH_2), 64.0(CH_2OH), 121.3, 121.5, 122.4, 122.7, 123.7, 124.5, 125.3, 126.3, 126.7, 127.1, 127.7, 127.9, 128.2, 128.7, 129.0, 129.3, 130.3, 131.4,

132.5, 132.7, 132.8, 133.4, 143.4 (Ar-C), 156.0 (N-C=O, phthalazine moiety); FT-IR (KBr, v, cm^{-1}): 3431-3122 (OH) cm^{-1}; MS (m/z): 495 (M^+, 13). Anal.calcd. for $C_{29}H_{24}N_2OSe$: C, 70.30; H, 4.88; N, 5.65; found C, 70.35; H, 4.80; N, 5.61.

Synthesis of 4-((4-(Pyren-1-ylmethyl)phthalazin-1-yl)thio)butan-1-ol (29b): Yield: 71%, m.p.: 165–166°C; ^{1}H NMR (DMSO-d$_6$, δ): 1.60 (m, 2H, CH_2), 2.04 (m, 2H, CH_2), 4.15 (t, 2H, J = 6.70 Hz, SCH_2), 4.72 (s, 2H, CH_2), 5.22 (t, 2H, J = 6.80 Hz, CH_2O), 5.30 (br, 1H, D_2O exchangeable OH), 7.52 (m, 2 H, H-7,8, *Phthalaz-H*), 7.64 (t, 1 H, $J_{6,7}$ = 8.3, $J_{5,6}$ = 8.3 Hz, H-6, *Phthalaz-H*), 7.84 (d, 1 H, $J_{5,6}$ = 8.1 Hz, H-5, *Phthalaz-H*), 8.05-8.07 (m,2H, *Pyr-H*); 8.09 (d,1H, $^3J_{HH}$ = 8.7 Hz, *Pyr-H*); 8.19 (d, 1H, $^3J_{HH}$ = 7.5 Hz, *Pyr-H*); 8.20-8.24 (m, 3H, *Pyr-H*); 8.31 (dd, 1H, $^3J_{HH}$ = 8.1 and $^4J_{HH}$ = 2.8 Hz, *Pyr-H*); 8.45 (d, 1H, $^3J_{HH}$ = 9.0 Hz, *Pyr-H*); ^{13}C NMR: δ 25.3 (CH_2), 30.4 (CH_2), 36.5 (SCH_2), 37.5(CH_2), 63.4(CH_2OH), 121.4, 121.7, 122.4, 122.7, 123.3, 124.5, 125.2, 126.3, 126.8, 127.1, 127.6, 128.1, 128.2, 128.8, 129.0, 129.2, 130.3, 131.6, 132.5, 132.6, 132.8, 133.5, 144.7 (Ar-C), 153.1 (N-C=O, phthalazine moiety); FT-IR (KBr, v, cm^{-1}): 3487-3187 (OH) cm^{-1}; MS (m/z): 448 (M^+, 10). Anal.calcd. for $C_{29}H_{24}N_2OS$: C, 77.65; H, 5.39; N, 6.24; S, 7.15; found C, 77.69; H, 5.43; N, 6.24; S, 7.10.

Synthesis of 2-(((4-(pyren-1-ylmethyl)phthalazin-1-yl)selanyl)methoxy)ethyl acetate (30a). : Yield: 60%, m.p.: 155–156°C; ^{1}H NMR (DMSO-d$_6$, δ): 2.12 (s, 3H, $OCOCH_3$), 3.80 (t, 2H, J = 5.0 Hz, OCH_2), 4.27 (t, 2H, J = 7.0 Hz, CH_2OCO), 4.40 (s, 2H, $SeCH_2O$), 4.68 (s, 2H, CH_2), 7.48 (t, 1 H, $J_{6,7}$ = 7.8 Hz, H-7, *Phthalaz-H*), 7.64 (d, 1 H, J = 7.7 Hz, H-8, *Phthalaz-H*), 7.72 (t, 1H, J = 8.5 Hz, H-6, *Phthalaz-H*), 7.87 (d, 1 H, $J_{5,6}$ = 8.6 Hz, H-5, *Phthalaz-H*), 8.01 (t, 1H, $^3J_{HH}$ = 7.5 Hz, *Pyr-H*); 8.04- 8.07 (m, 2H, *Pyr-H*); 8.18 (d, 1H, $^3J_{HH}$ = 9.1 Hz, *Pyr-H*), 8.21-8.24 (m, 2H, *Pyr-H*), 8.32 (d, 2H, $^3J_{HH}$ = 8.3 Hz, *Pyr-H*), 8.46-8.50 (m, 1H *Pyr-H*); ^{13}C NMR: δ 21.0 (CH_3 ester), 37.2(CH_2), 68.3(CH_2OAc), 70.0(OCH_2), 75.9($SeCH_2$), 121.1, 121.4, 122.2, 122.7, 123.4, 124.5, 125.0, 126.2, 126.5, 127.2, 127.9, 128.2, 128.4, 128.8, 129.3, 129.6, 130.1, 131.6, 132.2, 132.6, 132.9, 133.6, 142.7 (Ar-C), 153.2 (N-C=O, phthalazine moiety), 177.1(CO); FT-IR (KBr, v, cm^{-1}): 1738(C=O ester) cm^{-1}; MS (m/z): 539 (M^+, 8). Anal. calcd. for $C_{30}H_{24}N_2O_3Se$: C, 66.79; H, 4.48; N, 5.19; found C, 66.83; H, 4.50; N, 5.14.

Synthesis of 2-(((4-(pyren-1-ylmethyl)phthalazin-1-yl)thio)methoxy)ethyl acetate (30b): Yield: 60%, m.p.: 120–121°C; ^{1}H NMR (DMSO-d$_6$, δ):2.16 (s, 3H, CH_3CO), 4.05 (t, 2H, J = 6.0 Hz, OCH_2), 4.88 (t, 2H, J = 8.0 Hz, CH_2OCO), 4.72 (s, 2H, CH_2), 5.22 (s, 2H, SCH_2O), 7.54 (t, 1H, $J_{6,7}$ = 7.0 Hz, H-7, *Phthalazi-H*), 7.63 (d, 1H, $J_{7,8}$ = 7.1 Hz, H-8, *Phthalaz-H*), 7.84 (t, 1H, H-6, *Phthalaz-H*), 7.96 (d, 1H, $J_{5,6}$ = 8.1 Hz, H-5, *Phthalaz-H*), 8.04 (dd, 1H, $^3J_{HH}$ = 7.2 and 7.1 Hz, *Pyr-H*), 8.05-8. 9 (m, 2H, *Pyr-H*), 8.20 (d, 1H, $^3J_{HH}$ = 9.0 Hz, *Pyr-H*), 8.27 (d, 2H, $^3J_{HH}$ = 8.0 Hz, *Pyr-H*), 8.32 (d, 1H, $^3J_{HH}$ = 8.6 Hz, *Pyr-H*), 8.43-8.48 (m, 2H, *Pyr-H*), ^{13}C NMR: δ 20.87(CH_3 ester), 37.1(CH_2), 68.3(CH_2OAc), 70.2(OCH_2), 75.9(SCH_2), 121.1, 121.4, 122.2, 122.7, 123.4, 124.5, 125.0, 126.2, 126.5, 127.2, 127.9, 128.2, 128.4, 128.8, 129.3, 129.6, 130.1, 131.6, 132.2, 132.6, 132.9, 133.6, 142.7 (Ar-C), 154.12 (N-C=O, phthalazine moiety),176.3(CO); FT-IR (KBr, v, cm^{-1}): 1747(C=O ester)

cm^{-1}. MS (m/z): 492 (M^+, 22). Anal. calcd. for $C_{30}H_{24}N_2O_3S$: C, 73.15; H, 4.91; N, 5.69; S, 6.51; found C, 73.10; H, 4.88; N, 5.60; S, 6.49.

Synthesis of 2-(((4-(Pyren-1-ylmethyl)phthalazin-1-yl)selanyl)methoxy)ethan-1-ol (31a): Yield: 66%, m.p.: 188–189°C; ^{1}H NMR (DMSO-d$_6$, δ): 3.55 (t, 2H, *J* = 6.0 Hz, OCH_2), 3.91 (t, 2H, *J* = 6.0 Hz, CH_2OH), 4.50 (s, 2H, $SeCH_2O$), 4.78(s, 2H, CH_2), 4.93 (br, 1H, D_2O exchangeable OH), 7.56 (m, 2 H, H-7,8, *Phthalaz-H*), 7.67 (t, 1 H, $J_{6,7}$ = 8.4, $J_{5,6}$ = 8.3 Hz, H-6, *Phthalaz-H*), 7.81 (d, 1 H, $J_{5,6}$ = 7.8 Hz, H-5, *Phthalaz-H*), 8.01 (t, 1H, $^3J_{HH}$ = 7.5 Hz, *Pyr-H*), 8.06-8.10 (m, 2H, *Pyr-H*), 8.15 (d, 1H $^3J_{HH}$ = 9.1 Hz, *Pyr-H*), 8.20 (d, 2H, $^3J_{HH}$ = 7.5 Hz, *Pyr-H*), 8.24 (d, 1H, $^3J_{HH}$ = 8.1 Hz, *Pyr-H*), 8.42 (d, 1H, $^3J_{HH}$ = 7.5 Hz, *Pyr-H*), 8.48 (d, 1H, $^3J_{HH}$ = 8.1 Hz, *Pyr-H*); ^{13}C NMR: δ 34.9(CH_2), 60.9 (CH_2OH), 75.0 (OCH_2), 82.10 ($SeCH_2$), 120.1, 120.9, 121.1, 122.0, 123.1, 123.4, 125.3, 126.0, 126.5, 127.0, 127.9, 128.3, 129.0, 129.8, 130.3, 130.6, 131.1, 131.9, 132.4, 132.8, 133.8, 134.0, 141.4 (Ar-C), 150.8 (N-C=O, phthalazine moiety); FT-IR (KBr, *v*, cm^{-1}): 3443-3190 (OH) cm^{-1}; MS (m/z): 497 (M^+, 11). Anal. calcd. for $C_{28}H_{22}N_2O_2Se$: C, 67.61; H, 4.46; N, 5.63; found C, 67.67; H, 4.40; N, 5.60.

Synthesis of 2-(((4-(Pyren-1-ylmethyl)phthalazin-1-yl)thio)methoxy)ethan-1-ol (31b): Yield: 69%, m.p.: 199–200°C; ^{1}H NMR (DMSO-d$_6$, δ): 3.66 (t, 2H, *J* = 6.8 Hz, OCH_2), 4.60 (t, 2H, *J* = 6.0 Hz, CH_2OH), 4.75(s, 2H, CH_2), 4.92 (s, 2H, SCH_2O), 5.03 (br, 1H, D_2O exchangeableOH),), 7.51 (t, 1 H, $J_{6,7}$ = 7.2 Hz, H-7, *Phthalaz-H*), 7.58 (d, 1 H, $J_{7,8}$ = 7.1 Hz, H-8, *Phthalaz-H*), 7.69 (t, 1 H, H-6, *Phthalaz-H*), 7.87 (d, 1 H, $J_{5,6}$ = 7.7 Hz,H-5, *Phthalaz-H*), 7.99-8.03 (m, 1H, *Pyr-H*); 8.09 (t, 1H, $^3J_{HH}$ = 7.6 Hz, *Pyr-H*); 8.22 (d, 1H, $^3J_{HH}$ = 9.1 Hz, *Pyr-H*); 8.28 (d, 1H, $^3J_{HH}$ = 9.1 Hz, *Pyr-H*); 8.33 (d, 1H, $^3J_{HH}$ = 8.7 Hz, *Pyr-H*); 8.38 (d, 1H, $^3J_{HH}$ = 7.2 Hz, *Pyr-H*); 8.41 (d, 1H, $^3J_{HH}$ = 7.6 Hz, *Pyr-H*); 8.48 (d, 1H, $^3J_{HH}$ = 8.2 Hz, *Pyr-H*); 8.50-8.53 (m, 1H, *Pyr-H*), ^{13}C NMR: δ 36.9(CH_2), 62.4 (CH_2OH), 76.1 (OCH_2), 88.12 (SCH_2), 121.0, 121.5, 122.1, 122.5, 123.8, 124.1, 125.3, 126.2, 126.6, 127.2, 127.8, 128.2, 128.4, 128.9, 129.3, 129.5, 130.1, 131.7, 132.2, 132.6, 132.8, 133.6, 144.2 (Ar-C), 153.2 (N-C=O, phthalazine moiety); FT-IR (KBr, *v*, cm^{-1}): 3453-3280 (OH) cm^{-1}; MS (m/z): 450 (M^+, 19). Anal. calcd. for $C_{28}H_{22}N_2O_2S$: C, 74.64; H, 4.92; N, 6.22; S, 7.12; found C, 74.70; H, 4.90; N, 6.22; S, 7.08.

Synthesis of 3-((4-(Pyren-1-ylmethyl)phthalazin-1-yl)selanyl)propan-1-ol (32a): Yield: 70%, m.p.: 191–192 °C; ^{1}H NMR (DMSO-d$_6$, δ): 1.82 (m, 2H, CH_2), 3.50 (t. 2H, *J* = 6.0 Hz, CH_2), 4.39 (t, 2H,*J* = 6.4 Hz, CH_2), 4.72(s, 2H, CH_2), 4.79 (s, 1H, OH exchangeable with D_2O), 7.56 (t, 1 H, $J_{6,7}$ = 7.7 Hz, H-7, *Phthalaz-H*), 7.64 (d, 1 H, *J* = 7.2 Hz, H-8, *Phthalaz-H*), 7.70 (t, 1 H, *J* = 8.0 Hz, H-6, *Phthalaz-H*), 7.91 (d, 1 H, $J_{5,6}$ = 8.1 Hz, H-5, *Phthalaz-H*), 8.03 (t, 1H, $^3J_{HH}$ = 7.3 Hz, *Pyr-H*); 8.05- 8.07 (m, 2H, *Pyr-H*); 8.17 (d, 1H, $^3J_{HH}$ = 9.0 Hz, *Pyr-H*), 8.22-8.26 (m, 2H, *Pyr-H*), 8.29 (d, 2H, $^3J_{HH}$ = 8.2 Hz, *Pyr-H*), 8.44-8.47 (m, 1H *Pyr-H*); ^{13}C NMR: δ 23.3(CH_2), 27.5($SeCH_2$), 37.1(CH_2), 67.2(CH_2OH), 120.9, 121.4, 122.2, 122.6, 123.6, 124.2, 125.3, 126.4, 126.6, 127.0, 127.8, 128.0, 128.4, 128.7, 129.3, 129.7, 130.2, 131.6, 132.2, 132.5, 132.8, 133.3, 146.2 (Ar-C), 152.2 (N-C=O, phthalazine moiety); FT-IR (KBr, *v*, cm^{-1}): 3459-3209 (OH) cm^{-1}. MS (m/z): 482 (M^+, 30). Anal. calcd. for $C_{28}H_{22}N_2OSe$: C, 69.85; H, 4.61; N, 5.82; found C, 69.90; H, 4.66; N, 5.78.

Synthesis of 3-((4-(Pyren-1-ylmethyl)phthalazin-1-yl)thio)propan-1-ol (32b): Yield: 60%, m.p.: 215-216 °C; ^{1}H NMR (DMSO-d$_6$, δ):2.02 (m, 2H, CH$_2$(g)), 3.66 (t. 2H, *J* = 6.5 Hz, CH$_2$(f)), 4.58 (t, 2H, *J* = 6.7 Hz, CH$_2$(h)), 4.76(s, 2H, CH$_2$), 4.99 (s, 1H, OH exchangeable with D$_2$O), 7.44 (m, 2 H, H-7,8, *Phthalaz-H*), 7.63 (t, 1 H, $J_{6,7}$ = 7.6, $J_{5,6}$ = 7.6 Hz, H-6, *Phthalaz-H*), 7.82 (d, 1 H, $J_{5,6}$ = 7.7 Hz, H-5, *Phthalaz-H*), 8.00-8.03 (m, 2H, *Pyr-H*), 8.18 (d, 1H, $^3J_{HH}$ = 9.0 Hz, *Pyr-H*), 8.24 (d, 2H, $^3J_{HH}$ = 9.1 Hz, *Pyr-H*), 8.29 (d, 1H, $^3J_{HH}$ = 8.2 Hz, *Pyr-H*), 8.46 (d, 1H, $^3J_{HH}$ = 9.1 Hz, *Pyr-H*), 8.48-8.51 (m, 1H, *Pyr-H*); ^{13}C NMR: δ 25.0(CH$_2$), 30.1(SCH$_2$), 37.3(CH$_2$), 68.1(CH$_2$OH), 121.3, 121.4, 122.5, 122.6, 123.6, 124.5, 125.7, 126.6, 126.9, 127.0, 127.8, 128.2, 128.4, 128.7, 129.4, 129.7, 130.5, 131.6, 132.3, 132.5, 132.9, 133.5, 147.1 (Ar-C), 156.4 (N-C=O, phthalazine moiety); FT-IR (KBr, *v*, cm^{-1}): 3390-3241 (OH) cm^{-1}; MS (m/z): 434 (M$^+$, 15). Anal. calcd. For $C_{28}H_{22}N_2OS$: C, 77.39; H, 5.10; N, 6.45; S, 7.38; found C, 77.44; H, 5.18; N, 6.41; S, 7.32.

Synthesis of 1-Chloro-3-((4-(pyren-1-ylmethyl)phthalazin-1-yl)selanyl)propan-2-ol (33a): Yield: 69%, m.p.: 186–187°C; ^{1}H NMR (DMSO-d$_6$, δ): 3.02 (m, 1H, H(g)), 3.18 (m. 1H, H(f)), 3.55 (m, 1H, H(j)), 3.71(m, 1H, H(i)), 3.92 (m, 1H, H(h)), 4.73(s, 2H, CH$_2$), 4.97 (s, 1H, D$_2$O exchangeable OH), 7.49 (t, 1 H, $J_{6,7}$ = 7.4 Hz, H-7, *Phthalaz-H*), 7.55 (d, 1 H, $J_{7,8}$ = 7.5 Hz, H-8, *Phthalaz-H*), 7.63 (t, 1 H, H-6, *Phthalaz-H*), 7.83 (d, 1 H, $J_{5,6}$ = 8.0 Hz, H-5, *Phthalaz-H*), 8.02 (t, 1H, $^3J_{HH}$ = 7.5 Hz, *Pyr-H*), 8.08-8.11 (m, 3H, *Pyr-H*), 8.21-8.24 (m, 3H, *Pyr-H*), 8.29 (d, 1H, $^3J_{HH}$ = 8.5 Hz, *Pyr-H*), 8.42-8.47 (m, 1H, *Pyr-H*), ^{13}C NMR: δ 40.6 (SeCH$_2$), 36.7(CH$_2$), 47.2(CH$_2$Cl),68.3(CHOH), 120.0, 120.7, 122.0, 122.5, 122.8, 123.1, 124.5, 126.1, 126.9, 127.1, 127.9, 128.2, 128.8, 129.3, 129.9, 130.2, 130.5, 131.8, 132.2, 132.6, 132.9, 133.4, 142.3 (Ar-C), 150.4 (N-C=O, phthalazine moiety); FT-IR (KBr, *v*, cm^{-1}): 3432-3198 (OH) cm^{-1}; MS (m/z): 515 (M$^+$, 9). Anal. calcd. for $C_{28}H_{21}ClN_2OSe$: C, 65.19; H, 4.10; Cl, 6.87; N, 5.43; found C, 65.23; H, 4.15; Cl, 6.80; N, 5.40.

Synthesis of 1-Chloro-3-((4-(pyren-1-ylmethyl)phthalazin-1-yl)thio)propan-2-ol (33b): Yield: 74%, m.p.: 198–199 °C; ^{1}H NMR (DMSO-d$_6$, δ): 3.10 (m, 1H, H(g)), 3.22 (m. 1H, H(f)), 3.59 (m, 1H, H(j)), 3.84(m, 1H, H(i)), 3.98 (m, 1H, H(h)), 4.75(s, 2H, CH$_2$), 5.11 (s, 1H, D$_2$O exchangeable OH), 7.47 (t, 1 H, $J_{6,7}$ = 7.0 Hz, H-7, *Phthalaz-H*), 7.59 (d, 1 H, *J* = 7.3 Hz, H-8, *Phthalaz-H*), 7.73 (t, 1 H, *J* = 8.0 Hz, H-6, *Phthalaz-H*), 7.85 (d, 1 H, $J_{5,6}$ = 7.9 Hz, H-5, *Phthalaz-H*), 8.06 (t, 1H, $^3J_{HH}$ = 7.6 Hz, *Pyr-H*), 8.08 (d, 1H, $^3J_{HH}$ = 8.5 Hz, *Pyr-H*), 8.11 (d, 1H, $^3J_{HH}$ = 8.6 Hz, *Pyr-H*), 8.16 (d, 1H, $^3J_{HH}$ = 9.1 Hz, *Pyr-H*), 8.22- 8.24 (m, 2H, *Pyr-H*); 8.27(d, 1H, $^3J_{HH}$ = 8.1 Hz, *Pyr-H*), 8.35 (d, 1H, $^3J_{HH}$ = 9.1 Hz, *Pyr-H*), 8.44 (dd, 1H, $^3J_{HH}$ = 8.2 Hz, $^4J_{HH}$ = 2.2 Hz, *Pyr-H*); ^{13}C NMR: δ 41.6 (SCH$_2$), 37.8(CH$_2$), 49.3.1(CH$_2$Cl),70.2(CHOH), 121.0, 121.6, 122.1, 122.7, 123.2, 124.3, 125.7, 126.6, 127.0, 127.2, 127.8, 128.0, 128.4, 128.9, 129.4, 129.8, 130.5, 131.7, 132.3, 132.6, 132.9, 133.0, 145.2 (Ar-C), 155.1 (N-C=O, phthalazine moiety); FT-IR (KBr, *v*, cm^{-1}): 3455-3110 (OH) cm^{-1}. MS (m/z): 469 (M$^+$, 25). Anal.calcd. for $C_{28}H_{21}ClN_2OS$: C, 71.71; H, 4.51; Cl, 7.56; N, 5.97; S, 6.84; found C, 71.78; H, 4.58; Cl, 7.50; N, 5.93; S, 6.81.

Table S1: HOMO energy, LUMO energy, and LUMO-HOMO energy gap of compounds calculated using the DFT(B3LYP)/ 6–31+G(d) method

Compound number	*HOMO (eV)*	*LUMO (eV)*	*Gap energy HOMO-LUMO (eV)*
3	-5.72481	-1.98909	3.72
5	-5.583887	-2.32335	3.26
7	-5.41998	-2.18619	3.23
9	-5.42727	-1.71369	3.71
16a dimer	-5.37759	-2.08332	3.29
16a-dimer2	-5.16267	-2.25396	2.90
16a-dimer3	-5.22990	-0.08323	2.98
18a	-5.3001	-1.65888	3.64
20a	-5.30172	-1.64781	3.65
20a-dimer1	-5.29794	-1.64700	3.65
20a-dimer2	-5.15511	-1.79820	3.35
24a	-5.45535	-1.68804	3.67
27f	-5.50692	-1.75311	3.75
29a	-5.28363	-1.69722	3.58
29a- dimer1	-5.31765	-1.73475	3.58
29a-dimer2	-5.27715	-1.75122	3.52
29a -dimer3	-5.26527	-1.85409	3.41
33a-dimer1	-5.369814	-1.83411	3.53
33a-dimer2	-5.41701	-2.21103	3.20
33a-dimer3	-5.29389	-2.03256	3.26

Table S2: Global descriptors of chemical reactivity of the structures of some studied compounds using the DFT(B3LYP)/ 6–31+G(d) method

Compound number	*I(eV)*	*A(eV)*	*μ(eV)*	*χ(eV)*	*η(eV)*	*σ(eV)*	*ω(eV)*
3	5,72	1,98	-3,85	3,85	1,86	0,53	3,98
5	5,58	2,32	-3,95	3,95	1,63	0,61	4,79
7	5,41	2,18	-3,80	3,80	1,61	0,62	4,47
9	5,42	1,71	-3,57	3,57	1,85	0,54	3,43
16a dimer1	5,37	2,083	-3,73	3,73	1,64	0,60	4,22
16a-dimer2	5,16	2,25	-3,70	3,70	1,45	0,68	4,72
16a-dimer3	5,22	0,083	-2,65	2,65	2,57	0,38	1,37
18a	5,30	1,65	-3,47	3,47	1,82	0,54	3,32
20a	5,30	1,64	-3,47	3,47	1,82	0,54	3,30
20a-dimer1	5,29	1,64	-3,47	3,47	1,82	0,54	3,30
20a-dimer2	5,15	1,79	-3,47	3,47	1,67	0,59	3,60
24a	5,45	1,68	-3,57	3,57	1,88	0,53	3,38
27f	5,50	1,75	-3,63	3,63	1,87	0,53	3,51
29a	5,28	1,69	-3,49	3,49	1,79	0,55	3,39
29a- dimer1	5,31	1,73	-3,52	3,52	1,79	0,55	3,47
29a-dimer2	5,27	1,75	-3,51	3,51	1,76	0,56	3,50
29a -dimer3	5,26	1,85	-3,55	3,55	1,70	0,58	3,71
33a-dimer1	5,36	1,83	-3,60	3,60	1,76	0,56	3,66
33a-dimer2	5,41	2,21	-3,81	3,81	1,60	0,62	4,53
33a-dimer3	5,29	2,032	-3,66	3,66	1,63	0,61	4,11

Fig S1: 3D plots of HOMO and LUMO molecular orbitals of some compounds calculated using the DFT(B3LYP)/ 6–31+G(d) method.

Compound number	HOMO	LUMO
**3**	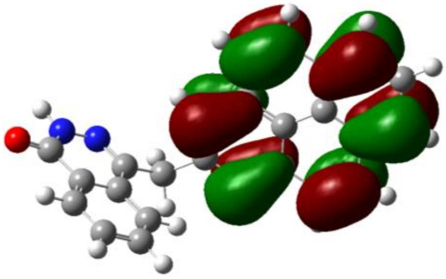	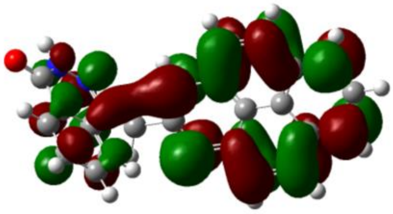
**5**	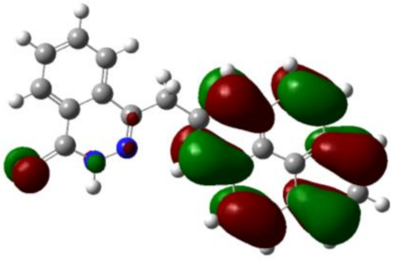	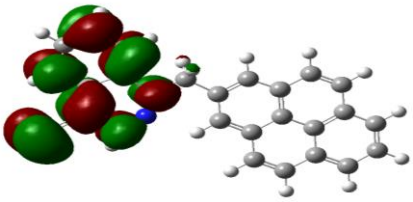
**7**	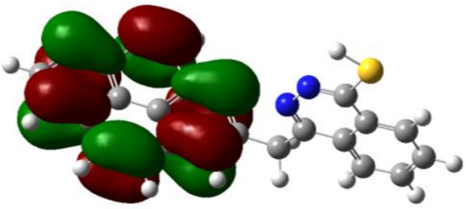	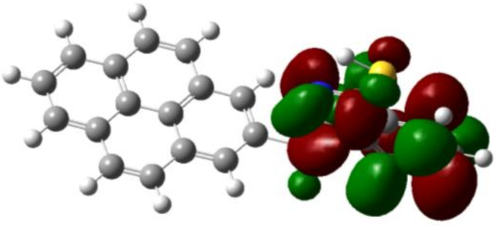
**9**	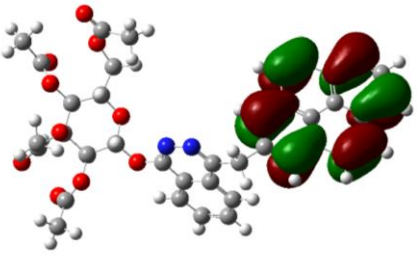	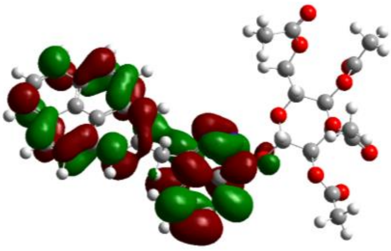

**16-a-dimer1**	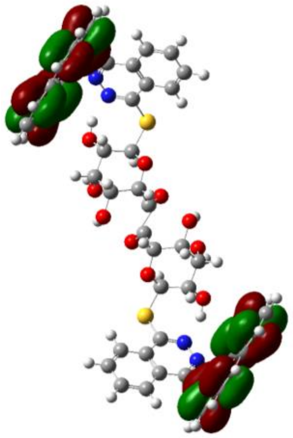	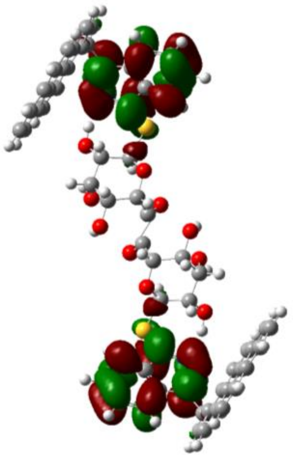
**16a-dimer2**	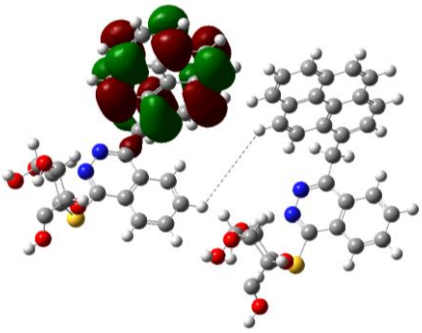	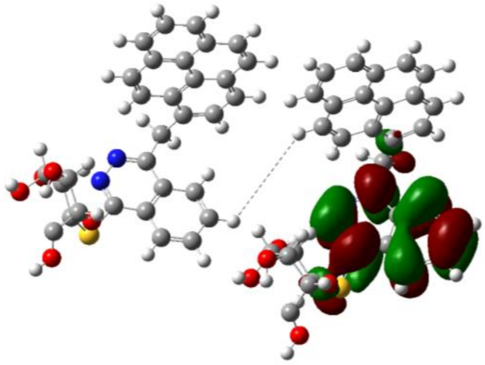
**16a-dimer3**	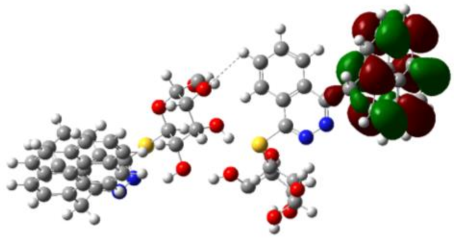	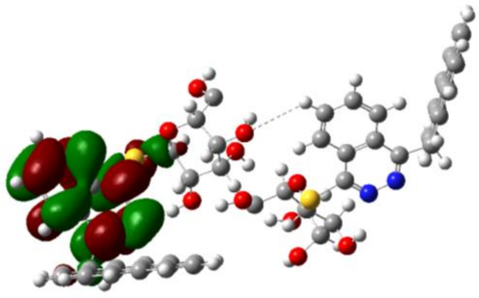
**18a**	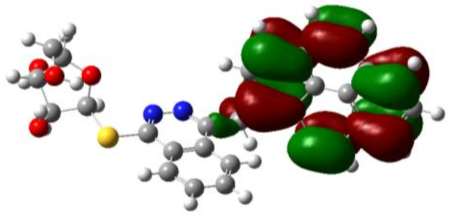	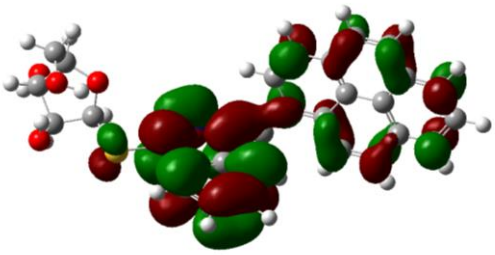
20a-dimer1		

	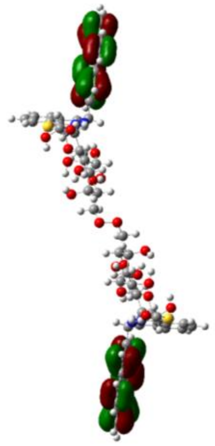	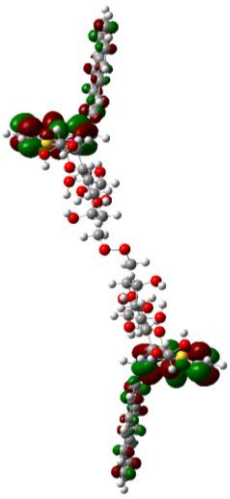
**20a-dimer2**	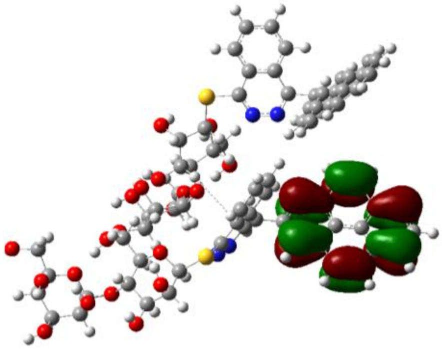	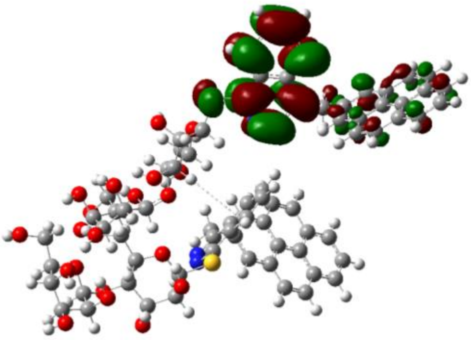
**24a**	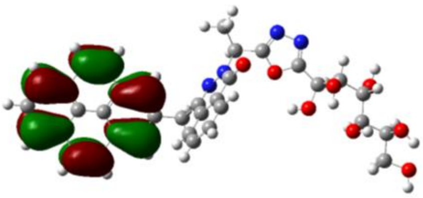	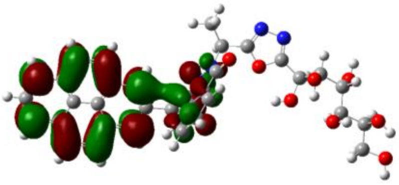
**26a**	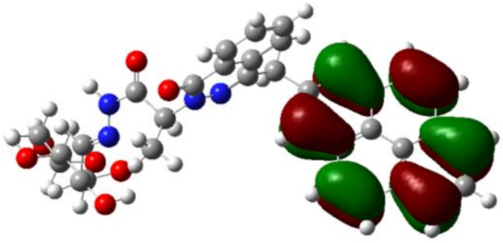	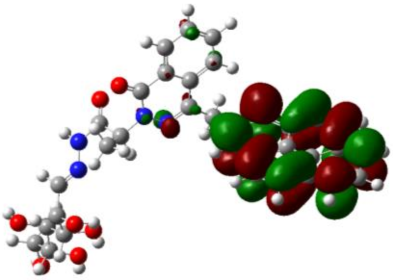

**27f**	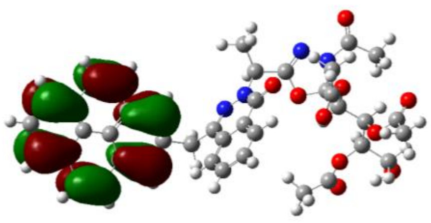	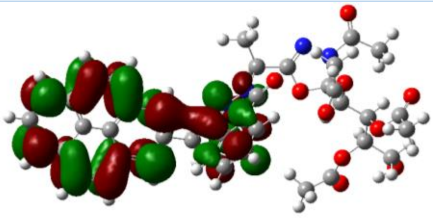
**29a-**	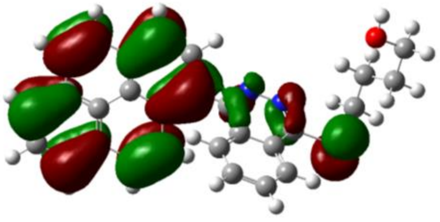	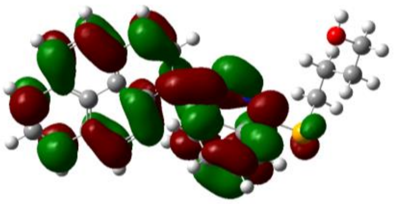
**29a-dimer1**	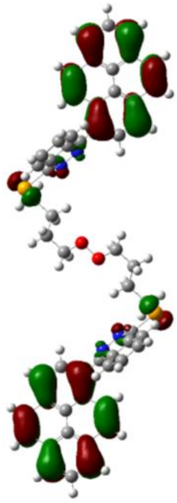	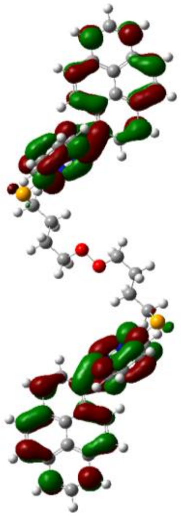
**29a-dimer2**	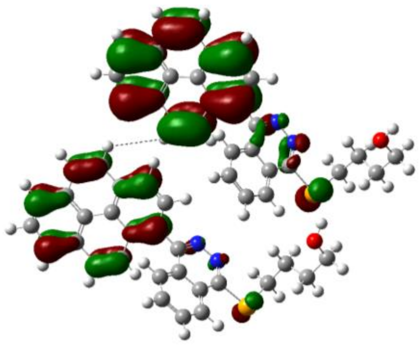	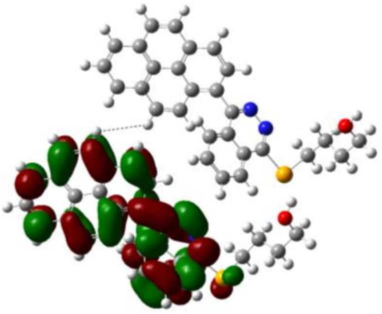
**29a-dimer3**	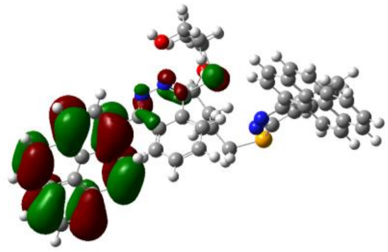	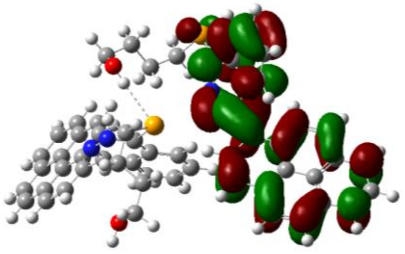

**33a-dimer1**	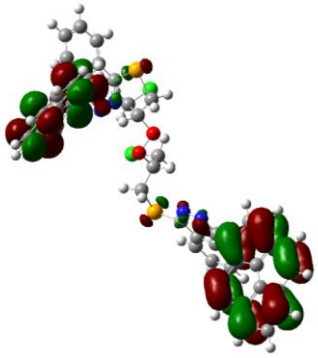	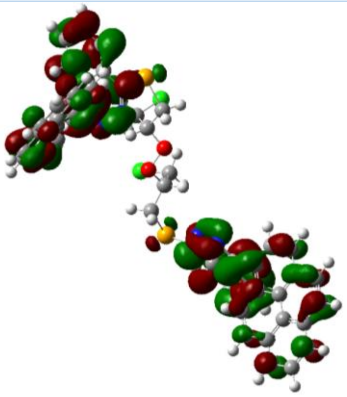
**33a-dimer2**	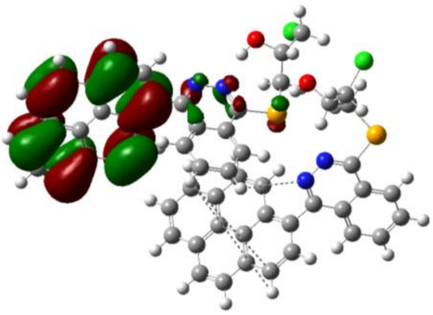	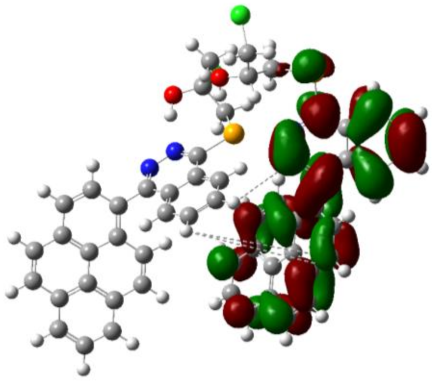
**33a-dimer3**	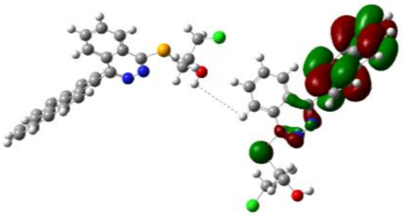	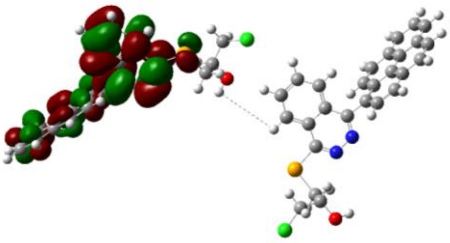

Fig.S2. Illustrative figures of molecular electrostatic potentials (MESP) mapped on the electron density surface calculated by the B3LYP/6-31+G(d)method for some selected compounds. Symbols are: O (red), C (gray), H (white), and N (blue).

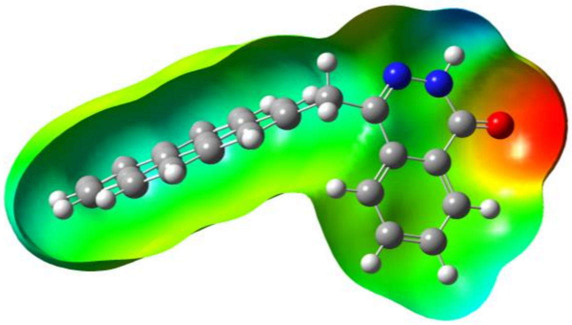

3

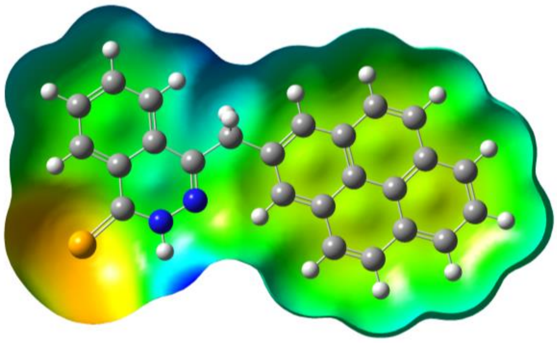

5

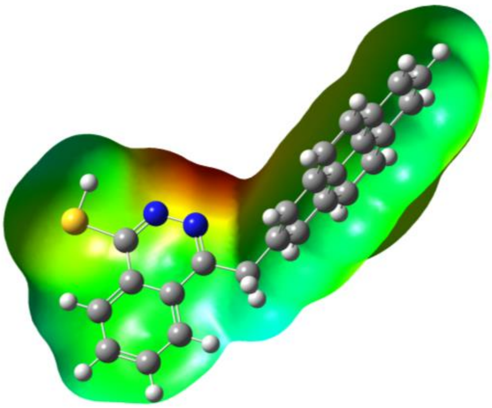

7

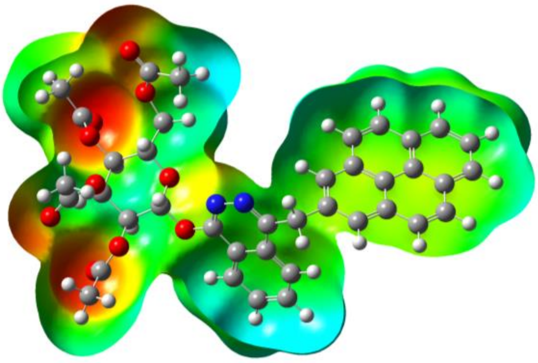

9

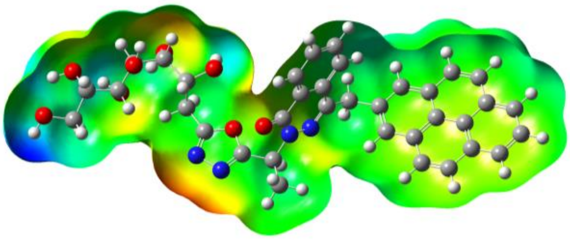

24a

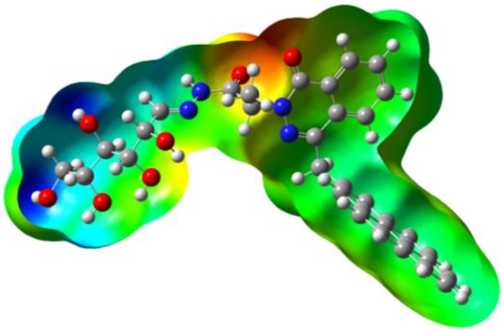

26a

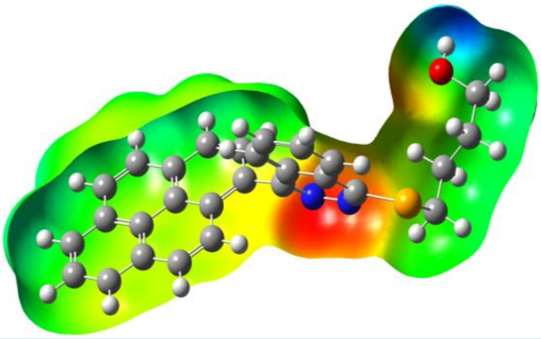

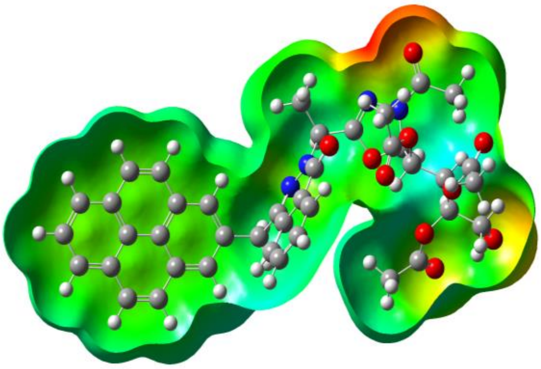 27f	**29a**
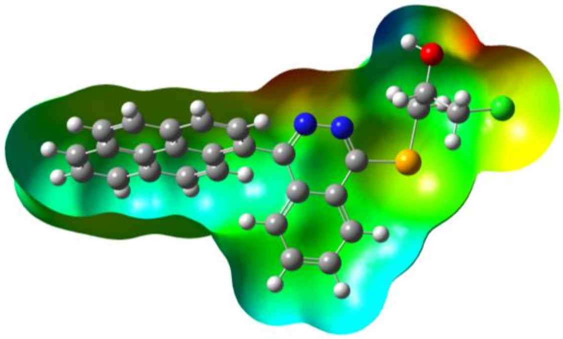 33a	
